# Phylogenetic relations and range history of jerboas of the Allactaginae subfamily (Dipodidae, Rodentia)

**DOI:** 10.1038/s41598-022-04779-x

**Published:** 2022-01-17

**Authors:** Vladimir S. Lebedev, Georgy I. Shenbrot, Boris Krystufek, Ahmad Mahmoudi, Marina N. Melnikova, Evgeniya N. Solovyeva, Alexandra A. Lisenkova, Enkhbat Undrakhbayar, Konstantin A. Rogovin, Alexey V. Surov, Anna A. Bannikova

**Affiliations:** 1 Zoological Museum of Moscow State University, B. Nikitskaya 6, 125009 Moscow, Russia; 2 A.N. Severtsov Institute of Ecology and Evolution, Russian Academy of Sciences, Leninskii Pr., 33, Moscow, Russia; 3 Science and Research Centre Koper, 6001 Koper, Slovenia; 4 Department of Biology, Faculty of Science, Urmia University, Urmia, Iran; 5 Lomonosov Moscow State University, Vorobievy Gory, 119991 Moscow, Russia; 6 Institute of General and Experimental Biology of Mongolian Academy of Science, Ulaanbaatar, 13330 Mongolia

**Keywords:** Ecology, Evolution, Zoology

## Abstract

Five-toed jerboas of the subfamily Allactaginae comprise several complex taxa occurring over a wide distribution range covering a large part of the Eurasian arid belt. In this study, we employed current methods of molecular phylogenetics based on 15 nuclear genes and the mitochondrial gene *cytb* to revise relations and systematics within Allactaginae. We also applied species distribution modelling projected on paleo-environmental data to reconstruct the geographic patterns of speciation in Allactaginae. We elucidated the intergeneric relationships within this subfamily and clarified interspecies relations within the genus *Scarturus*. Moreover, our results demonstrate the species status of *S. caprimulga*; outline the currently understudied diversity within *Orientallactaga*, *Allactaga*, and *Pygeretmus*; and improve the divergence estimates of these taxa. Based on our results from modelling of geographic range fragmentation in allactagines, we suggest the dating and location of speciation events and present hypotheses regarding general habitat niche conservatism in small mammals.

## Introduction

**Figure 1:**
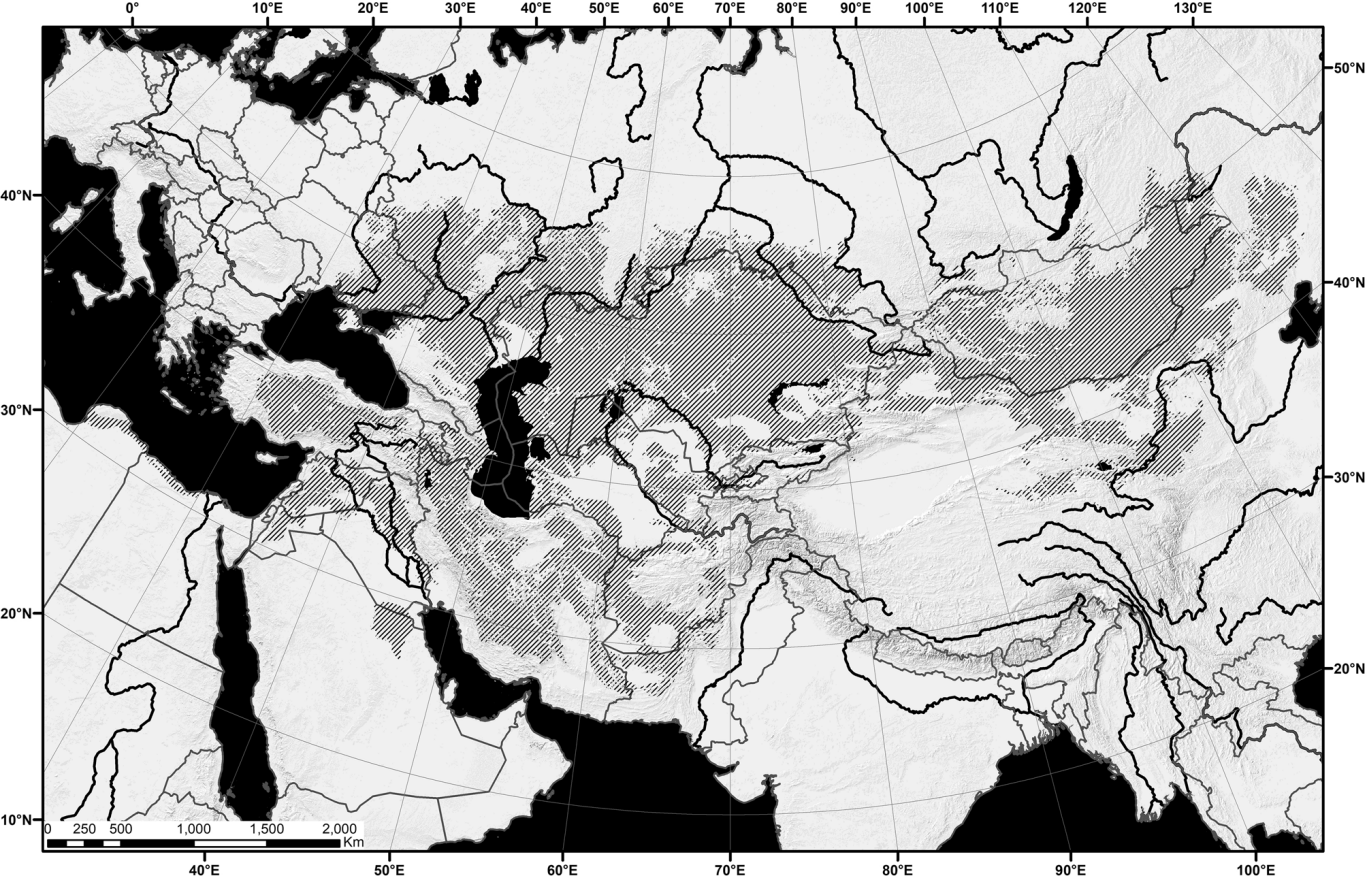
Geographic range of the Allactaginae subfamily. Hatched area corresponds to the combination of distribution ranges of all recent species (Fig. S1A–E). Distribution ranges of recent species were obtained as raster maps from modeling using MAXENT 3.4.1 software and then generated as polygon maps using ArcGIS Desktop 10.8.1 software. MAXENT: available at https://biodiversityinformatics.amnh.org/open_source/maxent/.ArcGIS Desktop: Copyright © 1995–2020 Esri.

Five-toed jerboas (Allactaginae Vinogradov, 1925) are typical elements of the Eurasian arid zone. Allactaginae is the most specious group of jerboas, the members of which have diverse diet adaptations varying from omnivorous generalists to strong folivorous specialists, and are better adapted to hard soils than other taxa. The distribution range of allactagines covers almost the entire arid (desert/semidesert) zone and parts of the steppe zone of Eurasia, stretching from eastern Tibet and southern Siberia to south-east Europe, Asia Minor, and north-east Africa, with maximum divergence in north Iran and West Central Asia and less diversity in Levant, North Africa, and East Central Asia^1,2^ (Fig. 1, Fig S1A–E).

The evolutionary history of Allactaginae started in the Early Miocene^3^ and is expected to be closely associated with the history of arid landscapes in the Palearctic. The knowledge of range evolution in Allactaginae is essential for understanding the environmental history of the arid Palearctic fauna. The biogeographic history of jerboa taxa was analysed previously^4^ using an event-based method employing a dispersal-extinction-cladogenesis model^5^. However, this and similar methods (e.g. dispersal–vicariance analysis^6^) have significant shortcomings: they use arbitrarily predefined areas, and the numbers of such areas allowed by different software programs are typically low due to computational constraints^6–8^. Moreover, these methods are based only on areas but do not take into account the environmental factors within these areas.

A geographic range is a projection of a species’ ecological niche onto a geographic space^9^. Thus, the geographic range is an expression of complex interactions between intrinsic characteristics of species such as their environmental tolerances, resource requirements, and dispersal abilities and the characteristics of their extrinsic environment^10^. Assuming that ecological tolerance and dispersal ability have a genetic basis, a species’ geographic range should be the product of two contrasting evolutionary forces—its phylogenetic history, which dictates similarities between related species, and selection or drift in contrasting environments, which causes divergence^11^. Owing to speciation processes being predominantly allopatric^12,13^, we can expect that geographic ranges of related species are more similar than would be expected by chance, reflecting both conservatism of ecological niche characteristics and dispersal abilities^14^. Peterson et al.^15^ hypothesised that ecological niches evolve only little at or around the time of speciation events whereas niche differences accumulate later; this hypothesis was supported by analysis of habitat niche evolution of arvicoline rodents^16^.

It seems that, after emergence of the main ecophysiological and morphological adaptations in allactagines, foraging niches within the group evolved (and diverged) substantially whereas habitat niches evolved relatively slow, mainly in terms of local adaptations. This relative conservatism of habitat niches allows us to implement a new approach to ancestral range estimation. Namely, we employed methods of species distribution modelling (SDM) to estimate the patterns of geographic distribution of ancient species. To accomplish this task, we obtained models for current descendants of a putative ancestral form and projected them to paleo-environment.

The knowledge of phylogeny and systematics is essential for historical range reconstructions because “the results of biogeographic analysis are true as much as taxonomic backgrounds used for the analysis are true”^9^. The current phylogeny and taxonomy of this group was substantially influenced and altered by emerging molecular data. Initially, only three genera were recognised based on morphological and cytogenetic data: *Allactaga* Cuvier, 1836*, Pygeretmus* Gloger, 1841, and *Allactodipus* Kolesnikov, 1937^17^*.* However, the earliest molecular studies suggested that the genus *Allactaga* is paraphyletic relative to *Allactodipus* and likely to *Pygeretmus*^4,18^. The most recent revision^2^ accepted five genera with a total of 16 species: *Allactaga*, *Allactodipus*, *Orientallactaga* Shenbrot, 1984, *Scarturus* Gloger, 1841, and *Pygeretmus* (with subgenera *Pygeretmus* and *Alactagulus* Nehring, 1897). However, the phylogeny of this group is understudied as previous research did not include all species, and the position of some groups (e.g. *Pygeretmus*) appeared controversial. Other studies focused on particular species groups demonstrating substantial cryptic diversity and found that some species are in fact complex species groups containing new species^18–23^. These findings require confirmation based on further genetic data and broader taxon sampling.

The aim of the present study was to examine the phylogenetic relations and taxonomic structure of Allactaginae with a focus on the correlation between recognised morphological species and genera and their genetic variability. Based on the phylogenetic results, the timing and geographic patterns of divergence among the major phylogenetic lineages were estimated.

## Materials and methods

### Taxon sampling and DNA processing

Taxonomy used in the present study follows Michaux and Shenbrot^2^ with modifications as suggested by Bannikova et al.^22^. In particular, based on the results of the latter study, we recognize three species within *S. elater* sensu lato (s.l.): *S. elater* (Lichtenstein, 1825), *S. indicus* (Gray, 1842), and *S. heptneri* (Pavlenko et Denisenko, 1976). The latter name is used provisionally. Iranian jerboas described as *Allactaga toussi*^24^ are included in *S. indicus* and not in *S. vinogradovi* (Argyropulo, 1941). The jerboa from Kopet Dag classified as *Paralactaga cf. williamsi* (Thomas, 1897) by Hamidi et al.^25^ is designated here as *Scarturus *sp. (Kopet Dag). The entire sample consists of 19 species of Allactaginae encompassing 5 genera (Supplementary Table S1).

For the DNA analysis, we used tissue samples stored in the collection of Zoological Museum of Moscow University (ZMMU) and Zoological Research Museum Alexander Koenig Leibniz Institute for Animal Biodiversity (ZFMK). The permissions to collect tissue samples from ZMMU and ZFMK were granted by the curators of the respective collections.

The original material includes sequences of 111 specimens of Allactaginae and four outgroups (*Salpingotus kozlovi* Vinogradov, 1922, *Cardiocranius paradoxus* Satunin, 1903, *Euchoreutes naso* Sclater, 1890, and *Dipus sagitta* Pallas, 1773) (Table S1). For the majority of the sample, we sequenced fragments of 15 nuclear genes and the mitochondrial *cytb* gene, while only *cytb* sequences were obtained for 46 specimens. In a few cases, DNA was extracted from the dried tissues of museum collection specimens. A total of 698 sequences of Allactaginae and outgroups were retrieved from GenBank (Table S2) and 917 sequences were obtained de novo (for Genbank accession numbers see Table S3). Details of DNA extraction, amplification, and sequencing are given in Supplementary methods (Molecular procedures and Table S4).

### Phylogenetic reconstructions

Phylogenetic reconstructions were performed with each gene separately and all nuclear genes combined. Trees were rooted using representatives of Dipodinae, Euchoreutinae, and Cardiocraniinae. In the combined analyses of 15 nuclear genes, the final alignment consisted of 10,993 bp. In total, the data set contained 64 specimens including five outgroups. Phylogenetic trees were reconstructed based on nuclear concatenation under Maximum Likelihood (ML), Maximum Parsimony (MP) and Bayesian criteria using IQTREE version 1.6^26^, PAUP* 4.0b10^27^, and MrBayes 3.2^28^, respectively. To examine the possibility that certain inferred relationships are driven by a single outlier gene, the ML analysis was re-run 15 times with one gene excluded in each iteration. Individual gene trees were generated in IQTREE and MrBayes.

The final alignment of *cytb* included 1140 bp for 664 specimens of Allactaginae and 10 outgroups. Taking into account a large number of sequences in the alignment, we restricted the analysis to the ML tree reconstruction in IQTREE and the Bayesian ultrametric tree inference in BEAST 1.10^29^.

We also used two multilocus approaches specifically designed to estimate a species tree from a set of potentially discordant loci. First, the species tree was reconstructed under multispecies coalescent model^30^ as implemented in *BEAST 1.8.4^29^. Second, a summary coalescent analysis framework as implemented in ASTRAL ver. 5.7.7^31^ was applied to the gene trees and their posterior distributions generated by MrBayes. In either case, both nuclear and mitochondrial data were used as input.

The details of the analyses are given in Supplementary methods (Phylogenetic reconstructions, Supplementary Tables S5, S6, Supplementary Fig. S2).

### Species delimitation

Boundaries between genetic lineages were identified based on mitochondrial data. This approach was utilized since the mtDNA alignment contained more sequences and provided a better representation of allactagine diversity. The *cytb* dataset is currently more informative than the nuclear concatenation of approximately 10,000 bp at species/subspecies level because of its substantially faster rate of evolution. Three species delimitation methods were employed: the Automatic Barcode Gap Discovery (ABGD) method^32^, the General Mixed Yule Coalescent (GMYC) model^33,34^, and the multi-rate Poisson Tree Process (mPTP) method^35^.

### Diversification analysis

To examine diversification dynamics in Allactaginae, we used the MEDUSA method as implemented in the *geiger* package^36^ for R, which calculates the best-fitting diversification scheme by optimising rate shifts on the phylogenetic tree. As input, we used the ultrametric trees produced using BEAST 1.10 software from the nuclear concatenation and *cytb* alignments. It was assumed that no correction for the proportion of unsampled taxa was needed. The trees were pruned to include a single representative per lineage (sensu ABGD). Considering that, in the mtDNA tree, heights for more ancient nodes are biased downwards (see “Mitochondrial results” below), the outgroups were excluded from the mitochondrial tree but were retained in the nuclear tree.

### Molecular dating

Chronograms and uncalibrated ultrametric trees were reconstructed using BEAST ver. 1.10 based on nuclear concatenation. Before the analysis, each gene was tested for departure from the strict molecular clock using a Likelihood Ratio Test implemented in PAML 4.9^37^ (for test details see Supplementary Table S7). The strict clock model was rejected regarding 12 of 15 genes; therefore, each subset was assigned a separate relaxed clock model. To test whether the results were sensitive to the choice of the relaxed clock model, the analyses were performed using both an uncorrelated clock with lognormal distribution of rates (UCLD) and a random local clock (RLC) model^38^.

To calibrate the tree, we used a set of seven fossil calibrations, each corresponding to the First Appearance Datum (FAD) of a Dipodidae taxon (Table S8). The base of the stratigraphic zone to which a FAD is allocated (FAD_min_) was used as the hard minimum constraint on the age of the split between a lineage with known FAD and its nearest sister group. Objective identification of maximum constraints (hard or soft) is methodologically problematic, thus the choice of the upper boundaries of node ages is frequently performed based on conventions such as stratigraphic bounding^39^.

In this study, to construct prior calibration densities and thereby identify soft maximum constraints on calibration points, we attempted to model distributions of ghost lineage times (i.e. the time interval between FAD and the true age of divergence (Td); = Td − FAD). It was assumed that distributions of the relative ghost lineage times (i.e. normalised by dividing by FAD) were identical for all nodes with FAD > ½Td. The mean and variance of this distribution were estimated based on the available FADs and corresponding relative split ages. The method employed for this purpose was similar to that of Marshall^40,41^; details of the calculations are provided in the Supplementary methods (Molecular dating).

### Biogeographic paleo-reconstructions

The main assumption we used in paleo-reconstructions of distribution patterns based on paleo-environmental conditions was that early stages of divergence of phylogenetic lines were due to geographic speciation. In other words, a unified geographic range of an ancestor was subdivided into isolated parts due to changes in climate, and the divergence of phylogenetic lines started in these isolated parts as a result of local adaptations and absence of gene flow among isolates. The consequence of this assumption is that species distribution model (SDM) of an ancestor and its close descendants can be based on environmental conditions in combined points of occurrences of these descendants. The events leading to divergence of two phylogenetic lines can be described by the following scenarios. The first is the classical vicariance scenario, when a unified ancestral geographic range is subdivided into isolated parts due to changes in climate, and divergence of phylogenetic lines started in these isolated parts as a result of local adaptations and the absence of gene flow among isolates. The second scenario is similar to founder-event speciation^42^. In this case, a corridor emerges between the area occupied by the ancestor and an environmentally suitable but unoccupied area, which disappears soon after, resulting in formation of an isolated founder population.

Species distribution modelling was performed using the MAXENT 3.4 software^43,44^, the details of the analysis are given in Supplementary methods (SDM).

## Results

### Concatenated nuclear data

The concatenated nuclear data produced a highly resolved and robust phylogeny. Tree topologies derived from ML, MP, and Bayesian analyses were consistent at all interspecific nodes. Most clades were highly supported by bootstrap and Bayesian credibility values.

**Figure 2:**
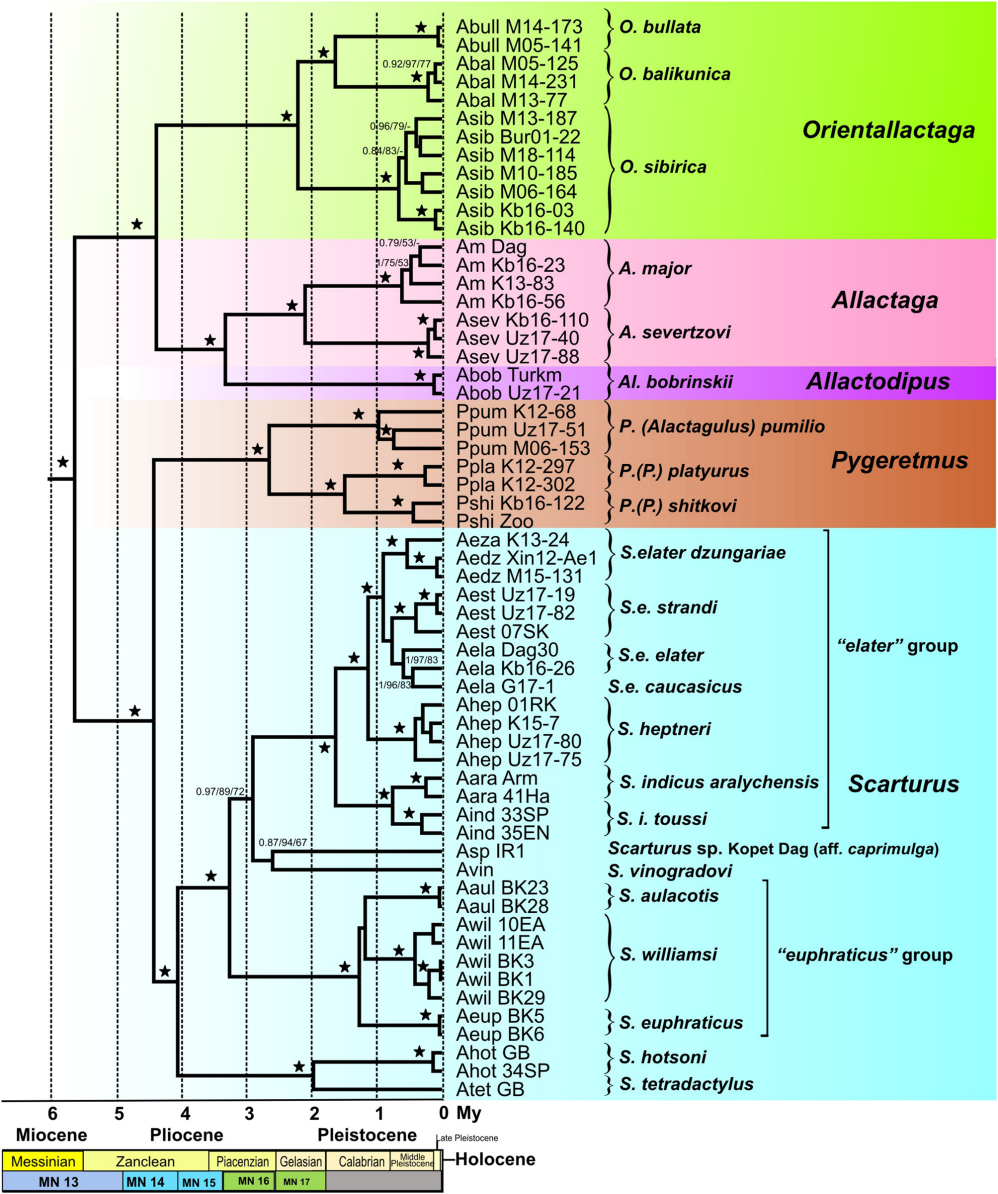
Chronogram of the divergence events in the Allactaginae produced by the BEAST algorithm based on the analysis of nuclear genes. Values above the branches correspond to posterior probabilities in Bayesian analysis (≥ 0.75) and bootstrap support (1000 pseudoreplicates, ≥ 50%) in the ML and MP analyses, respectively (BPP/ML/MP). Asterisks mark the nodes with absolute support in all analyses. Relationships among outgroup lineages are shown in Fig. S3.

Allactagines were split into two clades at the basal node (Fig. 2, Fig. S3): *Allactaga* + *Allactodipus* + *Orientallactaga* and *Scarturus* + *Pygeretmus*. Within the former clade, *Allactodipus* was consistently placed as a sister group to *Allactaga*. Monophyly of *Scarturus*, the genus *Pygeretmus*, and the subgenus *Pygeretmus* were robustly supported. The basal dichotomy within *Scarturus* separated the *S. tetradactylus* + *S. hotsoni* clade from a clade comprising four lineages: the *S. elater* species group, the *S. euphraticus* species group, *S. *sp*.* from Kopet Dag, and *S. vinogradovi* (designated VECE). Within this clade, the *S. euphraticus* species group occurred as a sister group to the other lineages, whereas *S. vinogradovi* appeared to form a clade with *Scarturus *sp*.* Kopet Dag*.*; however, both of these relationships were not robustly supported by all methods, which correlates with relatively short internode branches between consecutive divergences.

The relationships among *S. euphraticus*,* S. williamsi*, and *S. aulacotis* remained unresolved in all analyses, suggesting a hard polytomy. The *S. elater* species group included two lineages corresponding to *S. indicus* s.l. and *S. elater* + *S. heptneri*. *S. indicus* s.l. was split into two lineages: *S. indicus* proper and *S. indicus aralychensis. S. heptneri, S. elater dzungariae*, and *S. elater strandi* were placed as successive outgroups relative to *S. elater* sensu stricto (s.str.) including *S. e. caucasicus*.

All interspecific clades were found to be robust to gene removal with the exception of the relationship among the four main branches within the VECE clade, which appeared unresolved when the IRBP alignment was excluded.

The ML and Bayesian trees reconstructed from individual genes showed various degrees of resolution, branch support, and deviation from the concatenated nuclear tree. However, none of the gene trees contained any highly supported nodes (i.e. with ultrafast bootstrap support > 90% and posterior probability > 0.95) contradicting the concatenated topology.

### Mitochondrial results

**Figure 3:**
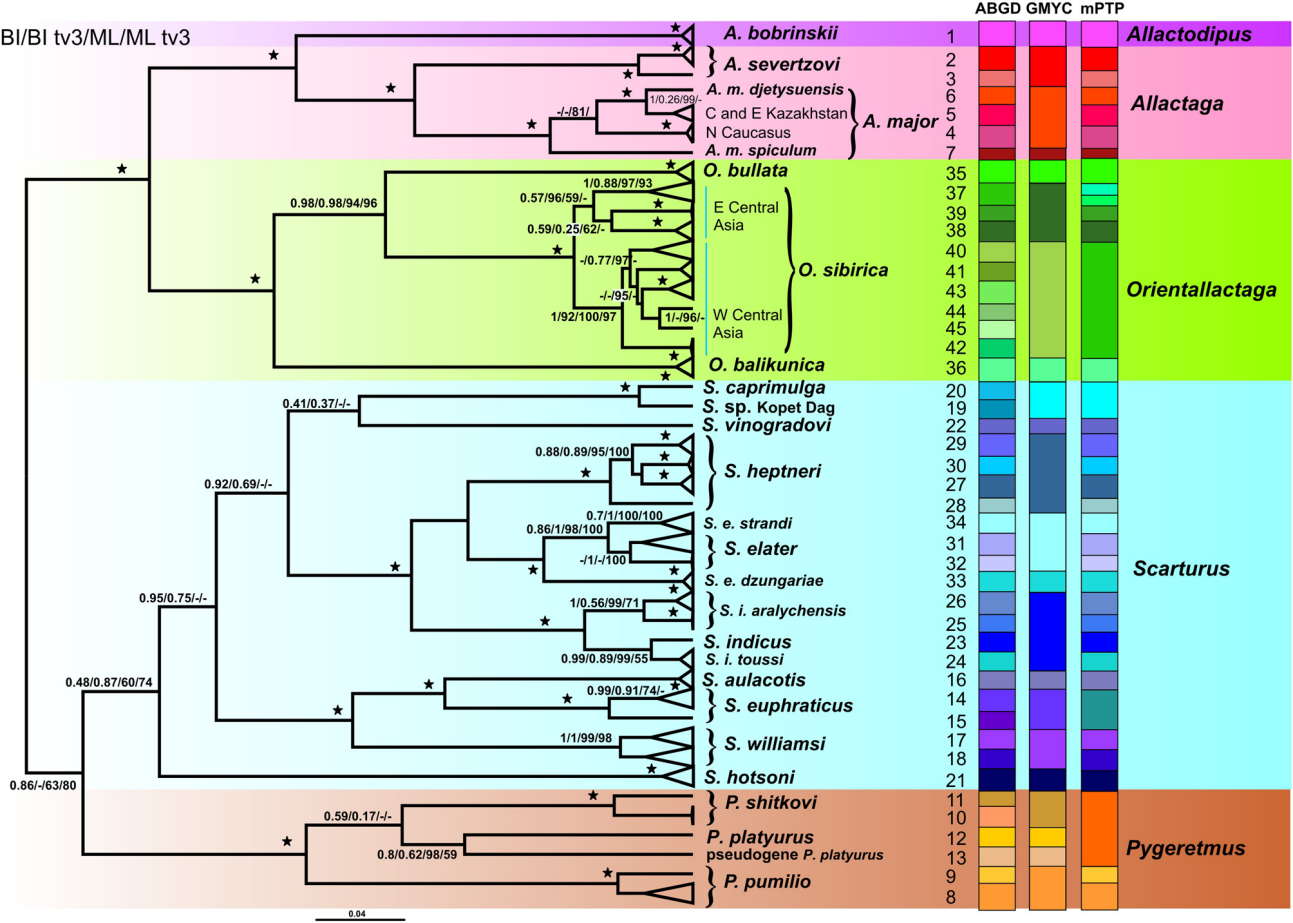
The phylogeny of Allactaginae produced by the BEAST algorithm based on the *cytb* gene sequences and the results of the ABGD, GMYC, and mPTP species delimitation analysis. Values above the branches correspond to posterior probabilities in BEAST and bootstrap support (1000 pseudoreplicates) in the ML analyses of all codon positions in *cytb* and 1st + 2nd + transversions of 3rd position, respectively (BPP/BPPtv3/ML/MLtv3).

Mitochondrial phylogenetic trees (Fig. 3, Fig. S4) were generally in agreement with the nuclear phylogeny. The support for the relationships among major lineages within the *Scarturus* + *Pygeretmus* clade was only low or moderate. The disagreements between nuclear and mitochondrial phylogenies were observed in the following: (1) *O. bullata* and *O. balikunica* were supported as sister taxa by the nuclear data, while in the mitochondrial tree, *O. bullata* formed a well-supported cluster with *O. sibirica*; (2) the clade comprising *S. vinogradovi* and *Scarturus *sp*.* Kopet Dag was not recovered in the mitochondrial ML analysis; (3) *S. heptneri* but not *S. indicus* was placed as the sister group to the rest of the *S. elater* species group in the mitochondrial ML tree; (4) the mtDNA data provided resolution for the relationships within the *S. euphraticus* species group where *S. euphraticus* formed a highly supported clade with *S. aulacotis*; (5) in the nuclear tree, *S. elater* from Zaisan clustered with *S. elater dzungariae*; however, the *cytb* data supported its proximity to *S. elater* s.str.; (6) *Euchoreutes* as the closest sister group of Allactaginae was not supported by the *cytb* data.

A few remarks should be made considering the phylogenetic position of taxa for which no nuclear data were available: (1) (*Scarturus *(*?williamsi*)* caprimulga* from Afghanistan was closely related to *Scarturus *sp*.* from Kopet Dag (*p* distance = 0.044); (2) *A. major spiculum* was placed as a divergent sister lineage to all other *A. major* specimens (*p* distance = 0.087); (3) as in Bannikova et al.^22^, *S. indicus* from Afghanistan clustered with specimens from East Iran described previously as *A. toussi*; (4) *O. sibirica* from the western part of the range (Kazakhstan, Uzbekistan), Tianshan and Dzungar Basin, was represented by six lineages with *p* distances of 0.037–0.049.

### Species delimitation

The subdivision pattern produced by ABGD corresponded to the barcode gap distance of 0.033. The sample was partitioned into 45 subsets (Fig. 3, Fig S4; for the description see Supplementary Information, ABGD results).

GMYC suggested 22 entities (confidence interval: 18–27; cut-off threshold ~ 0.05). The mPTP analysis supported recognition of 35 groups (Fig. 3). The results of the first two methods were generally consistent with the existing taxonomy. In contrast, the mPTP algorithm lumped two indisputable species, *P. shitkovi* (Kuznetsov, 1930) and *P. platyurus* (Lichtenstein, 1823), into a single cluster.

### Species tree reconstructions

Both *BEAST and ASTRAL produced species trees that were congruent with the topologies obtained from the nuclear concatenation (Supplementary Fig. S5). Most interspecific nodes were robustly supported both by posterior probabilities produced using *BEAST and consensus scores from ASTRAL.

### Divergence time estimation

The UCLD and RLC analyses in BEAST produced consistent results (Supplementary Table S9), thus indicating that, in this case, the choice of the clock model did not substantially affect the outcome. Linear regression of concatenation-based node ages against relative divergence times inferred by *BEAST indicated that the average interval between allele divergence and species divergence was close to 150 Ky, which can be used as a correction term to compensate for ancestral polymorphisms.

The time of the most recent common ancestor of living allactagines was estimated at 5.7–5.8 Mya (Messinian), which slightly predates the Miocene/Pliocene boundary. Intergeneric divergence times were dated to the Pliocene; however, the same was true for the oldest splits within *Scarturus*. Radiation within species groups (*S. elater* species group and *S. euphraticus* species group) was estimated to have started in the Early Pleistocene. Most taxa ranked as subspecies or closely related species were concluded to have diverged in the Middle Pleistocene.

Notably, node heights in the mitochondrial tree are not linearly related to the split ages estimated from nuclear concatenation (Supplementary Fig. S6), which is likely an effect of saturation of mtDNA sequences leading to an underestimation of the true level of divergence in more ancient nodes.

### Diversification analysis

In the phylogenetic tree produced from nuclear data, the MEDUSA algorithm identified a significant diversification increase along the branch leading to Allactaginae with the net diversification rate changing from 0.04 to 0.56. Within Allactaginae, no detectable rate shifts were found in either nuclear or mitochondrial trees. In both cases, the speciation pattern in allactagines was most accurately described by the Yule model.

### Biogeographic reconstruction

We performed the modelling of past distribution and produced the hypothetical speciation scenaria for all Pliocene–Pleistocene nodes. Scenaria for two cases that illustrate the vicariance and founder speciation models respectively are described below. The results of modelling for other 16 nodes are available in Supplementary Information (Biogeographic reconstructions, Fig. S7).

**Figure 4:**
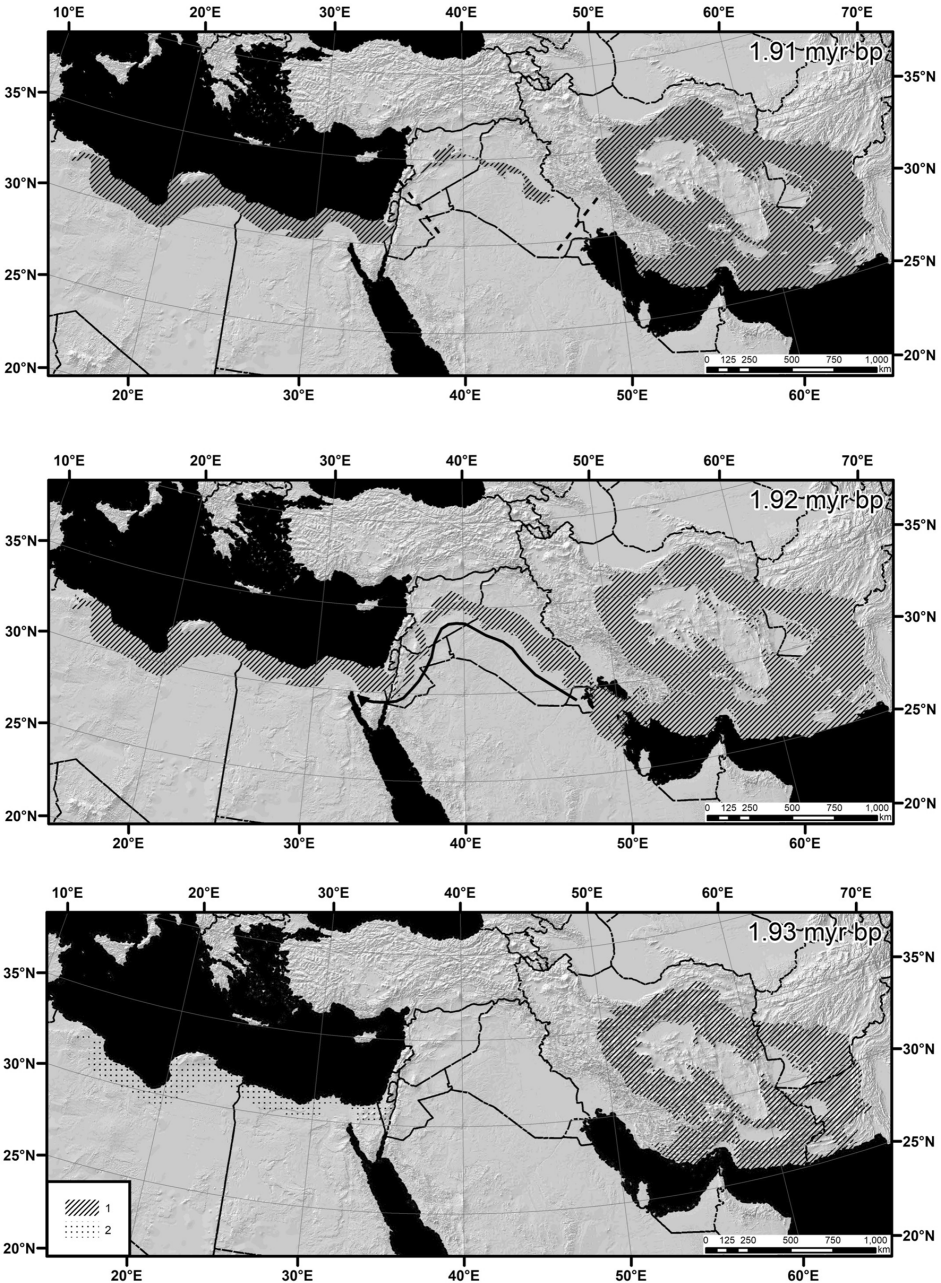
Geography of speciation in the node no. 12: divergence between *Scarturus hotsoni* and *S. tetradactylus.* Variables used in the modeling: Bio 1, Bio 4, Bio 7, Bio 7, Bio 9, Bio 15, Bio 16, and Bio 18. AUC = 0.963. Threshold value = 0.114. Stripes indicate expected true species range, whereas dots mark uninhabited isolated area with potentially suitable environment. The maps were obtained as raster maps from modeling using MAXENT 3.4.1 software and then generated as polygon maps using ArcGIS Desktop 10.8.1 software. MAXENT: available at https://biodiversityinformatics.amnh.org/open_source/maxent/. ArcGIS Desktop: Copyright © 1995–2020 Esri.

Node no. 12 (Fig. 4): divergence between *S. hotsoni* and *S. tetradactylus* 1.92–1.85 Mya (founder speciation model). Up to 1.93 Mya, the geographic range of the ancestral lineage was limited by central and eastern Iran, southern Afghanistan, and south-western Pakistan. A narrow corridor along southern and western foothills of Zagros Mountains, northern Mesopotamia, and Levant connected Iranian Highlands with North Africa 1.92 Mya. This corridor was closed from 1.91 Mya onwards. It seems obvious that the ancestor of *S. tetradactylus* dispersed into North Africa along this corridor, whereas the ancestor of *S. hotsoni* evolved in situ.

**Figure 5:**
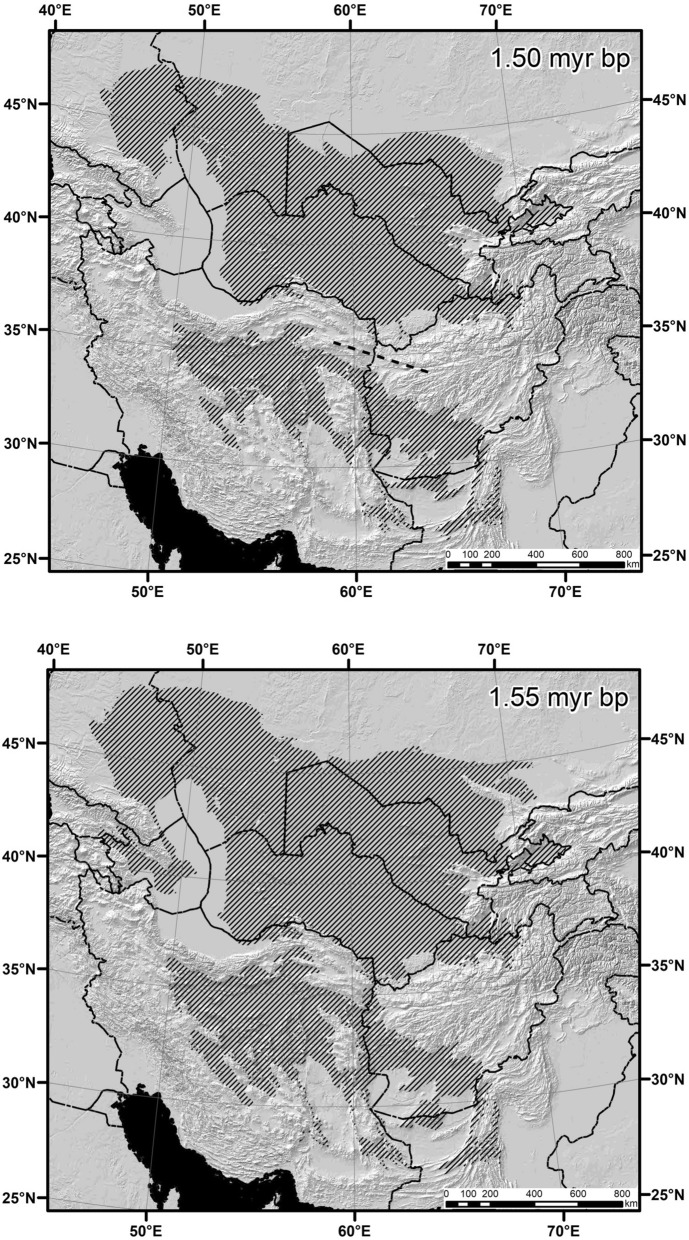
Geography of speciation in the node no. 13: divergence between *Scarturus elater* and *S. indicus* groups. Variables used in the modeling: Bio 3, Bio 7, Bio 9, Bio 10, Bio 11, Bio 12, and Bio 19. AUC = 0.875. Threshold value = 0.301. The maps were obtained as raster maps from modeling using MAXENT 3.4.1 software and then generated as polygon maps using ArcGIS Desktop 10.8.1 software. MAXENT: available at https://biodiversityinformatics.amnh.org/open_source/maxent/. ArcGIS Desktop: Copyright © 1995–2020 Esri.

Node no. 13 (Fig. 5): divergence between *S. elater* and *S. indicus* clades 1.58–1.50 Mya (vicariance model). The narrow corridor between eastern foothills of Kopet Dag and western foothills of Parapamiz mountains along the Tedzhen River valley connected northern and southern parts the *S. elater* groups’ geographic range until 1.55 Mya. This corridor was impenetrably closed 1.54 and 1.51–1.50 Mya and was re-opened after 1.49 Mya. It seems that due to isolation of northern and southern parts of the former geographic range, the ancestor of *S. elater* originated in Turan Plain, whereas the ancestor of *S. indicus* was formed in Iran Highland.

**Figure 6:**
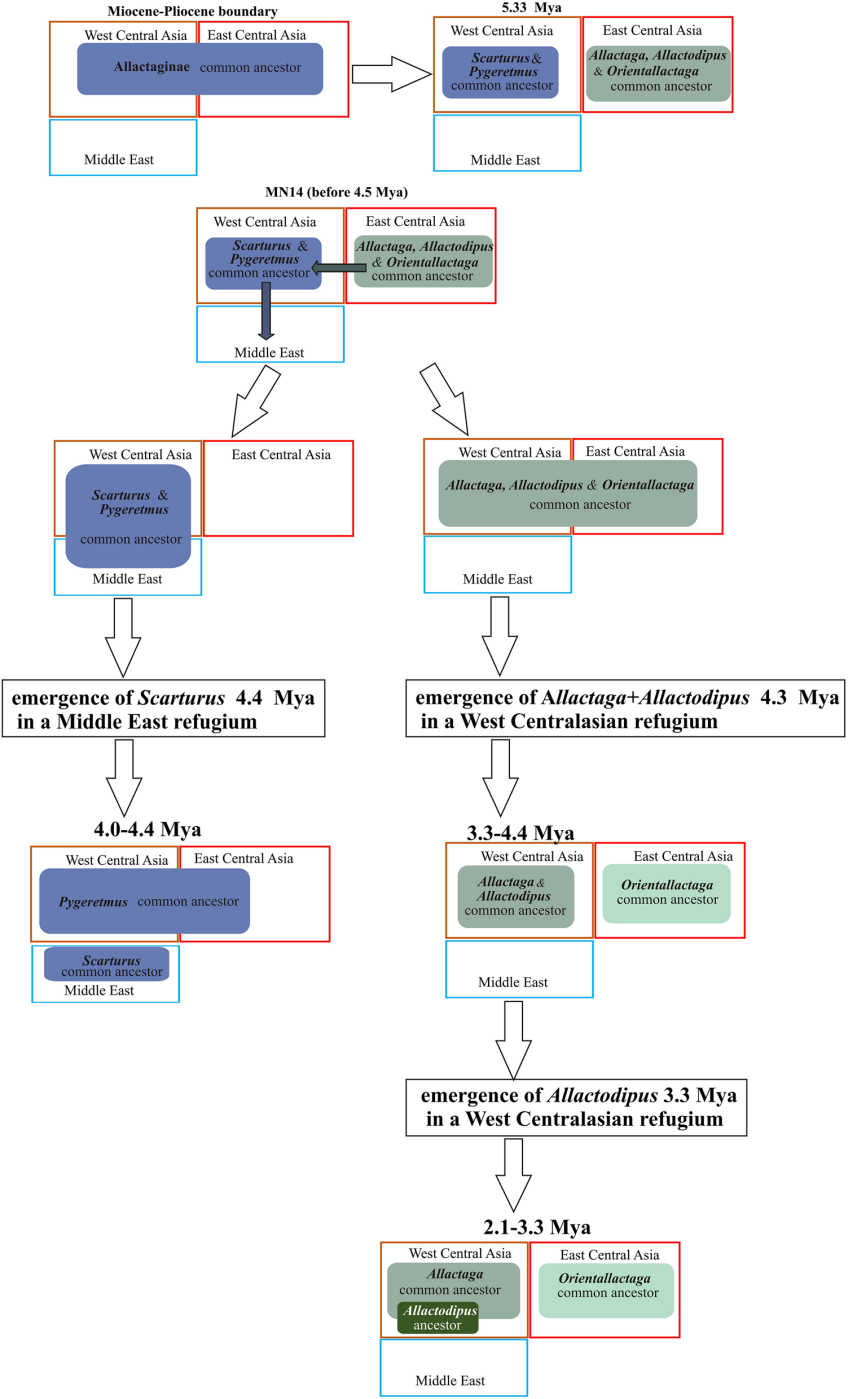
Hypothetical scenario of range evolution and geography of speciation during the early stages in phylogenetic history of Allactaginae. See text for explanations.

To summarize the results of the distribution modelling, we propose the following hypothetical scenario for the main stages of the range evolution at the genus level (Fig. 6). At the Miocene—Pliocene boundary, the distribution range of the common ancestor of crown allactagines covered the northern part of the Eurasian arid belt and included most of both East and West Central Asia but not the Middle East. During the wet period of the earliest Pliocene, the East and West Central Asia sectors of the range were separated by an environmental barrier and inhabited by the ancestors of the *Allactaga*-*Allactodipus-Orientallactaga* and *Scarturus-Pygeretmus* clades, respectively.

Two dispersal events occurred at the next stage (sometime before 4.3–4.5 Mya). The common ancestor of *Allactaga-Allactodipus-Orientallactaga* dispersed westward from East Central Asia into West Central Asia. At the same time, the ancestor of *Scarturus-Pygeretmus* dispersed southward, extending its range from West Central Asia into Iran and the Middle East.

The common ancestor of *Allactaga-Allactodipus* evolved in a West Central Asian isolate and all subsequent biogeographic events for this clade occurred in Turan. The speciation processes in *Orientallactaga* were mostly restricted to East Central Asia, but the origin of the *O. sibirica* lineage is associated with westward dispersal of its ancestor into West Central Asia at around 2.1 Mya (Fig. S7). The evolution of *Pygeretmus* was associated mainly with West Central Asian sector; speciation events took place in refugia in the eastern part of this area (Fig. S7). *Scarturus* originated in a Middle East isolate and later extensively radiated in SW Asia. This genus produced at least two northward colonization waves into West Central Asia that resulted in emergence of *S. elater* + *S. heptneri* (see above) and *S. vinogradovi*.

## Discussion

### Phylogenetic relations and systematics of Allactaginae

#### Intergeneric relations

Our data produced a robust phylogeny for Allactaginae above species level and thereby firmly proved that *Allactaga* s.l. (as recognised by Holden and Musser^17^) is paraphyletic to both *Pygeretmus* and *Allactodipus*. Both of the latter taxa are morphologically distinct from *Allactaga* by a number of unique apomorphies: a unique molar pattern and glans penis morphology in *Allactodipus* as well as high-crowned terraced molars, reduction of the premolar, and particular glans penis morphology in *Pygeretmus*. At the same time, the morphology of all other five-toed jerboas is relatively monotonous with variation only in terms of body size, relative molar crown height, size of auditory bullae, m1 morphotype frequency, and the rate of M^3^ reduction^1,45^. Such level of differences never allowed recognition of more than one genus.

Thus, allactagines represent a case when descendant lineages with derived morphology are nested within a group with overall conserved morphology. This can be compared to paraphyly of white-toothed shrews *Crocidura* relative to *Diplomesodon*^46^, rorquals (*Balaenoptera*) relative to humpback whales (*Megaptera*)^47^, or tits (*Parus* s.l.) relative to morphologically aberrant ground tit (*Pseudopodoces humilis*)^48^. In such cases, the taxonomy should be changed in accordance with the monophyly principle, which is achieved by combining genera (as done in whales) or splitting the genus in question into new taxa (as done in tits). Unfortunately, any decision in this context is arbitrary as it is based on subjective weighting of morphological differences. For Allactaginae, the splitting approach was implemented^18^, which resulted in the elevation of *Scarturus* and *Orientallactaga* to the generic rank^2^, despite the fact that a synapomorphy-based morphological diagnosis of *Scarturus* can hardly be formulated.

As an alternative to the morphology-based approach, temporal banding—a method which uses node age as a measure of rank^49^—was suggested as a standardised method for taxonomic ranking. In the present study, the age of divergence of major Allactaginae lineages was dated to the Pliocene. However, in other groups of Myodonta, Pliocene divergences were found both among genera (as in voles^50^ or hamsters^51^) and among congeneric species (as in *Sicista*^52^). Thus, the ambiguity remains unresolved; we see no better option than to retain the generic classification established by Michaux & Shenbrot^2^ (Table S10). However, it should be noted that the inferred age of divergence between *S. tetradactylus* + *S. hotsoni* and the VECE clades (3.9–4.1 Mya) is comparable or even larger than the divergence time of *Allactodipus* from *Allactaga*. If the temporal criterion (sensu Avise, Johns^49^) is accepted, one should consider elevating the VECE clade at least to subgeneric rank, with *Scarturus* proper including only two species. The diagnosis of the new taxon should be polythetic (medium to small jerboas with five-toes, bullae not enlarged, glans penis with longitudinal fold, molar low-to medium crowned, M3 not reduced). Although the name *Paralactaga* is traditionally used as a subgeneric for the *S. euphraticus* group and therefore may have been applied to the whole VECE clade, we believe that this is incorrect. The type species of *Paralactaga*—*P. anderssoni* Young, 1927—was described from the Late Miocene of China, which is inconsistent with the estimated time of origin of the VECE clade. Apparently all similarities between *S. euphraticus* group and *Paralactaga* proper are because of plesiomorphy. Therefore, we suggest that *Paralactaga* should be attributed to fossil taxa only.

#### Species groups within Scarturus

In the present study, we analysed in detail the phylogenetic reconstructions and divergence times estimations for the species and species groups of the genus *Scarturus*. Our study is the first to examine the phylogenetic position of the enigmatic taxon described from Afghanistan and which is currently termed *Scarturus williamsi caprimulga*. The mitochondrial data provided clear evidence that this taxon is not closely related to any member of the *S. euphraticus* species group including *S. williamsi*. Instead, it belongs to a separate divergent lineage of *Scarturus,* which should be considered a separate species, *Scarturus caprimulga*. It also includes the jerboa from Kopet Dag provisionally classified by Hamidi et al^25^ as *Paralactaga* cf. *williamsi*. The mitochondrial difference between specimens from Afghanistan and those from Kopet Dag suggested a potential subspecies rank of the latter form, which is provisionally referred to as *S. aff. caprimulga*. More research on the distribution and genetic structure of this species is needed for further clarification. Our study has added more representative genetic data on the poorly known *S. vinogradovi* and confirmed it as a separate divergent branch within *Scarturus* s.l. and likely a distant sister group of *S. caprimulga*.

Previous phylogenetic reconstructions of the *S. euphraticus* species group based on mtDNA data recovered a divergent branch within *S. euphraticus*^53^, which was subsequently classified as *S. aulacotis*^2^. With further addition of comprehensive nuclear data, the full species rank of this taxon is now completely supported. The relationships among the three species in the *S. euphraticus* group correspond to a hard trichotomy dated to the late Early Pleistocene.

Nuclear data strongly support deep structuring within the *S. elater* species group, as previously demonstrated using mtDNA^19,22,54^, and confirmed the species status of *S. indicus* and *S. heptneri*. The divergence between *S. elater* and *S. indicus* estimated based on the nuclear loci was dated to approximately 1.5 Mya, which was slightly older than the 1.26 Mya inferred from mtDNA by Bannikova et al.^22^. Both *S. indicus* and *S. elater* included allopatric lineages that have separated 600–800 kya (i.e. *dzungariae* and *strandi* within *elater*, and *aralychensis* within *indicus*). Their formal taxonomic rank appears controversial: the level of divergence apparently conforms to species rank, whereas genetic data indicates potential gene flow between them. Thus, the mtDNA haplotypes of *Scarturus* specimens from the Zaisan depression (*S. e. zaisanicus*) form a subclade within *S. elater* s.str., whereas nuclear data suggest that *S. e. zaisanicus* is relatively close to *S. e. dzungariae*. This pattern suggests that the Zaisan population, while being a derivative of the Dzungar form, experienced mtDNA capture as a result of a past hybridisation event with *S. elater*. Gene flow between *S. strandi* and *S. elater* proper was indicated by the occurrence of *elater* mtDNA haplotypes in certain populations of *strandi* from north-western Kyzylkum^22^. All these taxa require additional research to produce a more accurate evaluation of gene flow intensity. Nevertheless, we suggest that *dzungariae*, *strandi*, and *aralychensis* should be considered semispecies or species in statu nascendi. Taxonomically, we regard them as parts of *elater* and *indicus* superspecies and refer to them as *S. *(*elater*)* dzungariae*, *S. *(*elater*)* strandi*, and *S. *(*indicus*)* aralychensis*, respectively.

#### Phylogenetic relations within Orientallactaga

Within *Orientallactaga*, *O. bullata* and *O. balikunica* were supported as sister taxa based on nuclear data, which is consistent with their common morphology (enlarged bullae). However, mtDNA suggested that *O. bullata* is a sister taxon to *O. sibirica*, and the reason for this discrepancy is unclear, with ancient mtDNA introgression being the most obvious explanation. The crown age of *Orientallactaga* was dated to the early Early Pleistocene (Gelasian). Neither *O. bullata* nor *O. balikunica* show substantial intraspecific variation.

In contrast, *O. sibirica* consists of several genetic lineages, which partly correspond to recognised subspecies. The mtDNA data tentatively supported subdivision of *O. sibirica* into western and eastern groups separated by the Tianshan–Altay zoogeographic boundary. The structure of variation in the eastern portion of the range (Mongolia, China) is well-studied^23^; however, the genetic data on the western portion are still fragmentary. Available mtDNA data provisionally support recognition of western subspecies such as *O. s. ognevi* (north-eastern to central Kazakhstan), *O. s. dementjevi* (Issyk-Kul region), and *O. s. altorum* (central Tianshan). The latter two forms are distributed in high-altitude areas of Tianshan, thus indicating that, in contrast to most other jerboa species, mountain areas might serve as foci of diversification in *O. sibirica*.

The westernmost part of the range (western Kazakhstan, Qyzylkum) was assumed to be inhabited by a single *O. s. suschkini* subspecies after morphological revision^1^. However, three divergent mtDNA lineages were recovered based on the preliminary analysis of mtDNA data retrieved from museum specimens from the area, which suggests that the diversity of western populations is likely underestimated and in need of further examination.

The crown age of *O. sibirica* was estimated at 500–600 kya, which was substantially younger than 2.2–3.2 Mya as inferred by Cheng et al.^23^; this discrepancy, however, can be explained by mtDNA saturation effects and usage of inaccurate secondary calibrations in their study.

#### Variation within Allactaga and Pygeretmus

Considering the phylogenetic position of *Pygeretmus*, our data firmly corroborated its separate phylogenetic position and rejected any affinity with *Orientallactaga bullata* as reconstructed by Wu et al.^55^. The latter result should be attributed to identification error. In our study, all three species of *Pygeretmus* were analysed to confirm phylogenetic proximity of *P. shitkovi* and *P. platyurus* relative to *P. pumilio*. Thus, the subgeneric status of *Alactagulus* containing the latter species was not contradicted; however, the split age between *Pygeretmus* s.str. and *Alactagulus* is relatively young, dated as Pliocene/Pleistocene boundary, indicating that morphological and life history traits of the former (e.g. slower locomotion) have evolved rather recently.

A further taxon demonstrating a complex structure is *Allactaga major*. Our mtDNA data indicated that *A. major* consisted of several genetic lineages partly corresponding to morphological subspecies (*A. m. spiculum*, *A. m. djetysuensis*). A high level of divergence was observed between specimens from the northern Caucasus and Kazakhstan. A specimen of morphologically distinct *A. m. spiculum* (north-eastern Kazakhstan, western Siberia) was placed as a sister species to all other *A. major* with a divergence level compatible with species status.

Several other species included unexpected genetic lineages that were apparently divergent at subspecies level (e.g. a southern Uzbekistan lineage of *A. severtzovi* and an Ili lineage of *P. shitkovi*). However, the resolving power of the employed set of 15 nuclear genes is insufficient for clarifying relationships within species. Therefore, these cases should be studied using larger samples and further nuclear loci.

### Divergence time estimates within Allactaginae

Our estimated divergence times were generally more recent than those produced by most previous studies. The root node of crown Allactaginae was dated to 7.7 (5.4–9.9) Mya by Wu et al.^55^, 8.1 (4.2–12.7) Mya by Zhang et al.^56^, or 8.87 (8.3–9.85) Mya by Pisano et al.^4^. The results by Wu et al.^55^ may be affected by a node density effect as their re-analysis with reduced taxon sampling of Allactaginae and Dipodinae produced younger dating at 5.8 (3.1–8.6) Mya. The latter two studies used only one to four nuclear loci and calibrated their analysis using non-Dipodidae calibration points. In both cases, the Early Miocene age of *Sicista primus* was used to calibrate crown *Sicista*, which lacks proper justification and may result in upward bias, as argued by Rusin et al.^57^.

The earliest Allactaginae appeared in the Early Miocene and, in the Middle Miocene, the members of the primitive genus *Protalactaga* Young, 1927 became a common element of the Asian fauna^3^. During the Late Miocene, the diversity of allactagines persisted, and new genera emerged including *Paralactaga* Young, 1927 which is morphologically similar to *Allactaga* and is often considered its subgenus^3,45^. However, as can be derived from our results, all but one of the Middle and Late Miocene lineages went extinct without leaving any recent descendants, and all current diversity is a product of the Pliocene–Pleistocene evolution. This diversification pattern is unlike that observed in a different jerboa subfamily, Dipodinae, which includes lineages that had diverged in the Middle and early Late Miocene (*Paradipus* and *Dipus*, respectively)^4,58^.

As estimated here, the onset of radiation among crown Allactaginae occurred in the latest Messinian and thus was nearly coincident with the Messinian crisis. However, it remains unclear how (or whether at all) climatic perturbations at the Miocene /Pliocene boundary affected the evolution of Allactaginae. The results of the diversification analysis suggested that, throughout the Pliocene and Pleistocene, the rate and mode of speciation in five-toed jerboas remained constant, indicating high tolerance of this group towards the climatic changes of this period.

The minimum age of split observed between sympatric species was approximately 1 Mya as demonstrated by *heptneri* versus *elater* s.str. (and *strandi*). This was the estimate for the minimum time necessary for formation of effective reproductive barriers in allactagines (post- or pre-zygotic). Other phylogenetically close sympatric species pairs were S. *elater*/*S. indicus* (1.5 My), *O. bullata*/*O. balikunica* (1.5 My), and *A. major*/*A. severtzovi* (2.0 My).

### Geography of speciation

Of 17 analysed episodes of speciation in Pliocene–Pleistocene, the patterns of range fragmentation in 10 episodes matched well to the classical vicariance scenario and those of six episodes matched to the founder-event speciation scenario; in one episode, both scenarios were equally probable. As the location of arising isolation barriers within the ancestor range seemed incidental, only in three cases the ancestors’ range was subdivided into two parts which were more or less equal in size: first, into East and West Central Asia; second, into Turan and Iran; third, into Anatolia with trans-Caucasus and northern Zagros and Levant with northern Mesopotamia and southern Zagros. In all other cases, the ancestors’ range was subdivided into the main part and relatively small peripheral isolates. As can be expected from the modern patterns of species diversity of Allactaginae, the discovered speciation events were unequally distributed: one episode in North Africa, one in the eastern part of Central Asia, three in the Middle East, four in the Iranian highland, four in Turan, and five in Kazakhstan. In most cases, range fragmentation coincided with extreme climate conditions within the analysed time periods: warmest and wettest (decrease of the area of arid lands: nodes 2–3, 5, 10, 12, and 14–15) or coldest and driest (closing narrow mountain passages due to mountain glaciation: nodes 4, 6–9, 13, and 16–18). In one case (node 11), fragmentation of the range coincided with moderate climate conditions.

Successful modelling of fragmentation of geographic ranges as a base of speciation events seemed to agree with the hypothesis of Peterson et al.^15^, which states that ecological niches evolve little at or around the time of speciation events, whereas niche differences accumulate later. This hypothesis was supported by Peterson’s analysis^59^ of data published between 1999 and 2008 which demonstrated that niche conservatism was found in more than 70% of comparisons within species and between sister species, but in less than 50% of comparisons among closely-related (but not sister) species and across monophyletic lineages of species. Moreover, analysis of habitat niche evolution of arvicoline rodents^16^ demonstrated that closely related species with allopatric or parapatric distribution demonstrated small niche differences, whereas they were larger in species with sympatric distribution. This is a clear indication that interspecific competition forces natural selection to increase niche differences resulting in species co-occurrence. It was demonstrated that niche divergence/conservatism can be differently expressed between different niche/resource axes^60^. In voles, which have a highly specialised folivorous diet, habitat segregation seems to be the only type of niche differentiation. Closely related Allactaginae species are similar in diet and typically occur in allopatric or parapatric distribution patterns^1^, which may indicate their niche conservatism. The only exception to a pattern where species with similar diets show widely overlapping geographic distributions are *Scatrurus elater* and *S. heptneri* (these two species are similar in both, macro- and micro-habitat niches, and it is unclear which mechanisms allow them to co-occur^22^). Distantly related sympatric species typically show similarities regarding macro-habitat niches but marked differences in terms of micro-habitat niches (*Allactaga major* and *Orientallactaga sibirica*; *O. sibirica* and *O. bullata*; *O. sibirica* and *O. balikunica*; *Pygerethmus pumilio* and *P. platyurus*; *P. pumilio* and *P. shitkovi*; personal observations) and diet (*Allactaga* and *Allactodipus*; *Allactaga* and *Scarturus*; *Allactaga* and *Pygeretmus*; *Orientallactaga* and *Pygeretmus*; *Scarturus* and *Pygeretmus*^1,61^). Thus, macro-habitat niche conservatism may be expected even in sympatric species.

## Supplementary Information

Supplementary Information.
