# Supplementary material for: Phylogenetic relations and range history of jerboas of the Allactaginae subfamily (Dipodidae, Rodentia)
Source: Sci Rep. 2022 Jan 17;12:842. doi: 10.1038/s41598-022-04779-x (PMC8764116; doi:10.1038/s41598-022-04779-x)
Supplement: Supplementary file 1 — Supplementary Information. [file 41598_2022_4779_MOESM1_ESM.pdf]

Supplementary Information

Phylogenetic relations and range history of jerboas of the Allactaginae subfamily (Dipodidae, Rodentia)

Vladimir S. Lebedev,
Georgy I. Shenbrot,
Boris Krystufek,
Ahmad Mahmoudi,
Marina N. Melnikova,
Evgeniya N. Solovyeva,
Alexandra A. Lisenkova,
Enkhbat Undrakhbayar,
Konstantin A. Rogovin,
Alexey V. Surov,
Anna A. Bannikova

Figure S1. Distribution ranges of the recent species of Allactaginae as inferred from the occurrence records in MAXENT.

A) *Allactaga* and *Allactodipus*: 1, *Allactaga major* with subspecies *A. m. major* (**maj**), *A.m. spiculum* (**spi**), and A. *m. djetysuensis* (**dje**); 2, *Allactaga severtzovi*; 3, *Allactodipus bobrinskii*; 4, zone of sympatry between *A. major* and *A. severtzovi*; 5, zone of sympatry between *A. bobrinskii* and *A. severtzovi*.

B) *Orientallactaga*: 1, *O. sibiric*a with subspecies *O. s. sibirica* (**sib**), *O. s. ognevi* (**ogn**), *O. s. suschkini* (**sus**), *O. s. altorum* (**alt**), *O. s. dementiewi* (**dem**); 2, *O. balikunica*; 3, *O. bullata*; 4, zone of sympatry between *O. sibirica* and *O. bullata*; 5, zone of sympatry between *O. sibirica* and *O. balikunica*; 6, zone of sympatry between *O. sibirica*, *O. bullata*, and *O. balikunica*.

C) *Pygeretmus*: 1, *P. pumilio*; 2, *P. platyurus*; 3, *P. shitkovi*; 4, zone of sympatry between *P. pumilio* and *P. platyurus*; 5, zone of sympatry between *P. pumilio* and *P. shitkovi*; 6, zone of sympatry between *P. pumilio*, *P. platyurus*, and *P. shitkovi.*

D) *Scarturus elater* species group: 1, *S. elater* with subspecies *S. e. elater* (***ela***), *S. e. strandi* (***str***), *S. e. dzungariae* (***dzu***), *S. e. zaisanicus* (***zai***), and *S. e. caucasicus* (***cau***); 2, *S. heptneri*; 3, *S. indicus* with subspecies *S. i. indicus* (***ind***), *S. i. toussi* (***tou***), and *S. i. aralychensis* (***ara***); 4, zone of sympatry between *S. elater* and *S. heptneri*; 5, zone of sympatry between *S. indicus*, *S. elater*, and *S. heptneri*.

E) *Scarturus* (other than the *S. elater* species group): 1, *S. tetradactylus*; 2, *S. hotsoni*; 3, *S. vinogradovi*; 4, *S. caprimulga*; 5, *S. aulacotis*; 6, *S. euphraticus*; 7, *S. williamsi*.

All maps were obtained as raster maps from modeling using MAXENT 3.4.1 software and then generated as polygon maps using ArcGIS Desktop 10.8.1 software. MAXENT: available at https://biodiversityinformatics.amnh.org/open_source/maxent/ ArcGIS Desktop: Copyright © 1995-2020 Esri

A

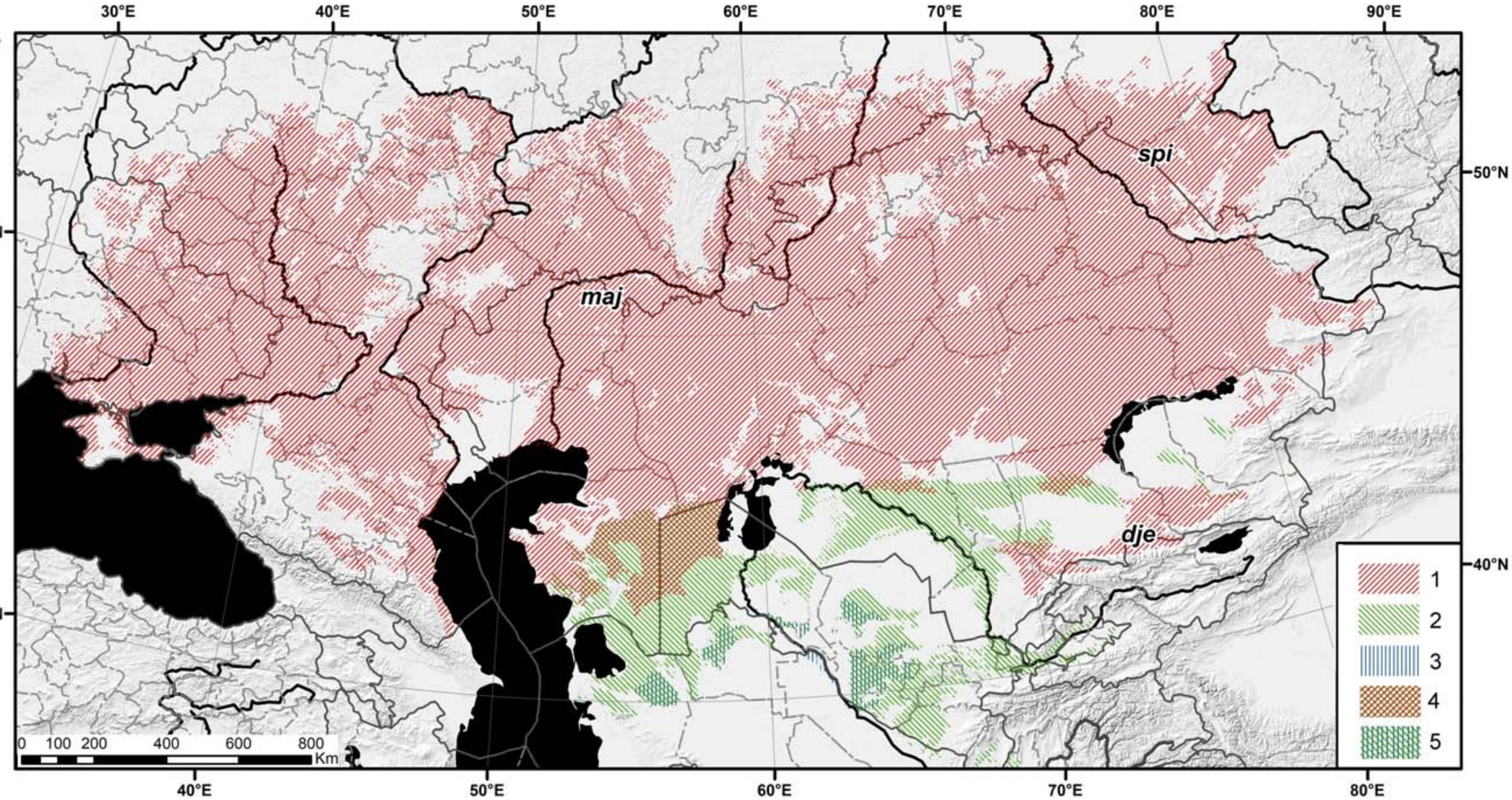

30°E
40°E
50°E
60°E
70°E
80°E
90°E
50°N
40°N
spi
maj
dje
1
2
3
4
5
0 100 200 400 600 800 Km

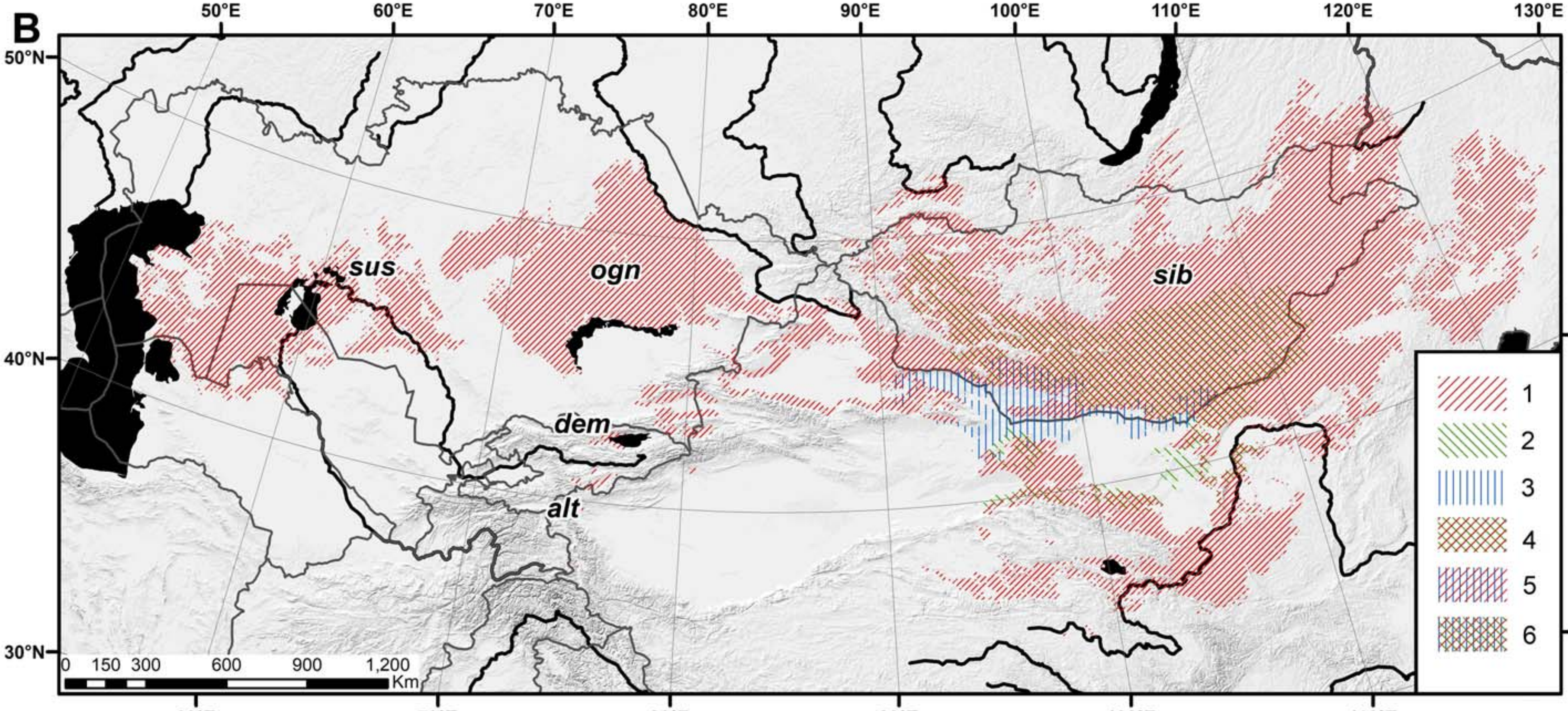

B
50°E
60°E
70°E
80°E
90°E
100°E
110°E
120°E
130°E
50°N
40°N
30°N
60°E
70°E
80°E
90°E
100°E
110°E
40°N
30°N
sus
ogn
sib
dem
alt
1
2
3
4
5
6
0 150 300 600 900 1,200
Km

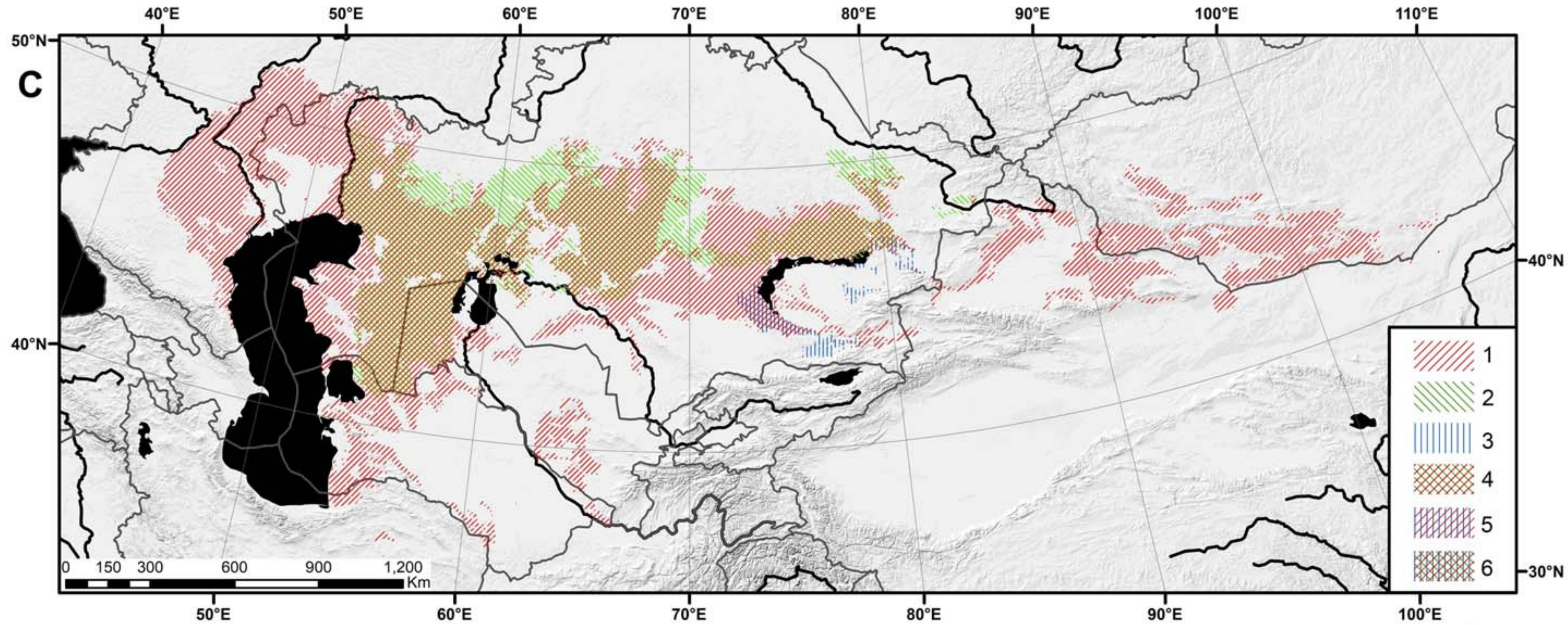
C
40°E
50°E
60°E
70°E
80°E
90°E
100°E
110°E
50°N
40°N
30°N
0 150 300 600 900 1,200
Km
1
2
3
4
5
6

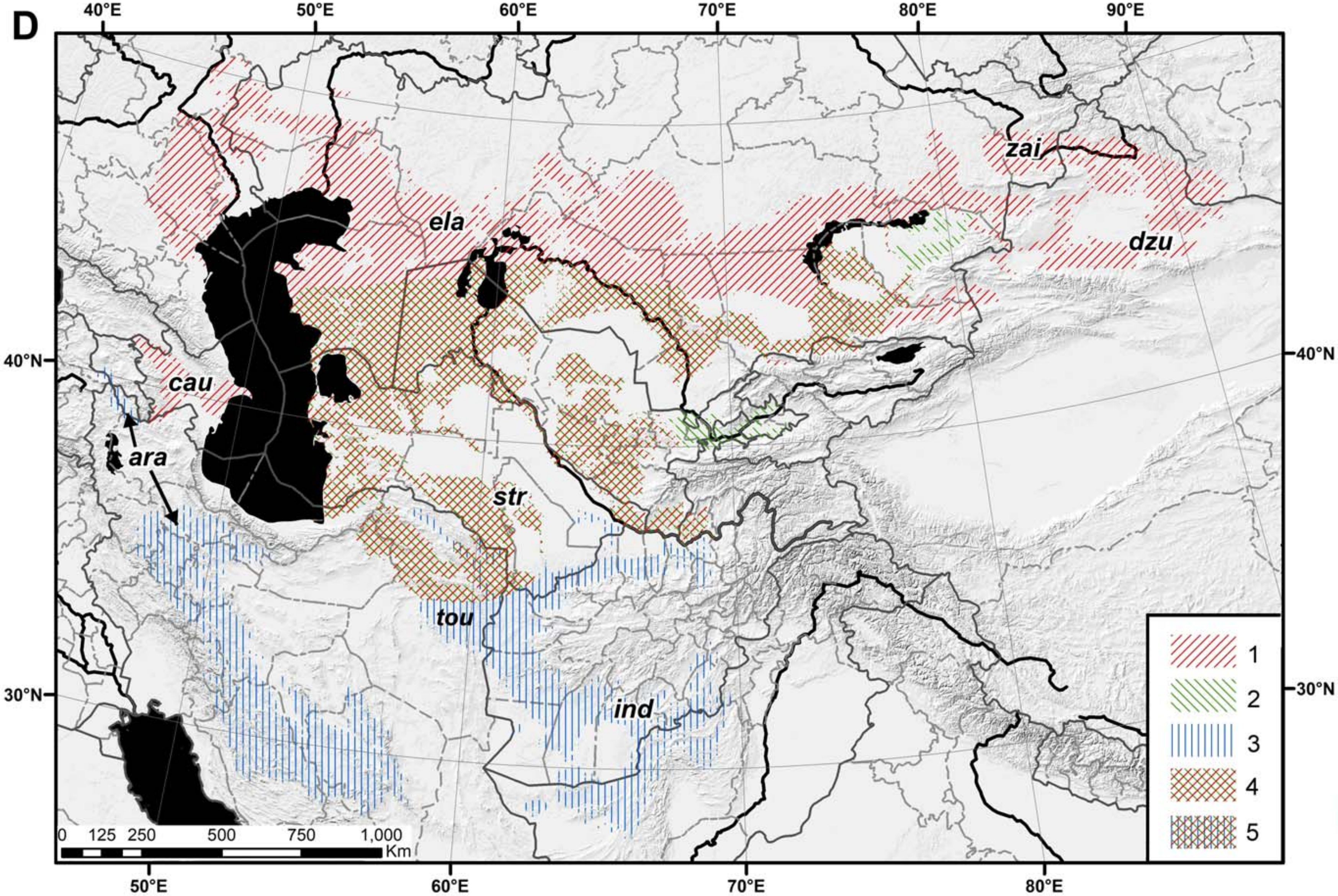
D
40°E
50°E
60°E
70°E
80°E
90°E
40°N
30°N
zai
ela
dzu
cau
ara
str
tou
ind
1
2
3
4
5
0 125 250 500 750 1,000
Km

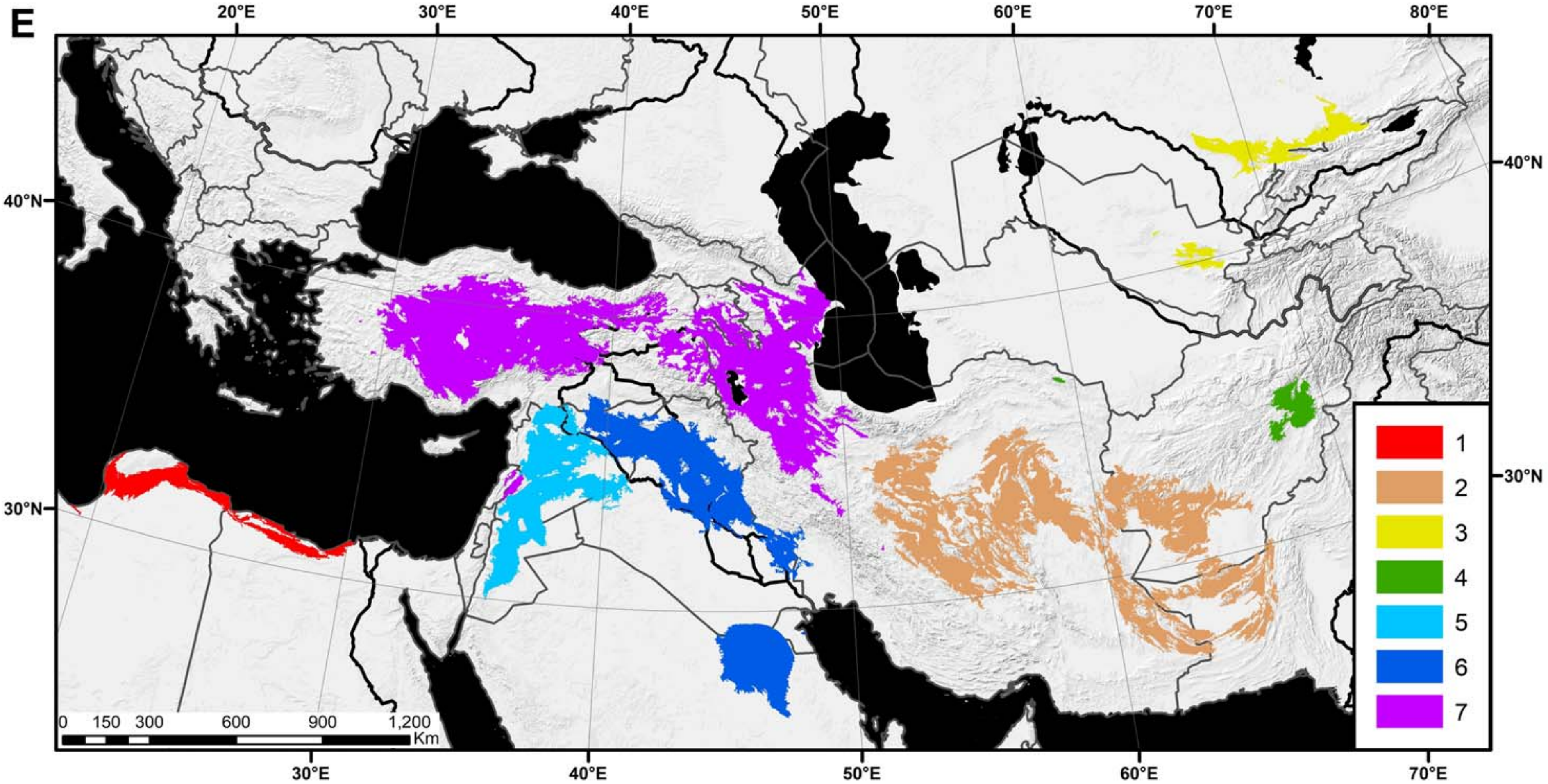
E
20°E
30°E
40°E
50°E
60°E
70°E
80°E
40°N
30°N
1
2
3
4
5
6
7
0 150 300 600 900 1,200
Km

Table S1. Characterization of the original material used in the study.

Species	specimen code (Fig. 1–2, S1–S4 ref.)	museum catalog number or tissue code	Collecting locality	Latitude	Longitude
Allactaga major	Am Dag	ZMMU 01/08_32	Russia, Dagestan, Chakanny	43.792°	47.522°
	Am 4	ZMMU S196092	Moscow Zoo		
	Am K12-263	Turgay K12-263	Kazakstan, Kara-Turgay River valley, Urpek-Karasu Rd.	50.175°	65.393°
	Am K12-274	Turgay K12-274			
	Am K12-295	Turgay K12-295			
	Am K13-83	ZMMU S192601	Kazakhstan, East Kazakhstan reg., Shakelmes, N shore of Zaisan lake	48.073°	84.130°
	Am Kb16-23	ZMMU S197282	Kazakhstan, Qaraghandy Reg., NW shore of Lake Balkhash, 30 km SW from Gulshad vill.	46.385°	74.017°
	Am Kb16-56	ZMMU S197283	Kazakhstan, Jambyl Reg.,NE slope of Chuili Mts.	44.438°	73.986°
	Am Kb16-141	ZMMU S197284	Kazakhstan, East Kazakhstan reg., 3.5 km NE from Baykoshkar vill.	48.407°	78.810°
	Am spiculum 143270*	ZMMU S143270	Kazakhstan, East Kazakhstan reg., Belagash (1987)	50.787°	80.866°
Allactaga severtzovi	Asev Kb16-110	ZMMU S197285	Kazakhstan, Almaty Region, left bank of the Karatal river, 10 km W of Naimansuek vill.	45.693°	77.221°

	Asev Uz17-40	ZMMU S198824	Uzbekistan, Karakalpakstan, 10 km SE from Bozgul Hill	42.989°	61.319°
	Asev Uz17-88	ZMMU S198826	Uzbekistan, Samarqand Reg., W of Zaravshan Mts., 3 km NE Jam vill.	39.453°	66.439°
	Asev 2006	uncatalogized (presented by A.V.Abramov)	Uzbekistan, C Kyzylkum, Dzharakuduk vill., 40 km SW Mynbulak	42.115°	62.629°
Allactodipus bobrinskii	Abob Turkm	uncatalogized (presented by D.A.Kramerov)	Turkmenistan, Chardzhou region, left bank of Amur-Daryi, 6 km W Lebap vill.		
	Abob Uz17-21	ZMMU S198828	Uzbekistan, Navoi region, 23 km WNW from Uchkuduk	42.209°	63.284°
	Abob Uz17-22	ZMMU S198829			
Orientallactaga sibirica	Asib Bur01-22	ZIN 86161	Russia, Buriatia, Kyakhtinsky district, 4-5 km N of Kiran vill.	50.467°	106.817°
	Asib M18-114	M18-114	Mongolia, Erdenet Tsaagan Nuur, 20 km S of somon	45.914°	115.368°
	Asib Kb16-03	ZMMU S197287	Kazakhstan, Qaragandy reg. Bektau Ata, N slope	47.481°	74.695°
	Asib Kb16-140	ZMMU S197291	Kazakhstan, E Kazakhstan reg., 4 km from Baikoshkar	48.407°	78.810°
	Asib M10-185	ZMMU S187318	Mongolia, Ubs aymag, Achit Nuur SE shore	49.478°	90.631°
	Asib M13-187	ZMMU S192231	Mongolia, Ömnögovi aymag, S Borzongiin Gobi	42.232°	105.413°
	Asib M06-164	ZMMU	Mongolia, Khovd	47.567°	93.469°

		S181016	aymag, S shore Durgen-nur Lake		
	Asib M12-68	ZMMU S190966	Mongolia, Govi-Altai aymag, Alag-Nuur	45.191°	94.593°
	Asib M14-64	ZMMU S193952	Mongolia, Sukhbaatar aymag, E of Darganga	45.217°	114.168°
	Asib M14-86	ZMMU S193953	Mongolia, Sukhbaatar aymag, to the W of Ongon	45.385°	112.503°
	Asib M14-144	ZMMU S193956	Mongolia, Dorongovi aymag, NW of Saralyn-nuur	42.751°	109.733°
	Asib M14-171	ZMMU S193959	Mongolia, Dorongovi aymag, west of Tenger-toirom	42.653°	108.302°
	Asib M13-216	ZMMU S192233	Mongolia, Ömnögovi aymag, Tavan-Aldyn Khuduk	42.411°	106.116°
	Asib Xin12-As1	As1	China, Xinjiang, Barkol, 25 km NW	43.753°	92.784°
	Asib M11-99	ZMMU S188951	Mongolia, Govi-Altai aymag, Gun-Tamga-Bulag	45.259°	93.654°
	Asib EK	ZMMU S182674	Kazakhstan, E Kazakhstan reg., Teristanbaly	50.069°	80.762°
	Asib 146874*	ZMMU S146874	Kazakhstan, Almaty Reg., 15 km NW Druzhba (1988)	45.311°	82.307°
	Asib 73068*	ZMMU S73068	Kyrgyzstan, Issyk-Kul Reg., Kurskoye (1955)	42.618°	76.965°
	Asib 69280*	ZMMU S69280	Kazakhstan, Mangystau Reg., N Usturt, Karatyuley Sor (1958)	44.804°	55.672°
	Asib 145985*	ZMMU S145985	Kazakhstan, Qyzylorda	46.797°	61.616°

			Reg., Aralsk (1988)		
	Asib 73076*	ZMMU S73076	Kyrgyzstan, Naryn Reg., Atbashi (1957)	41.188°	75.868°
	Asib 73081*	ZMMU S73081	Kyrgyzstan, Naryn Reg., Aksai (1957)	40.877°	76.188°
	Asib 146877*	ZMMU S146877	Kazakhstan, Almaty Reg, Dzharkent distr., Tyshkan (1988)	44.320°	80.084°
	Asib 143272*	ZMMU S143272	Kazakhstan, Qyzylorda Reg., N Qyzylqum, 8 km N Irisbay (1987)	43.981°	63.323°
	Asib 57308*	ZMMU S57308	Kyrgyzstan, Issyk-Kul Reg., Akulen (1954)	42.353°	76.180°
O. bullata	Abull M14-173	ZMMU S193945	Mongolia, Dorongov aymag, Tenger ToiromW	42.654°	108.302°
	Abull M05-141	ZMMU S179572	Mongolia, Dundgovi aymag, Luus-somon 10 km W	45.514°	105.628°
	Abull M12-97	ZMMU S190960	Mongolia, Govi-Altai aymag, Haryn-Shang	44.504°	95.622°
	Abull M12-98	ZMMU S190961			
	Abull M13-182	ZMMU 44/13	Mongolia, Ömnögovi aymag, NE from Hurmen	43.367°	104.301°
	Abull EG	ZMMU S188089	Mongolia, Ömnögovi aymag, Manlai–Mandakh road	44.277°	107.375°
O. balikunica	Abal M05-125	Abal	Mongolia, Bayankhongor aymag, Ekhiin-gol	43.251°	98.993°
	Abal M13-77	ZMMU S192212	Mongolia, GoviAltai aymag, Dzamyn-Bilgekh-bulag	43.099°	97.488°
	Abal M14-231	ZMMU	Mongolia, Ömnögovi	42.483°	105.254°

		S193939	aymag, Borzongiin Gobi, Gashuun-bulag		
	Abal M13-147	ZMMU S192214	Mongolia, Ömnögovi aymag, Dzulganai	43.583°	100.066°
	Abal M11-226	ZMMU S188941	Mongolia, Govi-Altai aymag, Altai-somon, 15 km WNW	44.649°	94.746°
	Xin12-Ab1	Ab1	China, Xinjiang, Mori, Hunlyushu	44.560°	91.341°
Pygeretmus (Alactagulus) pumilio	Ppum K12-68	ZMMU S190928	Kazakhstan, East Kazakhstan Reg., Shakelmies	48.070°	84.131°
	Ppum Uz17-51	ZMMU S198859	Uzbekistan, Republic of Karakalpakstan, Delta of the Ahchadarya dry river	42.913°	61.332°
	Ppum Uz17-54	ZMMU S198862			
	Ppum M06-153	ZMMU S181007	Mongolia, GoviAltai aymag, W shore of the Beger-Nuur lake	45.883°	96.880°
	Ppum M11-100	ZMMU S189185	Mongolia, GoviAltai aymag, Gun-Tamga-Bulag	45.259°	93.654°
	Ppum K12-245	Turgay K12-245	Kazakstan, Kara-Turgay River valley, Urpek-Karasu Rd.	50.175°	65.393°
	Ppum K12-251	Turgay K12-251			
	Ppum K12-261	Turgay K12-261			
	Ppum K12-262	Turgay K12-262			
	Ppum K12-204	Turgay K12-204			
	Ppum Xin12-Pp3	Pp3	China, Xinjiang, Hami, 30 km NNE	43.097°	93.598°
Pygeretmus platyurus	Ppla K12-297	Turgay K12-297	Kazakstan, Turgay		
	Ppla K12-302	Turgay K12-302			
Pygeretmus shitkovi	Pshi Kb16-122	ZMMU S197354	Kazakhstan, Almaty	46.339°	81.314°

	Pshi Kb16-123	ZMMU S197355	Reg., Koshkarakol Lake		
	Pshi Kb16-124	ZMMU S197356			
	Pshi Zoo	ZMMU S196141	Kazakhstan, E of Almaty, near Shilik		
Scarturus hotsoni	Ahot 34SP	34SP-AM	Iran, Semnan, Parvar, Damghan	36.178°	54.323°
Scarturus e. elater	Aela Dag30	uncatalogized (coll. L.Khlyap)	Russia, Dagestan, Chakanny, Kizlyar district	43.792°	47.522°
	Aela Kb16-26	ZMMU S197245	Kazakhstan, Qaraghandy Reg., NW shore of Lake Balkhash, 30 km SW from Gulshad vill.	46.385°	74.017°
Scarturus e. caucasicus	Aela G17-1	1G	Georgia, Kakhet'i Dalis Mta Reservoir, Iori River v.	41.282°	45.849°
Scarturus e. strandi	Aest Uz17-19	ZMMU S198800	Uzbekistan, Navoi reg., 23 km NW Zarafshan, Zhamankum Sands	41.737°	64.024°
Scarturus e. strandi	Aest Uz17-82	ZMMU S198817	Uzbekistan, Bukhara reg., Bukhara Hazard cattery	39.588°	64.660°
	Aest 04SK	04SK-AM	Iran, Razavi Khorasan, Khaf	34.572°	60.157°
	Aest 05SK	05SK-AM			
	Aest 07SK	07SK-AM	Iran, South Khorasan, Ghaen	33.724°	59.239°
	Aest 08SK	08SK-AM			
Scarturus e. dzungariae	Aedz Xin12-Ae1	Ae1	China, Xinjiang, Musowan Field Station, 1.5 km NE	45.126°	86.028°
	Aedz M15-131	ZMMU S196374	Mongolia, Khovd aimak, Bayan-Inder, 37 km SW from Uench	45.812°	91.696°

Scarturus e. zaisanicus	Aeza K13-24	ZMMU S192593	Kazakhstan, E Kazakhstan reg., W of Tugyl	47.771°	84.166°
Scarturus indicus toussi	Aind 33SP	33SP-AM	Iran, Semnan, Parvar		
	Aind 35EN	35EN-AM	Iran, Semnan, Parvar, Damghan	36.178°	54.323°
	Aind 06SK	06SK-AM	Iran, Razavi Khorasan, Zaveh	35.269°	59.459°
Scarturus i. aralychensis	Aara Arm	ZMMU S197177	Armenia, between Artashat and Vedi	39.931°	44.644°
	Aara 41HA	41HA-AM	Iran, Hamadan-Kabudar Ahang, Akanlu	35.601°	48.189°
	Aara 09HK	09HK-AM			
Scarturus heptneri	Ahep K15-7	ZMMU S196178	Kazakhstan, Almaty region, S Balkhash, Bozbor	45.257°	75.425°
	Ahep Uz17-75	ZMMU S198813	Uzbekistan, Bukhara region, S from Kuldzhuktau	40.751°	63.829°
	Ahep Uz17-80	ZMMU S198815	Uzbekistan, Bukhara reg., Bukhara Hazard cattery	39.588°	64.660°
	Ahep 39NK	39NK-AM	Iran, Razavi Khorasan, Sarakhs	36.538°	61.058°
	Ahep 01RK	01RK-AM	Iran, Razavi Khorasan, Shurak Maleki	36.069°	60.228°
	Ahep 02RK	02RK-AM			
	Ahep 03RK	03RK-AM			
Scarturus euphraticus	Aeup BK5	BK5	Turkey, Sanliurfa, Harran	36.836°	39.199°
	Aeup BK6	BK6			
Scarturus aulacotis	Aaul BK23	BK23	Syria, Karyatien, Homs	34.246°	37.230°
	Aaul BK28	BK28			
Scarturus williamsi	Awil BK1	BK1	Turkey, Konya, Cihanbeyli	38.551°	33.181°

	Awil BK3	BK3	Turkey, Kirikkale, Keskin	39.685°	33.629°
	Awil BK29	115/11BK	Lebanon, Beqaa, Aarsal	34.171°	36.417°
	Awil 10EA	10EA	East Azerbaijan, Ahar	38.469°	47.040°
	Awil 11EA	11EA			
Scarturus caprimulga	Acap BK12*	ZFMK 93.639	Afganistan, Ghazni, Dashte Nawar	33.686°	67.841°
Scarturus sp. Kopet Dag (S. aff. *caprimulga*)	Asp IR1	ZMFUM 5028/5029	Iran, Khorasan-e Razavi, Kopet Dag	36.935°	59.520°
Scarturus vinogradovi	Avin 148087*	ZMMU S148087	Kyrgyzstan, Talas district, Kirovskoye (1989)	42.643°	71.582°
Cardiocranius paradoxus	M10-137	ZMMU S187344	Mongolia, Govi-Altai aymag, Buutsagaan ~50km SW	45.951°	98.074°
Salpingotus kozlovi	M13-28	ZMMU S183051	Mongolia, Govi-Altai aymag, Barun-Shargyn-Gol	43.435°	97.224°
Euchoreutes naso	M05-129	ZMMU S179636	Mongolia, Bayankhongor aymag, Ekhiin-gol 47 km NE	43.466°	99.483°
Dipus sagitta	M18-182	M18-182	Mongolia, Burdene-Bulag, 120 km from Saishand	44.217°	110.933°
	M17-13	ZMMU S198673	Mongolia, Ubs aymag, Altynels	49.783°	95.383°

ZMMU – Zoological Museum of Moscow State University, Moscow
ZFMK – Zoological Research Museum Alexander Koenig Leibniz Institute for Animal Biodiversity
Asterisks mark the specimens which DNA was extracted from dried skins or bones of museum collection

Table S2. Sequences retrieved from GeneBank.

species	Tissue code/voucher	Genes/GB AccNo	reference
		**Nuclear genes**	
Allactaga major	Am Dag	***IRBP:*** *JQ347931*<br>***GHR***: *JQ347911*<br>***BRCA1***: *JQ347889*<br>***RAG1***: *JQ347867*	[1]
Allacatadipus bobrinskii	Abob Turkm	***IRBP:*** *JQ347934*<br>***GHR***: *JQ347914*<br>***BRCA1***: *JQ347892*<br>***RAG1***: *JQ347870*	[1]
		A2AB: JF938721	[2]
Orientallactaga balikunica	Abal 05-125	***IRBP:*** *KM397136*<br>***GHR***: *KM397227*<br>***BRCA1***:*KM397274*<br>***RAG1***: *KM397274*	[3]
O. bullata	Abull M05-141	***IRBP***: *JQ347929*<br>***GHR***:*JQ347909*<br>***BRCA1***: *JQ347887*<br>***RAG1***: *JQ347865*	[1]
O. sibirica	Asib M06-164	***IRBP:*** *JQ347928*<br>***GHR:*** *JQ347908*<br>***BRCA1***:*JQ347886*<br>***RAG1:*** *JQ347864*	[1]
Scarturus e. elater	Aela Dag30	***IRBP***: JQ347933<br>***GHR:*** JQ347913<br>***BRCA1:*** JQ347891<br>***RAG1***: JQ347869	[1]
S. e. caucasicus	Aela G17-1	***IRBP***: *MH979213*<br>***BRCA1***: *MH991700*	[4]
S. vinogradovi	Avin	***IRBP***: *MH979217*	[4]
S. i. aralychensis	Aara Arm	***IRBP***: *MH979161*<br>***BRCA1***: *MH991640*	
S. e. strandi	Aest Uz17-19	***IRBP***: *MH979197*<br>***BRCA1:*** *MH991683*	
	Aest Uz17-82	***IRBP***: *MH979211*<br>***BRCA1***: *MH991697*	
S. e. dzungariae	Aedz Xin12-1	***IRBP***: MH979164<br>***BRCA1:*** MH991644	
S. heptneri	Ahep K15-7	***IRBP***: *MH979165*<br>***BRCA1***: *MH991651*	
	Ahep Uz17-75	***BRCA1***:*MH991694*<br>***IRBP***: *MH979208*	
	Ahep Uz17-80	***BRCA1***: *MH991695*	

		IRBP: *MH979209*	
S. hotsoni	Ahot GB	***A2AB:*** JF938728<br>***ATP7a:*** JF938754<br>***BRCA1:*** JF938780<br>***CNR1:*** JF938807<br>***CREM:*** JF938830<br>***GHR:*** JF938857<br>***IRBP***: JF938883<br>**RAG2:** JF938906<br>***vWF:*** JF938705	[2]
S. tetradactylus		***A2AB:*** KT164755<br>***IRBP***: KT164758<br>***GHR:*** KT164757<br>***BRCA1:*** KT164760<br>***vWF:*** KT164761<br>***LCAT:*** KT164759<br>***ATP7a:*** KT164762<br>***CNR1:*** KT164756<br>***CREM:*** KT164763<br>***RAG2:*** KT164764	[5]
Dipus sagitta		***BRCA1:*** *JQ347896*<br>***RAG1:*** *JQ347874*	[1]
		A2AB: JF938720<br>***ATP7a:*** JF938746<br>***CREM:*** JF938822	[2]
		IRBP: AJ427232<br>***LCAT***: AJ275532	[6]
		GHR: AM407908	[7]
		CNR1: AY303184<br>***RAG2:*** AY303203	[8]
Jaculus jaculus		***GHR:*** XM_004652494<br>***RAG1:*** XM_004651251<br>***ACP5:*** NW_004504415<br>***c-myc:*** NW_004504331<br>***LCAT***:NW_004504357<br>**ATP7a:**XM_004666975	whole genome shotgun sequence JacJac1.0
		IRBP: AM407907	[7]
		BRCA1: JN414198<br>***BRCA2:*** JN414342<br>***CREM:*** JN633508<br>***ApoB:*** JN414058	[9]

		A2AB:JF938725	2
		CNR1: FM162121<br>***RAG2***: FM162089	10
		vWF: AJ224665	11
Cardiocranius paradoxus		***IRBP***: *JQ347925*<br>***GHR***: *JQ347905*<br>***BRCA1***: *JQ347883*<br>***RAG1***: *JQ347861*	1
		A2AB: JF938732<br>***ATP7***: JF938758<br>***CNR1***: JF938811<br>***CREM***: JF938834<br>***vWF:*** JF938709	2
Salpingotus kozlovi		***IRBP***: *JQ347924*<br>***GHR***: *JQ347904*<br>***BRCA1***: *JQ347882*<br>***RAG1***: *JQ347860*	3
		A2AB: JF938729<br>***ATP7***: JF938755<br>***CNR1***: JF938808<br>***CREM***: JF938831<br>***vWF:*** JF938706	2
Euchoreutes naso		***IRBP***: *JQ347927*<br>***GHR***: *JQ347907*<br>***BRCA1***: *JQ347885*<br>***RAG1***: *JQ347863*	1
		A2AB: JF938724<br>***ATP7***: JF938750<br>***CNR1***: JF938803<br>***CREM***: JF938826<br>***RAG2***: JF938902<br>***vWF:*** JF938702	2
		cytb	
Allactaga major	Am Dag	*KM397178*	3
	Am Kb16-23	*MH973322*	4
Allactaga severtzovi	Asev Kb16-111	*MH973323*	4
O. balikunica	YW15014	MN979455	12
	XJ08106	MN979456	
	XJ08107	MN979457	
	Abal M05-125	*KM397180*	3
O. bullata	Abull M05-141	*KM397179*	3
Allactodipus	Abob Turkm	*KM397181*	3

bobrinskii			
Pygeretmus pumilio	Ppum Kb16-5AR	*MH973415*	[4]
Pygeretmus shitkovi	Pzh Kb16-124	*MH973414*	[4]
O. sibirica	QZ0005	HQ585979	[2]
	H1− H9	JX891482 −JX891490	[13]
	XJ0309SD03	*KM397214*	[3]
	CGGZ02, CGGZ03, CGJY01−CGJY10, CJTJ01−CJTJ06, CNAY01, CNAZ04−CNAZ08, CNBLY01−CNBLY07, CNCTS01−CNCTS04, CNDM01−CNDM11, CNEL01−CNEL03, CNET01−CNET03, CNHH01, CNHLX01, CNKL01−CNKL05, CNWC01, CNWC02, CNWH01−CNWH05, CNWS01−CNWS22, CNWZ01, CNXLDW01, CNXLW01−CNXLW08, CNXYC01, CNZL01, CQDL2−CQDL41, CQDLC01−CQDLC06, CQGQ01−CQGQ04, CQGY01−CQGY04, CQWD01,CQWD02, CXAL01, CXBE01,CXBE02, CXBL01−CXBL11, CXBT01−CXBT14; CXCMJJ01CXCMJJ07, CXCML01−CXCML13, CXCQ01; CNAY01, CXFHDN01−CXFHDN07, −CXFHS01− CXFHS03, CXFJ01, CXFJ02; CXFYB01−CXFYB13, CXHBH01−CXHBH11, CXHYW01, CXHYW01; CXJMN01−CXJMN06, CXKL01−CXKL03, CXQH01−CXQH04, CXTEM01−CXTEM06, CXTHF18401−CXTHF18406, CXWE01−CXWE03,	MN979460−MN979461, MN979462−MN979471, MN979472−MN979477, MN979479MN979483, MN979484−MN979490, MN979491−MN979494, MN979495MN979505, MN979506−MN979508, MN979509−MN979511 MN979512, MN979513, MN979514−MN979518, MN979519, MN979520 MN979521−MN979525, MN979526−MN979547, MN979548, MN979549 MN979550−MN979557 MN979558, MN979559 MN979560−MN979599, MN979600−MN979605, MN979606−MN979609, MN979610−MN979613, MN979614−MN979616 MN979617−MN979618, MN979619−MN979629, MN979630−MN979743, MN979644−MN979650, MN979651−MN979663, MN979664, MN979478, MN979665−MN979679, MN979680−MN979682, MN979683, MN979684 MN979685−MN979698, MN979699−MN979709, MN979710, MN979711 MN979712−MN979717, MN979718−MN979720, MN979721−MN979724, MN979725−MN979731, MN979732−MN979737, MN979738−MN979740,	[12]

	CXWFC01−CXWFC03, MBEG01, MHHA01	MN979741−MN979743, MN979744, MN979745	
S. vinogradovi	Avin S148087	*MH973407*	4
Scarturus i. indicus	Aind BK16 Afganistan	*MH973413*	4
Scarturus i. toussi	FUMZM1431	JQ954933	14
	FUMZM1416	JQ954938	
	FUMZM2694−FUMZM2696	JQ954954−JQ954956	
	FUMZM1415, FUMZM1418, FUMZM1430	JQ954957−JQ954959	
	14, 17, 18, 21	KX018304−KX018307	15
	ZMFUM2864,ZMFUM3557, ZMFUM3558,ZMFUM3553, ZMFUM2875,ZMFUM1434, ZMFUM1438, ZMFUM3614	KX219828−KX219835	16
	-	AJ389534	17
S. i. aralychensis	Aara Arm	*MH973325*	4
	FUMZM2678−FUMZM2681	JQ954934−JQ954937	14
	ZMFUM4503	KX219837	15
	ECHAP1	MG255325	18
Scarturus e. elater	Aela Dag30	*KM397178*	3
	Aela Kb16-75	*MH973355*	4
	Aela K12-201	*MH973326*	
	Aela K12-266	*MH973347*	
	Aela Kb16-108	*MH973357*	
	Aela Kb16-34	*MH973349*	
	Aela Kb16-40	*MH973368*	
	Aela Kb16-41	*MH973350*	
	Aela Kb16-42	*MH973351*	
	Aela Kb16-71	*MH973354*	
	Aela Kb16-73	*MH973365*	
	Aela Kb16-76	*MH973366*	
	Aela Kb16-84	*MH973356*	
	Aela Kalmykia 2219	*MH973401*	
	Aela Uz17-33	*MH973385*	
	Aela Uz17-34	*MH973386*	
	Aela Zoo	*MH973324*	
	Aela Kb16-26	*MH973348*	
S. e. zaisanicus	Aela K12-66	*MH973327*	4
	Aela K12-67	*MH973328*	
	Aela K12-74	*MH973329*	
	Aela K13-24	*MH973339*	
	Aela K13-25	*MH973340*	
	Aela K13-26	*MH973341*	
	Aela K13-27	*MH973342*	

	Aela K13-28	*MH973343*	
	Aela K13-49	*MH973344*	
	Aela K13-50	*MH973345*	
	Aela K13-84	*MH973346*	
S. e. caucasicus	Aela G17-2	*MH973399*	
	Aela G17-3	*MH973400*	
	Aela G17-1	*MH973398*	
S. e. strandi	Aest Uz17-19	*MH973383*	4
	Aest Uz17-82	*MH973394*	
	Aest Uz17-05	*MH973378*	
	Aest Uz17-07	*MH973380*	
	Aest Uz17-08	*MH973381*	
	Aest Uz17-09	*MH973402*	
	Aest Uz17-23	*MH973384*	
	Aest Uz17-36	*MH973403*	
	Aest Uz17-44	*MH973388*	
	Aest Uz17-55	*MH973389*	
	Aest Uz17-71	*MH973391*	
	Aest Uz17-74	*MH973392*	
	Aest Uz17-83	*MH973395*	
	FUMZM 2674	JQ954928	
	FUMZM 2128	JQ954931	
	FUMZM 1429	JQ954932	
	ZMFUM1377,ZMFUM1412, ZMFUM2084,ZMFUM1740, ZMFUM3542,ZMFUM1905, ZMFUM2704,ZMFUM2749, ZMFUM2842,ZMFUM2862, ZMFUM1374,ZMFUM2897	KX219804− KX219815	15
S. e. dzungariae	Aedz Xin12-1	*MH973330*	4
	XJ18107	*MN979459*	
	Aedz M15-131	*MH973335*	
S. euphratica	FUMZM 2129	JQ954953	14
	Har1− Har4	KC465442− KC465445	19
	ECHAP3− ECHAP11	MG255326−MG255334	18
S. aulacotis	Kar1− Kar4, Dam1	KC465446−KC465450	19
S. williamsi	2688−2139	JQ954944− JQ954952	14
	Kon1, Kon2, Kes1, Leb1, Leb2	KC465439− C465441, KC465451− KC465452	19
	ECHAP12− ECHAP86	MG255335−MG255409	18
S. heptneri	Ahep K15-22	*MH973333*	4
	Ahep K15-27	*MH973334*	
	Ahep K15-8	*MH973332*	
	Ahep Kb16-109	*MH973358*	

	Ahep Kb16-118	*MH973359*	
	Ahep Kb16-121	*MH973360*	
	Ahep Kb16-126	*MH973367*	
	Ahep Kb16-130	*MH973361*	
	Ahep Kb16-135	*MH973362*	
	Ahep Kb16-136	*MH973363*	
	Ahep Kb16-1AR	*MH973336*	
	Ahep Kb16-2AR	*MH973337*	
	Ahep Kb16-43	*MH973369*	
	Ahep Kb16-44	*MH973370*	
	Ahep Kb16-45	*MH973371*	
	Ahep Kb16-46	*MH973372*	
	Ahep Kb16-49	*MH973352*	
	Ahep Kb16-50	*MH973373*	
	Ahep Kb16-51	*MH973374*	
	Ahep Kb16-53	*MH973375*	
	Ahep Kb16-54	*MH973376*	
	Ahep Kb16-55	*MH973353*	
	Ahep Kb16-60	*MH973377*	
	Ahep Kb16-61	*MH973364*	
	Ahep Kb16-6AR	*MH973338*	
	Ahep Uz17-06	*MH973379*	
	Ahep Uz17-1479	*MH973396*	
	Ahep Uz17-1480	*MH973397*	
	Ahep Uz17-18	*MH973382*	
	Ahep Uz17-37	*MH973387*	
	Ahep Uz17-66	*MH973404*	
	Ahep Uz17-68	*MH973390*	
	Ahep Uz17-75	*MH973393*	
	Ahep Uz17-80	*MH973405*	
	Ahep Uz17-81	*MH973406*	
	Ahep Zoo2013	*MH973331*	
	FUMZA 2675	JQ954927	[14]
	FUMZM 2676	JQ954929	
	FUMZM 2677	JQ954930	
	ZMFUM2732,ZMFUM2738, ZMFUM2751,ZMFUM3547, ZMFUM3548,ZMFUM3554, ZMFUM3601−ZMFUM3609 ZMFUM3613	KX219816−KX219827	[15]
S. hotsoni	11, 12, 13, 15, 16, 19, 20−24	KX018295−KX018303	[15]
	FUMZM 2682, FUMZM 2684, FUMZM 2686, FUMZM 2687, FUMZM 2685, FUMZM 1277,	JQ954939−JQ954943, JQ954960−JQ954962	

	FUMZM 1521, FUMZM 1533		
Dipus sagitta	ZMMU S179627	*MF076858*	[20]
	M13-149	*MF535703*	[21]
Jaculus jaculus	sc27,<br>sc31	JN214514, JN214518	[22]
Cardiocranius paradoxus	ZMMU S188830	*KM397211*	[3]
Salpingotus kozlovi	M13-28 / ZMMU S183051	*KM397210*	[3]
Euchoreutes naso	M05-129 / ZMMU S179636	*KM397212*	[3]
	-	KP981634	[23]

Supplementary Table S3. GenBank Accession Numbers for the original sequences used in the study.

Asterisks denote sequences obtained in the previous studies (for information see Table S2).

species	code	*IRBP*	*GHR*	*BRCA1*	*RAG1*	*RAG2*	*BRCA2*	*APOB*	*vWF*	*LCAT*	*CNR1*	*ACP5*	*ATP7a*	*A2AB*	*CREM*	*cmyc*	*cytb*
Allactaga major	Am Dag	***	***	***	***	OL494491	OL494645	OL494702	OL494991	OL494932	OL494821	OL494762	OL494875	OL494377	OL495202	OL494431	***
A. major	Am K13-83	OL495161	OL494595	OL495048	OL494548	OL494492	OL494646	OL494703	OL494992	OL494933	OL494822	OL494763	OL494876	OL494378	OL495203	OL494432	OL495088
A. major	Am Kb16-23	OL495162	OL494596	OL495049	OL494549	OL494493	OL494647	OL494704	OL494993	OL494934	OL494823	OL494764	OL494877	OL494379	OL495204	-----	***
A. major	Am Kb16-56	OL495163	OL494597	OL495050	OL494550	OL494494	OL494648	OL494705	OL494994	OL494935	OL494824	OL494765	OL494878	OL494380	OL495205	OL494433	OL495089
A. major	Am 4	-----	-----	-----	-----	-----	-----	-----	-----	-----	-----	-----	-----	-----	-----	-----	OL495090
A. major	Am K12-263	-----	-----	-----	-----	-----	-----	-----	-----	-----	-----	-----	-----	-----	-----	-----	OL495091
A. major	Am K12-274	-----	-----	-----	-----	-----	-----	-----	-----	-----	-----	-----	-----	-----	-----	-----	OL495092
A. major	Am K12-295	-----	-----	-----	-----	-----	-----	-----	-----	-----	-----	-----	-----	-----	-----	-----	OL495093
A. major	Am spiculum 143270	-----	-----	-----	-----	-----	-----	-----	-----	-----	-----	-----	-----	-----	-----	-----	OL495094
A. severtzovi	Asev Kb16-110	OL495164	OL494598	OL495051	OL494551	OL494495	OL494649	OL494706	OL494995	OL494936	OL494825	OL494766	OL494879	OL494381	OL495206	OL494434	OL495095
A. severtzovi	Asev Uz17-40	OL495165	OL494599	OL495052	OL494552	OL494496	OL494650	OL494707	OL494996	OL494937	OL494826	OL494767	OL494880	OL494382	OL495207	OL494435	OL495096
A. severtzovi	Asev Uz17-88	OL495166	OL494600	OL495053	OL494553	OL494497	OL494651	OL494708	OL494997	OL494938	OL494827	OL494768	OL494881	OL494383	OL495208	OL494436	OL495097
A. severtzovi	Asev 2006	-----	-----	-----	-----	-----	-----	-----	-----	-----	-----	-----	-----	-----	-----	-----	OL495098
Allactodipus bobrinskii	Abob Turkm	***	***	***	***	OL494498	OL494652	OL494709	OL494998	OL494939	OL494828	OL494769	OL494882	***	OL495209	OL494437	***
A. bobrinskii	Abob-Uz17-21	OL495167	OL494601	OL495054	OL494554	OL494499	OL494653	OL494710	OL494999	OL494940	OL494829	OL494770	OL494883	OL494384	OL495210	OL494438	OL495099
Orientallactaga sibirica	Asib Bur01-22	OL495168	OL494602	OL495055	OL494555	OL494500	OL494654	OL494711	OL495000	OL494941	OL494830	OL494771	OL494884	OL494385	OL495211	OL494439	OL495100
O. sibirica	Asib M18-114	OL495169	OL494603	OL495056	OL494556	OL494501	OL494655	OL494712	OL495001	OL494942	OL494831	OL494772	OL494885	OL494386	OL495212	OL494440	OL495101
O. sibirica	Asib Kb16-03	OL495170	OL494604	OL495057	OL494557	OL494502	OL494656	OL494713	OL495002	OL494943	OL494832	OL494773	OL494886	OL494387	OL495213	OL494441	OL495102
O. sibirica	Asib Kb16-140	OL495171	OL494605	OL495058	OL494558	OL494503	OL494657	OL494714	OL495003	OL494944	OL494833	OL494774	OL494887	OL494388	OL495214	OL494442	OL495103
O. sibirica	Asib M10-185	OL495172	OL494606	OL495059	OL494559	OL494504	OL494658	OL494715	OL495004	OL494945	OL494834	OL494775	OL494888	OL494389	OL495215	OL494443	OL495104
O. sibirica	Asib M13-187	OL495173	OL494607	OL495060	OL494560	OL494505	OL494659	OL494716	OL495005	OL494946	OL494835	OL494776	OL494889	OL494390	OL495216	OL494444	OL495105
O. sibirica	Asib M06-164	***	***	***	***	OL494506	OL494660	OL494717	OL495006	OL494947	OL494836	OL494777	OL494890	OL494391	OL495217	OL494445	***
O. sibirica	Asib Xin12-As1	-----	-----	-----	-----	-----	-----	-----	-----	-----	-----	-----	-----	-----	-----	-----	OL495107
O. sibirica	Asib M12-68	-----	-----	-----	-----	-----	-----	-----	-----	-----	-----	-----	-----	-----	-----	-----	OL495108
O. sibirica	Asib M14-64	-----	-----	-----	-----	-----	-----	-----	-----	-----	-----	-----	-----	-----	-----	-----	OL495109
O. sibirica	Asib M14-86	-----	-----	-----	-----	-----	-----	-----	-----	-----	-----	-----	-----	-----	-----	-----	OL495259
O. sibirica	Asib M14-171	-----	-----	-----	-----	-----	-----	-----	-----	-----	-----	-----	-----	-----	-----	-----	OL495110
O. sibirica	Asib M14-144	-----	-----	-----	-----	-----	-----	-----	-----	-----	-----	-----	-----	-----	-----	-----	OL495111
O. sibirica	Asib 146874	-----	-----	-----	-----	-----	-----	-----	-----	-----	-----	-----	-----	-----	-----	-----	OL495112
O. sibirica	Asib 73068	-----	-----	-----	-----	-----	-----	-----	-----	-----	-----	-----	-----	-----	-----	-----	OL495113
O. sibirica	Asib 69280	-----	-----	-----	-----	-----	-----	-----	-----	-----	-----	-----	-----	-----	-----	-----	OL495114
O. sibirica	Asib 145985	-----	-----	-----	-----	-----	-----	-----	-----	-----	-----	-----	-----	-----	-----	-----	OL495115
O. sibirica	Asib 73076	-----	-----	-----	-----	-----	-----	-----	-----	-----	-----	-----	-----	-----	-----	-----	OL495116
O. sibirica	Asib 73081	-----	-----	-----	-----	-----	-----	-----	-----	-----	-----	-----	-----	-----	-----	-----	OL495117
O. sibirica	Asib 146877	-----	-----	-----	-----	-----	-----	-----	-----	-----	-----	-----	-----	-----	-----	-----	OL495118
O. sibirica	Asib 143272	-----	-----	-----	-----	-----	-----	-----	-----	-----	-----	-----	-----	-----	-----	-----	OL495119
O. sibirica	Asib 57308	-----	-----	-----	-----	-----	-----	-----	-----	-----	-----	-----	-----	-----	-----	-----	OL495120
O. bullata	Abull M14-173	OL495174	OL494608	OL495061	OL494561	OL494507	OL494661	OL494718	OL495007	OL494948	OL494837	OL494778	OL494891	OL494392	OL495218	OL494446	OL495121
O. bullata	Abull M05-141	***	***	***	***	OL494508	OL494662	OL494719	OL495008	OL494949	OL494838	OL494779	OL494892	OL494393	OL495219	OL494447	***
O. bullata	Abull M12-97	-----	-----	-----	-----	-----	-----	-----	-----	-----	-----	-----	-----	-----	-----	-----	OL495122
O. bullata	Abull M12-98	-----	-----	-----	-----	-----	-----	-----	-----	-----	-----	-----	-----	-----	-----	-----	OL495123
O. bullata	Abull M13-147	-----	-----	-----	-----	-----	-----	-----	-----	-----	-----	-----	-----	-----	-----	-----	OLXXXX
O. bullata	Abull EG	-----	-----	-----	-----	-----	-----	-----	-----	-----	-----	-----	-----	-----	-----	-----	OL495124
O. balikunica	Abal M05-125	***	***	***	***	OL494509	-----	OL494720	OL495009	OL494950	-----	OL494780	OL494893	OL494394	OL495220	OL494448	***
O. balikunica	Abal M13-77	OL495175	OL494609	OL495062	OL494562	OL494510	OL494663	OL494721	OL495010	OL494951	OL494839	OL494781	OL494894	OL494395	OL495221	OL494449	OL495125
O. balikunica	Abal M14-231	OL495176	OL494610	OL495063	OL494563	OL494511	OL494664	OL494722	OL495011	OL494952	OL494840	OL494782	OL494895	OL494396	OL495222	OL494450	OL495126
O. balikunica	Abal M13-147	-----	-----	-----	-----	-----	-----	-----	-----	-----	-----	-----	-----	-----	-----	-----	OL495127
O. balikunica	Abal M11-226	-----	-----	-----	-----	-----	-----	-----	-----	-----	-----	-----	-----	-----	-----	-----	OL495128

O. balikunica	Xin12-Ab1	-----	-----	-----	-----	-----	-----	-----	-----	-----	-----	-----	-----	-----	-----	-----	OL495129
Pygeretmus pumilio	Ppum K12-68	OL495177	OL494611	OL495064	OL494564	OL494512	OL494665	OL494723	OL495012	OL494953	OL494841	OL494783	OL494896	OL494397	OL495223	OL494451	OL495130
P. pumilio	Ppum Uz17-51	OL495178	OL494612	OL495065	OL494565	OL494513	OL494666	OL494724	OL495013	OL494954	OL494842	OL494784	OL494897	OL494398	OL495224	OL494452	OL495131
P. pumilio	Ppum M06-153	***	***	***	***	OL494514	OL494667	OL494725	OL495014	OL494955	OL494843	OL494785	OL494898	OL494399	OL495225	OL494453	***
P. pumilio	Ppum M11-100			-----	-----	-----	-----	-----	-----	-----	-----	-----	-----	-----	-----	-----	OL495132
P. pumilio	Ppum K12-245			-----	-----	-----	-----	-----	-----	-----	-----	-----	-----	-----	-----	-----	OL495133
P. pumilio	Ppum K12-251			-----	-----	-----	-----	-----	-----	-----	-----	-----	-----	-----	-----	-----	OL495134
P. pumilio	Ppum K12-261			-----	-----	-----	-----	-----	-----	-----	-----	-----	-----	-----	-----	-----	OL495135
P. pumilio	Ppum K12-262			-----	-----	-----	-----	-----	-----	-----	-----	-----	-----	-----	-----	-----	OL495136
P. pumilio	Ppum K12-204			-----	-----	-----	-----	-----	-----	-----	-----	-----	-----	-----	-----	-----	OL495137
P. pumilio	Ppum Xin12-Pp3			-----	-----	-----	-----	-----	-----	-----	-----	-----	-----	-----	-----	-----	OL495138
P. pumilio	Ppum Uz17-54	-----	-----	-----	-----	-----	-----	-----	-----	-----	-----	-----	-----	-----	-----	-----	OL495139
P. platyurus	Ppla K12-297	OL495179	OL494613	OL495066	OL494566	OL494515	OL494668	OL494726	OL495015	OL494956	OL494844	OL494786	OL494899	OL494400	OL495226	OL494454	OL495258
P. platyurus	Ppla K12-302	OL495180	OL494614	OL495067	OL494567	OL494516	OL494669	OL494727	OL495016	OL494957	OL494845	OL494787	OL494900	OL494401	OL495227	OL494455	OL495140
P. shitkovi	Pshi Kb16-122	OL495181	OL494615	OL495068	OL494568	OL494517	OL494670	OL494728	OL495017	OL494958	OL494846	OL494788	OL494901	OL494402	OL495228	OL494456	OL495141
P. shitkovi	Pshi Zoo	OL495182	OL494616	OL495069	OL494569	OL494518	-----	OL494729	OL495018	OL494959	OL494847	-----	OL494902	OL494403	OL495229	OL494457	OL495142
P. shitkovi	Pshi Kb16-123	-----	-----	-----	-----	-----	-----	-----	-----	-----	-----	-----	-----	-----	-----	-----	OL495143
P. shitkovi	Pshi Kb16-124	-----	-----	-----	-----	-----	-----	-----	-----	-----	-----	-----	-----	-----	-----	-----	OL495144
Scarturus williamsi	Awil BK1	OL495183	OL494617	OL495070	OL494570	OL494519	OL494671	OL494730	OL495019	OL494960	OL494848	OL494789	OL494903	OL494404	OL495230	OL494458	***
S. williamsi	Awil BK3	OL495184	OL494618	OL495071	OL494571	OL494520	OL494672	OL494731	OL495020	OL494961	OL494849	OL494790	OL494904	OL494405	OL495231	OL494459	***
S. williamsi	Awil BK29	OL495185	OL494619	OL495072	OL494572	OL494521	OL494673	OL494732	OL495021	OL494962	OL494850	OL494791	OL494905	OL494406	OL495232	OL494460	***
S. williamsi	Awil 10EA	OL495186	OL494620	OL495073	OL494573	OL494522	OL494674	OL494733	OL495022	OL494963	OL494851	OL494792	OL494906	OL494407	OL495233	OL494461	-----
S. williamsi	Awil 11EA	OL495187	OL494621	OL495074	OL494574	OL494523	OL494675	OL494734	OL495023	OL494964	OL494852	OL494793	OL494907	OL494408	OL495234	OL494462	OL495145
S. euphraticus	Aeup BK5	OL495188	OL494622	OL495075	OL494575	OL494524	OL494676	OL494735	OL495024	OL494965	OL494853	OL494794	OL494908	OL494409	OL495235	OL494463	***
S. euphraticus	Aeup BK6	OL495189	OL494623	OL495076	OL494576	OL494525	OL494677	OL494736	OL495025	OL494966	OL494854	OL494795	OL494909	OL494410	OL495236	OL494464	***
S. aulacotis	Aaul BK23	OL495190	OL494624	OL495077	OL494577	OL494526	OL494678	OL494737	OL495026	OL494967	OL494855	OL494796	OL494910	OL494411	OL495237	OL494465	***
S. aulacotis	Aaul BK28	OL495191	OL494625	OL495078	OL494578	OL494527	OL494679	OL494738	OL495027	OL494968	OL494856	OL494797	OL494911	OL494412	OL495238	OL494466	***
S. elater elater	Aela Dag30	***	***	***	***	OL494528	OL494680	OL494739	OL495028	OL494969	OL494857	OL494798	OL494912	OL494413	OL495239	OL494467	***
S. e. elater	Aela Kb16-26	OL495192	OL494626	OL495079	OL494579	OL494529	OL494681	OL494740	OL495029	OL494970	OL494858	OL494799	OL494913	OL494414	OL495240	OL494468	***
S. e. zaisanicus	Aeza K13-24	***	OL494627	***	OL494580	OL494530	OL494682	OL494741	OL495030	OL494971	OL494859	OL494800	OL494914	OL494415	OL495241	OL494469	***
S. e. dzungariae	Aedz Xin12-Ae1	***	OL494632	***	OL494585	OL494535	OL494687	OL494746	OL495035	OL494976	OL494864	OL494805	OL494919	OL494420	OL495246	OL494474	***
S. e. dzungariae	Aedz M15-131	OL495194	OL494633	OL495081	OL494586	OL494536	OL494688	OL494747	OL495036	OL494977	OL494865	OL494806	OL494920	OL494421	OL495247	OL494475	***
S. e. caucasicus	Aela G17-1	***	OL494636	***	OL494588	OL494539	OL494691	OL494750	OL495039	OL494980	OL494868	OL494809	OL494923	OL494424	OL495250	OL494478	***
S. e. strandi	Aest Uz17-19	***	OL494638	***	OL494590	OL494541	OL494693	OL494752	OL495041	OL494982	OL494870	OL494811	OL494925	OL494426	OL495252	OL494480	***
S. e. strandi	Aest Uz17-82	***	OL494639	***	OL494591	OL494542	OL494694	OL494753	OL495042	OL494983	OL494871	OL494812	OL494926	OL494427	OL495253	OL494481	***
S. e. strandi	Aest 04SK	-----	-----	-----	-----	-----	-----	-----	-----	-----	-----	-----	-----	-----	-----	-----	OL495153
S. e. strandi	Aest 05SK	-----	-----	-----	-----	-----	-----	-----	-----	-----	-----	-----	-----	-----	-----	-----	OL495154
S. e. strandi	Aest 07SK	OL495197	OL494640	OL495084	-----	OL494543	OL494695	OL494754	OL495043	OL494984	OL494872	OL494813	OL494927	OL494428	OL495254	OL494482	OL495155
S. e. strandi	Aest 08SK	-----	-----	-----	-----	-----	-----	-----	-----	-----	-----	-----	-----	-----	-----	-----	OL495156
S. heptneri	Ahep K15-7	***	OL494628	***	OL494581	OL494531	OL494683	OL494742	OL495031	OL494972	OL494860	OL494801	OL494915	OL494416	OL495242	OL494470	***
S. heptneri	Ahep Uz17-75	***	OL494629	***	OL494582	OL494532	OL494684	OL494743	OL495032	OL494973	OL494861	OL494802	OL494916	OL494417	OL495243	OL494471	***
S. heptneri	Ahep Uz17-80	***	OL494630	***	OL494583	OL494533	OL494685	OL494744	OL495033	OL494974	OL494862	OL494803	OL494917	OL494418	OL495244	OL494472	***
S. heptneri	Ahep 01RK	OL495193	OL494637	OL495080	OL494584	OL494534	OL494686	OL494745	OL495034	OL494975	OL494863	OL494804	OL494918	OL494419	OL495245	OL494473	OL495146
S. heptneri	Ahep 02RK	-----	-----	-----	-----	-----	-----	-----	-----	-----	-----	-----	-----	-----	-----	-----	OL495147
S. heptneri	Ahep 03RK	-----	-----	-----	-----	-----	-----	-----	-----	-----	-----	-----	-----	-----	-----	-----	OL495148
S. heptneri	Ahep 39NK	-----	-----	-----	-----	-----	-----	-----	-----	-----	-----	-----	-----	-----	-----	-----	OL495149
S. indicus toussi	Aind 33SP	OL495199	OL494642	OL495085	OL494593	OL494545	OL494696	OL494756	OL495045	OL494986	OL494873	OL494815	OL494929	-----	OL495256	OL494484	-----
S. i. toussi	Aind 35EN	OL495200	OL494643	OL495086	OL494594	OL494546	OL494697	OL494757	OL495046	OL494987	OL494874	OL494816	OL494930	-----	OL495257	OL494485	OL495158
S .i. toussi	Aind 06SK	-----	-----	-----	-----	-----	-----	-----	-----	-----	-----	-----	-----	-----	-----	-----	OL495159
S. i. aralychensis	Aara Arm	***	OL494634	***	-----	OL494537	OL494689	OL494748	OL495037	OL494978	OL494866	OL494807	OL494921	OL494422	OL495248	OL494476	***
S. i. aralychensis	Aara 41HA	OL495195	OL494635	OL495082	OL494587	OL494538	OL494690	OL494749	OL495038	OL494979	OL494867	OL494808	OL494922	OL494423	OL495249	OL494477	OL495150
S. i. aralychensis	Aara 09HK	-----	-----	-----	-----	-----	-----	-----	-----	-----	-----	-----	-----	-----	-----	-----	OL495151
Scarturus caprimulga	Acap BK12	-----	-----	-----	-----	-----	-----	-----	-----	-----	-----	-----	-----	-----	-----	-----	OL589003
Scarturus sp. Kopet Dag<br>*(S.* aff. *caprimulga)*	Asp IR1	OL495196	OL494631	OL495083	OL494589	OL494540	OL494692	OL494751	OL495040	OL494981	OL494869	OL494810	OL494924	OL494425	OL495251	OL494479	OL495152
S. hotsoni	Ahot 34SP	OL495198	OL494641	-----	OL494592	OL494544	-----	OL494755	OL495044	OL494985	-----	OL494814	OL494928	OL494429	OL495255	OL494483	OL495157

S. vinogradovi	Avin 148087	OL495201	OL494644	OL495087	-----	-----	-----	-----	OL495047	-----	-----	-----	OL494931	OL494430	-----	OL494486	OL495160
Cardiocranius paradoxus	M10-137	***	***	***	***	OL494547	OL494698	OL494758	***	OL494988	***	OL494817	***	***	***	OL494487	***
Salpingotus kozlovi	M13-28	***	***	***	***	-----	OL494699	OL494759	***	OL494989	***	OL494818	***	***	***	OL494488	***
Euchoreutes naso	M05-129	***	***	***	***	***	OL494700	OL494760	***	OL494990	***	OL494819	***	***	***	OL494489	***
Dipus sagitta	M18-182	***	***	***	***	***	-----	OL494761	***	***	***	OL494820	***	***	***	OL494490	***
Dipus sagitta	M17-13	-----	-----	-----	-----	-----	OL494701	-----	-----	-----	-----	-----	-----	-----	-----	——	***

Supplementary methods

Molecular procedures

Modern DNA extraction, amplification, and sequencing

The total DNA of modern specimens was extracted from tissues stored in alcohol using a standard protocol of proteinase K digestion, phenol-chloroform deproteinization, and isopropanol precipitation[24]. Primers used in amplification and sequencing of *BRCA1*, *IRBP*, *GHR*, *RAG1,* and *BRCA2* nuclear genes and the PCR conditions were described in the previous studies[1,20,25]. The *cytb* gene in most species from the sample was amplified with the primers by Montgelard et al.[17] and sequenced using primers from Bannikova et al.[4]; however, primers utilized for *cytb* of *Pygeretmus* spp. and short fragments of *cytb* of the museum specimens were specifically designed for this study (SuppInfo, Table S4). The amplification conditions for the nuclear loci included the initial denaturation at 95°C for 3 min, 36 cycles of 95°C for 30 s, 57–62°C (depending on the primer) for 45 s, 72°C for 1 min, and a final extension of 72°C for 6 min.

Amplification and sequencing of historical DNA

The total DNA of 12 jerboas was extracted from dried skins or bones of museum collection specimens (age range 30-80 years): *Scarturus vinogradovi*, *S. caprimulga* Ellerman, 1948, *Allactaga major spiculum* (Lichtenstein, 1825), and nine specimens of *Orientallactaga sibirica* Forster, 1778 (SuppInfo, Table S1). The DNA was purified directly using the MiniElute PCR Purification Kit (QIAGEN) including an overnight lysis step following the manufacturer's protocol and recommendations of Yang et al.[26]. All stages of the extraction process included a negative control run in parallel. Only short fragments of *cytb* were obtained using the internal primers (Table S4) for *A. m. spiculum* (661 bp) and nine specimens of *O. sibirica* (592 bp). For *S. vinogradovi,* the fragments of seven nuclear genes were amplified and sequenced: *BRCA1* (two fragments with a total length of 541 bp), exon 10 of *GHR* (398 bp), *vWF* (491 bp), *ATP7a* (679 bp), *c-myc* (501 bp), *A2AB* (478 bp), and *IRBP* (383 bp in addition to previously sequenced 250 bp[4]). The PCR program for amplification of short fragments included the initial denaturation at 95°C for 3 min, 45 cycles at 95°C for 30 s, 52-60°C (depending on the primer) for 30 s, 72°C for 30 s, and a final extension at 72°C for 6 min. To avoid contamination, the

extraction and amplification of the DNA of museum specimens were performed in the dedicated facilities of ZMMU Laboratory of Historical DNA, exclusively equipped for the work with historical and ancient DNA, where no previous work on fresh tissues had been performed.

The PCR products were purified using a Diatom™ DNA PCR Clean-Up (Diatom, Moscow, Russia). Approximately 10−30 ng of the purified PCR product was used for sequencing with each primer with an ABI 3100-Avant Genetic Analyzer using the BigDyeTM Terminator Chemistry v. 3.1 (Applied Biosystems).

Putative cytb pseudogene in Pygeretmus platyurus

The *cytb* sequence of one of the *P. platyurus* specimens (K12-297) that was initially obtained with the L0/H0 primer combination contained a stop codon (AGA) at the position 299, and thus is likely a sequence of a nuclear pseudogene. The sequence of the second specimen (k12-302) contained multiple positions with two peaks of approximately equal intensity. Using a set of de novo designed primers (L682, H711, H828) we were able to obtain two separate sequences for K12-302, one of which was similar to that of K12-907 (*p*-distance <1%), while the other substantially diverged from it (*p*-distance =11.2%). The latter sequence contained no stop codons, frame-shift mutations, or excessive number of non-synonymous mutations, hence it was assumed to belong to the authentic mitochondrial *cytb* gene.

Sequence processing

All sequences were aligned by eye using BioEdit version 7.0.9.0[27]. Heterozygous positions (at which two peaks of approximately equal intensity are observed) were coded using the IUB ambiguity codes.

It should be noted that *RAG2* sequences obtained by Wu et al[2] for *A. major, O. sibirica, O. bullata, A. bobrinskii,* and *P. pumilio* (JF938894−JF938896, JF938899, JF938900) are highly divergent from other sequences of Dipodidae including our newly obtained

sequences for the same species (*p*-distance=11%). Thus, they likely originate from a paralog, and their inclusion in the analysis by Wu et al[2] could have affected the results.

Table S4. Primers used for amplification and sequencing.

Primers	sequence (5'→3')	comments	Reference
	***A2AB* − Alpha-2B adrenergic rec**eptor		
a2ab-11Fa	CCATCACCTTCCTCATCCTCTTCAC	All Dipodidae	This study
a2ab-15Ra	ATCAGGTAGATGCGMAGGTAGACG	All Dipodidae	This study
	***ATP7a* − ATPase copper transporting alpha, exon**		
atp7a-11Fa	TCCCTGGACAACCAAGAAGC	All Dipodidae	This study
atp7a-15Ra	CTTTCAAGACAGCATCAAATCCCAT	All Dipodidae	This study
	***CREM* − cAMP-responsive element modulator**		
Jac_F1b	AGGAACTCAAGGCCCTCAAAGAT	All Dipodidae	This study
Jac_R1b	TGGGAGAGCAAATGTCTTTCAAGGT	All Dipodidae	This study
	***vWF* von Willebrand Factor, exon 28**		
101Fa	TGTYAACCTCACCTGTGAAGCCTG	All Dipodidae	This study
R500a	GATCACRGTGACCTTCTTCTTCTTC	All Dipodidae	This study
F101g1	TGTBAACCTYACSTGTGAAGCCTG	All Dipodidae	This study
103R	GACCTTCTTCTTCTTCAAGCCCTGG	All Dipodidae	This study
	***ACP5* − acid phosphatase 5, tartrate resistant exon & intron**		
120fwd_1	CYAATGCCCCATTCCACACAGC	All Dipodidae	This study

R590a	CGCTTGGAGATCTTGGAGTAGGCA	All Dipodidae	This study
564rev_1	GCAGAGACRTTGCCAAGGTG	*Scartus* spp.	This study
	APOB **− Apolipoprotein B, exon**		
F1370a	CTGGAGAAGCCAMTCTCCRACGCAT	All Dipodidae	This study
R2440b	YGTGGAATCCAGGAAKGTTTCAAC	All Dipodidae	This study
	c-myc **− MYC proto-oncogene, bHLH transcription factor, exon**		
F2007a	GAAGATGAGGAAGAAATTGATGTTGT	All Dipodidae	This study
R2537a	CTACGCACCAGAGTTTCGCAGC	All Dipodidae	This study
	LCAT **− lecithin-cholesterol acyltransferase, exon & intron**		
R468a	CATTGATGTGCTCCAGTGTCTTATTGCT	All Dipodidae	This study
F10a	CATCCCGATYATGTCCAGYATCAAGC	All Dipodidae	This study
	IRBP **− interphotoreceptor retinoid-binding protein, exon**		
F25dip	GCGGCCATCCARCAGGTAATGAAGAG	All Dipodidae	[1]
R1175dip	GCACTGACACCTGAAACACAGAGTCCAC	All Dipodidae	[1]
F597dip (internal)	GGACATCGCCTACATCCTCAAGCAGA	All Dipodidae	[1]
R701dip (internal)	GGACACGGGCACCGTGAGGAAGAAGT	All Dipodidae	[1]
F459_Av	CGTCGTGCACGTGGACACCATCTAT	*S. vinogradovi*	[4]
R701b_Av	ACACGGGCACCGTRAGGAAGAAGT	*S. vinogradovi*	[4]

***GHR* − gGrowth hormone receptor, exon 10**			
ghr_dip_F	GCCATTCATGATAACTACAAATCTGA	All Dipodidae	[1]
ghr_dip_R	ATAGCCACAAGATGAGAGGAACTC	All Dipodidae	[1]
ghr_zap_R	ATAGCCACAAGATGCCAGGGACTC	All Dipodidae	[1]
***BRCA1* − breast cancer type 1 susceptibility protein, exon 11**			
brca_All-l135F	GAGAACAGCAGTTTATTACYCACTAAAGACAG	All Dipodidae	[1]
brca_zap100F	TGTGGCACAGATGTTCRTGCCAGCTCATTACA	All Dipodidae	[1]
brca_All_982R	CTACTGGATTCACATTTTCCTCTTTCTG	All Dipodidae	[1]
brca_zap980R	CTACTGGATTCTCACTTTCCTCTTTCTGAA	All Dipodidae	[1]
***BRCA2* − breast cancer type 2 susceptibility protein, exon**			
F1381a	TCTACTATATTAGAAGAATCAGGAAGTCAAT	All Dipodidae	[25]
R2263a	GGAGACTTTGATGTTTTTTCCACTTGC	All Dipodidae	This study
***RAG1* − recombination activating gene 1, exon**			
rag1_all-940F	GACCTGGAGAGTCCAGTGAAGTCCTTTCT	All Dipodidae	[1]
rag1_all-980F	TGAATTCCCTGATGGTRAAATGTCC	All Dipodidae	[1]
rag1-dip2117R	ACGRGTAGCATCACAAAGAGTACAAATGT	All Dipodidae	[1]
rag1-Al613R	CTGGGTAATCATCCACAGAGGAGACAAG	All Dipodidae	[1]

***RAG2* − recombination activating gene 1, exon**			
rag2_dip10F	GGAGGRCGGTCCTACATGCCTTCTAC	All Dipodidae	This study
rag2_dip806R	GGTACCCAAGTATTAATATCCACRTCACAAG	All Dipodidae	This study
***CNR1* − Cannabinoid Receptor 1, exon**			
dip_F50	GGCAGTCCCTTCCAAGAAAAGATGAC	All Dipodidae	This study
dip_F53	GTCCCTTCCAAGAAAAGATGACTGC	All Dipodidae	This study
dip_R1036	CACGGTGGAGTTCAGGAGGCAGAG	All Dipodidae	This study
zap_R1036	CACGGTGGAGTTCAGCAGGCAGAG	All Dipodidae	This study
Cytb* – cytochrome *b			
L7-fw	ACCAATGACATGAAAAATCATCGTT	All Dipodidae	[17]
H6-rev	TCTCCATTTCTGGTTTACAAGAC	All Dipodidae	[17]
H601ae (internal)	GGGTTGTTGGATCCTGTTTCGTG	All Dipodidae	[4]
L400ae (internal)	CCATGAGGACAAATATCMTTCTGAG	All Dipodidae	[4]
L543_Av	CCACTTCATCCTCCCATTCATTGT	*S. vinogradovi, S. caprimulga*	This study
L298_Al	CGAGGACTATACTACGGMTCATACAC	*S. vinogradovi, S. caprimulga*	This study
H751_Al	GCTGGTGTGTAGTTGTCTGGGTCTCC	*S. vinogradovi*	This study
L70b	CCCATCYAACATCTCYGCATGATGAAACTT	*S. vinogradovi*	This study

L153b	AGCTATACAYTACACATCCGAYACCACAAC	*S. vinogradovi*	This study
H943	ACATTGGCTGATTGGTCGGAAGATTAT	*S. vinogradovi*	This study
H883b	AGGGCTAGGATTAGRATWGATAGGAC	*S. vinogradovi*	This study
H341	CAGTGAATAGAAGTAGGACTCCGATGT	*S. vinogradovi*	This study
L677	CTAAAGACGCCCTAGGATTCATACTC	*S. vinogradovi*	This study
H328a	GTAGTACTCCGATGTTTCAGGTTTC	*S. caprimulga*	This study
L710a	TCCTCCTTATATTGGTCCTATTTTCC	*S. caprimulga*	This study
L0a_pyg	CATCGTTGTWAATTCAACTACAAAAACCTA	*Pygeretmus spp.*	This study
H0a_pyg	GACCAGGGTAATWTTTATACTACAAAG	*Pygeretmus spp.*	This study
L537a_pyg (internal)	CGCCTTTCACTTYATCCTMCCATTCATC	*Pygeretmus spp.*	This study
L468a_pyg (internal)	TCGGAACAACCYTAGTAGAATGAAT	*Pygeretmus spp.*	This study
H667b_pyg (internal)	TCCTAGGGCGTCYTTGATTGT	*Pygeretmus spp.*	This study
H758a_pyg (internal)	GGRTTAGCTGGTGTRTAGTTGTCTG	*Pygeretmus spp.*	This study
H711_plat (internal)	GGGAGAAGAGGACTAGGAGAAGGAGAAGA	*P. platyurus*	This study
H711_plat (internal)	GGGAGAAGAGGACTAGGAGAAGGAGAAGA	*P. platyurus*	This study
L682_plat (internal)	GCCTTAGGATTCCTATTCCTTATTCTT	*P. platyurus*	This study
H828_plat (internal)	TGATCGGAGGATAGCGTAGGCG	*P. platyurus*	This study
L696_plat (internal)	CCTATTCCTTATTCTTCTTCTCCTTCTC	*P. platyurus*	This study

L236_Am2	TGACTRATCCGATAYCTACACGCCAAC	*Orientallactaga sibirica, Allactaga major*	This study
H479_Am3	GAARCCYCCTCAGATTCAYTCTACTA	*O. sibirica, A. major*	This study
L530_Am1	GCCTTCCATTTYATCCTRCCATTCAT	*O. sibirica, A. major*	This study
H932_Am1	GCGGAAGATYATGCTTCGTTGTT	*O. sibirica, A. major*	This study
L430_Am1	CCGTTATYACYAACCTCCTATCAGC	*Allactaga major*	This study
H751_Am2	CTGGTGTRTAGTTGTCTGGGTCTC	*Allactaga major*	This study

Phylogenetic reconstructions

Nuclear genes: combined data

Maximum parsimony reconstructions were performed in PAUP* 4.0b10[28]. The dataset included an additional matrix of binary characters corresponding to parsimony-informative indels. To reconstruct the optimum trees, heuristic searches were run with the starting tree obtained via stepwise addition, with random addition of sequences and 20 replicates. Tree-bisection-reconnection was used as the branch-swapping algorithm. To assess clade stability, 1000 bootstrap pseudoreplicates were analysed using the same options as above, but with 50 random addition replicates and 'Multrees' not invoked. Maximum likelihood (ML) reconstructions were conducted in IQTREE version 1.6[29]. The ModelFinder routine[30] was used to determine the optimum partitioning strategy and best-fit substitution models for each subset under the BIC criterion. At the initial stage, all gene × codon position combinations were treated as independent subsets. Both edge-linked proportional and edge-unlinked partition models were tested. BIC value was lower for the former; therefore, all subsequent analyses were performed assuming proportional branch lengths among subsets (for more details on partitioning, see Supplementary Table S5). Branch support was evaluated using Ultrafast Bootstrap[31] with 10,000 replicates and branch-wise approximate likelihood ratio test (SH-aLRT[32]) with 100,000 replicates.

Bayesian analysis of the nuclear concatenated data was performed in MrBayes 3.2[33] using the same partitioning scheme as in the ML analysis. Substitution models were specified based on the results of ModelFinder, except that all models with more than two rate parameters were set as GTR. Compound Dirichlet priors for branch lengths combined with a gamma prior on the tree length were invoked. All parameters except branch lengths were unlinked across partitions. The analysis included two independent runs of four chains with the default heating scheme. Chain length was set at five million generations with sampling every 2,000 generations. The Tracer 1.7 software[34] was used to check for convergence and determine the necessary burn-in fraction, which was 10% of the chain length. With these settings, the effective sample size exceeded 200 for all estimated parameters.

Individual gene analysis

The ML and Bayesian trees were generated for each individual nuclear gene using IQTREE and MrBayes, correspondingly. For both analyses, partitions were allocated and best-fit models were determined using ModelFinder as described above. In the ML analysis, branch support was estimated using Ultrafast Bootstrap with 10,000 replicates. The Bayesian analysis was run with the chain length of two million generations and sampling frequency of 1,000.

Supplementary Table S5. Partitioning and models.

The ModelFinder procedure under edge-linked proportional model partitioned the concatenated nuclear data into 10 subsets varying in substitution rate, rate heterogeneity, and base composition. Four subsets (shown in bold) consisted of the 3rd codon positions and intronic sequences, while the other six included only 1st and 2nd codon positions.

designation	genes / codon positions	legth - bp	best-fit model	relative rate	GC content - %
S12	a2ab_1 cnr1_1 lcat_1 lcat_2	755	TNe+I	0.11	52.7
VS2	a2ab_2 cnr1_2 acp5_2	558	F81+F	0.08	37.8
**F3**	a2ab_3 cmyc_3 cnr1_3	650	HKY+F+G4	1.82	76.4
F12	apob_1 brca1_1 brca1_2 brca2_1 brca2_2	1488	TPM2+F+G4	1.31	44.8
M2	apob_2 atp7_2 ghr_2 irbp_2 rag1_2 rag2_2 vwf_2	2035	TPM2u+F+I	0.44	39.9
**MF3**	apob_3 atp7_3 brca1_3 brca2_3 ghr_3 rag2_3 acp5_3	1788	K3Pu+F+G4	1.49	38.4
M12	atp7_1 cmyc_2 rag2_1 crem	1044	HKY+F+G4	0.48	45.5
M1	cmyc_1 ghr_1 irbp_1 rag1_1 vwf_1 acp5_1	1427	TIM+F+G4	0.46	59.6
**VF3**	irbp_3 vwf_3	532	K3Pu+F+G4	3.86	74.7
**MS3**	lcat_3 rag1_3 acp5_intron	716	K2P+I	1.33	54.2

Mitochondrial data: tree reconstructions and distance estimation

The *cytb* alignment was partitioned by codon position in both ML and Bayesian analyses; the best-fit model selection and support evaluation followed the same routine as with the nuclear genes. We generated the ultrametric tree by invoking a birth-death model and assuming a relaxed lognormal clock model with the mean rate fixed at unity. The analysis in BEAST 1,10 was run for 100 million generations, and parameters were logged to a file at every 50,000th step. Convergence and mixing were analyzed via Tracer 1.7.

To test for deviation from base composition homogeneity, the substitution pattern homogeneity test[35] as implemented in Mega 4[36] was performed based on the 3rd codon positions. The results demonstrated significant difference in base frequencies both among jerboa subfamilies and between some allactagine species (see Supplementary information below, Table S6, Fig. S1); we thus performed a second round of reconstructions (both ML and Bayesian) based on the alignment containing 3rd codon positions recoded into purines and pyrimidines.3

Species tree reconstructions

Species tree reconstructions employing a Bayesian coalescent framework were performed in *BEAST ver 1.8.4[37]. Forty lineages and sublineages of Allactaginae and five outgroups were used as operational taxonomic units (OTUs). Most of these OTUs corresponded to entities identified by the ABGD species delimitation analysis. Units with absent nuclear data were not included. In a few cases, entities with wide distribution range were split into two or more OTUs (e.g. *Pygeretmus pumilio* from Mongolia and Uzbekistan, *Scarturus elater* from N Caucasus and E Kazakhstan).

Two variants of the analysis were performed, differing by the inclusion of the *cytb* alignment. Prior to the analysis, the genotype data on each of the 15 nuclear genes were phased using PHASE software[38] via DNAsp ver.5[39]. In order to accelerate calculations, the number of sequences in the *cytb* alignment was reduced to 280 excluding most of the highly similar sequences of *O. sibirica* and *S. williamsi* (randomly selected among

haplotypes). The *cytb* data for *Scarturus elater zaisanicus* were excluded from the analysis because nuclear and mitochondrial data support conflicting positions for this taxon in relation to *S. e. elater* and *S. e. dzungariae*.

Each individual dataset was partitioned into one to three subsets following the results of ModelFinder analyses of the separate gene alignments. Substitution models were linked across partitions as in the ML analysis. Following the results of the LRT tests, either strict or relaxed lognormal gene-specific clock models were employed. No calibration information was used, and clock rate for *A2AB* was fixed at unity. Trial runs with default priors showed that the chains do not always reach a stationary state even at 500 million generations in length since the algorithm could get 'stuck' in a local optimum. To facilitate convergence, we had to use more informative priors. Prior densities for clock rates or UCLD mean rates of nuclear genes except *A2AB* were modeled using lognormal distributions with means and variances estimated from the posterior distributions produced by the additional BEAST analysis of the concatenated data, which employed the same partitioning scheme as in the species tree analysis. A diffuse uniform distribution bounded by 20 and 300 was applied as the prior for the *cytb* UCLD mean rate. We used non-random starting trees (the species tree and the gene trees) that were constructed based on the ultrametric tree obtained in the concatenated analysis. A birth-death prior for the species tree shape was assumed. We applied the piecewise-linear with constant root population size model with the "species.popMean" parameter set at 0.0001. Default priors were used for all other parameters. Four runs of 500 million generations each were conducted saving every 100,000th generation. Parameter convergence was assessed in Tracer 1.7 software[34]. Depending on the convergence of the MCMC trace, 40 to 60% of the chain length was discarded as the burnin. In the combined posterior sample, the effective sample size for all parameters except several "speciesTree.splitPopSize" parameters exceeded 100.

As the second method to infer the species tree, we used ASTRAL ver. 5.7.7[40]. The mulilocus bootstrap procedure was employed to account for gene tree estimation error ("-b" option, 1000 replicates). The input consisted of 16 sets of gene trees (3,600 trees each) extracted from the posterior samples generated by MrBayes. The tree sample for *cytb* was reconstructed in a separate analysis in MrBayes based on a reduced sample excluding

specimens with absent nuclear data. A second analysis employed gene bootstrapping ("--gene-only" option) based on the ML gene trees reconstructed by IQTREE.

Species delimitation

Automatic identification of the 'barcode gap' was performed using the online ABGD application (http://www.abi.snv.jussieu.fr/public/abgd/abgdweb.html) under the default set of parameters using the matrix of uncorrected *p*-distances as input. The prior for maximum intraspecific diversity was set to values <1%. Single threshold GMYC analysis was performed employing the GMYC web server (http://species.h-its. org/gmyc/[41] with the Maximum Clade Credibility tree produced by BEAST. The mPTP analysis was conducted based on the ML tree using the online version of the mPTP program (https://mptp.h-its.org/#/tree). In all analyses the outgroups were excluded.

Variation in base composition of cytb

Pattern homogeneity tests[35] were performed with 1000 replicates. Only sequences with more than 900 bp were used. *P*-values for interspecific comparisons are given in **Table S6.** To visualize variation in the base composition, Principle Component Analysis (PCA) of nucleotide frequencies was conducted in Statistica 7.0 (Statsoft, Inc. 1984-2007). The results are presented in **Fig S1**. The first axis (56.27% of total variation) is positively correlated with C content, the second axis (40.54% of total variation) corresponds to the difference in A and T content. The results demonstrated significant departure from homogeneity both within Allactaginae and among subfamilies. Among outgroups, Cardiocraniinae were the most distant from allactagines ($P<0.001$ for most comparisons). Within Allactaginae, two groups with deviant base composition were revealed. Sequences of *Allactaga severtzovi* were C-rich and differed significantly from *Scarturus, Pygeretmus*, and all outgroups. In contrast to that, sequences of *Scarturus aulacotis* and *S. euphraticus* were T-rich and differed significantly from the majority of allactagine species. Within species, significant differences were extremely rare.

Figure S2. Projection of cases onto the plane of the first two principle components. Dotted lines correspond to projections of the input variables (base frequencies).

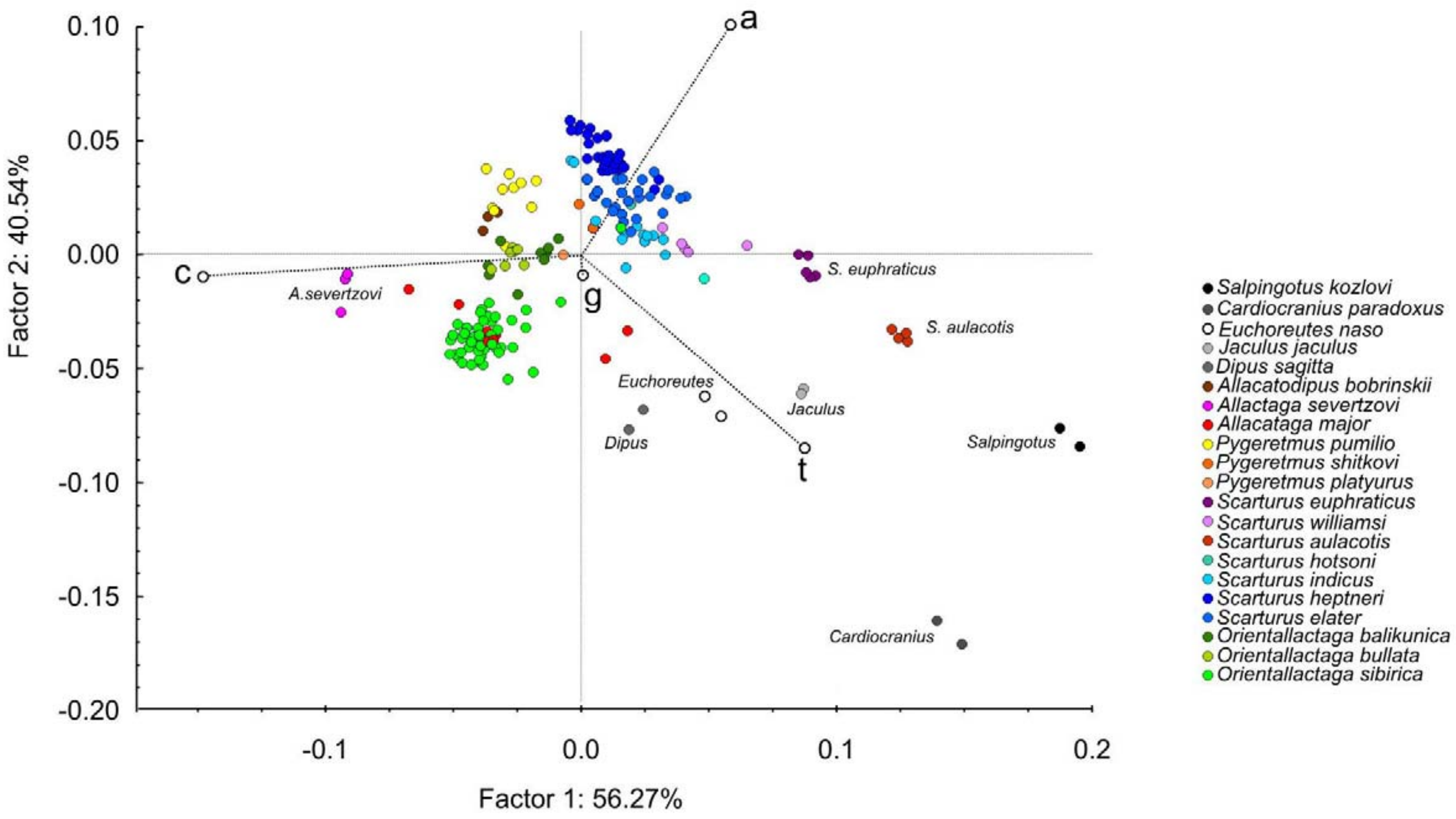

Table S6. *P*-values for Pattern Homogeneity tests.

Initially, pairwise tests were performed for 229 sequencies of 17 allactagin species and five outgroups. The table contains median *P*-values calculated across all pairwise tests for a given pair of species. Values below 0.01 and 0.05 are marked with red and yellow, respectively.

	Cardio cranius parado xus	Salpin gotus kozlov i	Euchor eutes naso	Jaculus jaculus	Dipus sagitta	Allacat odipus bobrin skii	Allacta ga severtz ovi	Allacat aga major	Pygere tmus pumili o	Pygere tmus shitkov i	Pygere tmus platyur us	Scartur us aulacot is	Scartur us euphra ticus	Scartur us willia msi	Scartur us aff.cap rimulg a	Scartur us hotsoni	Scartur us indicus	Scartur us heptne ri	Scartur us elater	Orient allacta ga baliku nica	Orient allacta ga bullata	Orient allacta ga sibirica
Cardiocranius paradoxus	1.0000																					
Salpingotus kozlovi	0.0085	1.0000																				
Euchoreutes naso	0.0010	0.0000	0.3720																			
Jaculus jaculus	0.0020	0.0135	0.6780	1.0000																		
Dipus sagitta	0.0000	0.0000	1.0000	0.1200	0.3260																	
Allacatodipus bobrinskii	0.0000	0.0000	0.0030	0.0000	0.0085	1.0000																
Allactaga severtzovi	0.0000	0.0000	0.0000	0.0000	0.0000	0.0920	1.0000															
Allacataga major	0.0000	0.0000	0.0255	0.0000	0.0975	0.1260	0.0670	0.0915														
Pygeretmus pumilio	0.0000	0.0000	0.0020	0.0000	0.0050	1.0000	0.0440	0.0700	1.0000													
Pygeretmus shitkovi	0.0000	0.0000	0.0145	0.0030	0.0320	1.0000	0.0070	0.1135	1.0000	1.0000												
Pygeretmus platyurus	0.0000	0.0000	0.0220	0.0045	0.0620	1.0000	0.0300	0.2250	1.0000	1.0000												
Scarturus aulacotis	0.0000	0.0025	0.0680	0.6805	0.0250	0.0010	0.0000	0.0000	0.0020	0.0355	0.0100	1.0000										
Scarturus euphraticus	0.0015	0.0555	0.0780	0.3455	0.0060	0.0000	0.0000	0.0000	0.0000	0.0000	0.0005	0.2540	1.0000									
Scarturus williamsi	0.0000	0.0000	0.0930	0.0575	0.0655	0.0540	0.0000	0.0140	0.0605	1.0000	0.1840	0.2385	0.0130	1.0000								
Scarturus aff.caprimulga	0.0000	0.0000	0.2310	0.1670	0.1570	0.0270	0.0000	0.0260	0.0195	0.2325	0.1110	0.2730	0.0315	1.0000								
Scarturus hotsoni	0.0000	0.0000	0.0325	0.0090	0.0185	0.2290	0.0020	0.0490	0.3115	1.0000	1.0000	0.0960	0.0010	1.0000	1.0000							
Scarturus indicus	0.0000	0.0000	0.0690	0.0140	0.0255	0.1690	0.0040	0.0410	0.2255	1.0000	0.3150	0.0555	0.0015	1.0000	1.0000	1.0000	1.0000					
Scarturus heptneri	0.0000	0.0000	0.0040	0.0000	0.0025	0.1850	0.0010	0.0180	0.2950	1.0000	0.2380	0.0170	0.0000	0.3100	0.0790	1.0000	1.0000	1.0000				
Scarturus elater	0.0000	0.0000	0.0220	0.0050	0.0140	0.2530	0.0030	0.0380	0.2785	1.0000	1.0000	0.0420	0.0010	1.0000	0.2450	1.0000	1.0000	1.0000	1.0000			
Orientallactaga balikunica	0.0000	0.0000	0.0225	0.0020	0.0365	1.0000	0.0410	0.4040	1.0000	1.0000	1.0000	0.0030	0.0000	0.1340	0.1200	1.0000	0.3275	0.1640	0.3280	1.0000		
Orientallactaga bullata	0.0000	0.0000	0.0145	0.0010	0.0200	1.0000	0.0690	1.0000	1.0000	1.0000	1.0000	0.0030	0.0000	0.1310	0.0490	0.3080	0.3440	0.1710	0.3330	1.0000	1.0000	
Orientallactaga sibirica	0.0000	0.0000	0.0285	0.0020	0.1290	0.3550	0.1310	1.0000	0.1600	0.1775	1.0000	0.0030	0.0000	0.0340	0.0220	0.1050	0.0960	0.0250	0.0830	1.0000	1.0000	1.0000

Molecular dating

The analyses in BEAST 1.10 were performed using the partitioning scheme and substitution models as inferred via a ModelFinder procedure. The birth-death model was employed as the tree shape prior. Chain length was 100 million generations, and parameters were logged to a file at every 50,000th step. Convergence diagnostics were performed via Tracer 1.7[34], the burn-in fraction was set to 10%, and the effective sample size exceeded 1,000 for all parameters. The maximum clade credibility tree was generated by Tree Annotator 1.10.4 (available in BEAST).

The method of estimation of prior calibration density parameters was executed according to the following steps.

As the first step, the analysis in BEAST was run without calibrations to produce relative split ages (RSA). The UCLD mean rate for the F12 subset was set at 1.0. Diffuse uniform priors (0.0–10.0) were used for the mean rate parameters of all other subsets.

Second, using the RSA produced by BEAST, the empirical scaling factor (S = FAD/RSA) was calculated for each calibration node, and the node with the largest S (S_{max}) was identified (see Marshall[42] for details). In this calculation, FAD estimates corresponded to centres of stratigraphic units. We discarded three FADs (*A.major, O. sibirica, S, cf.williamsi*) that were evidently too young to be informative for calibration (i.e. if the age of the oldest fossil is less than one-half of the expected divergence time of the taxon they represent). In our case, S_{max} corresponded to the split between Allactaginae and Euchoreutinae.

Third, according to Solow[43] and Marshall[42,44], the true age of the node with S_{max} can be estimated as $Td_{max} = RSA_{max} \times (S_{max} + \Delta)$, where Δ is a correction term calculated as the average gap size between successive S values. The latter estimate is unbiased if S values follow a uniform distribution. Although the exact form of the distribution was unknown, no marked departure from uniform density was evident in our data.

Next, based on the estimated Td_{max}, the ultrametric tree was rescaled, and ages of all calibration nodes were produced, which, in turn, allowed calculation of all absolute and relative ghost lineage times.

Finally, the observed mean (M) and standard deviation (σ) of inferred relative ghost lineage times were used to determine the parameters of calibration densities for each node. The latter were modelled using gamma distribution with ($M \times FAD + W/2$) as mean, ($(\sigma \times FAD)^2 + W^2/12$) as variance, and FAD_{min} as offset. Additional components of mean and variance were introduced to account for the dating error with W being the time span of the stratigraphic zone.

Supplementary Table S7. The results of clock tests.

Hierarchical likelihood ratio tests were performed using the ML topology obtained from the concatenated data. All protein-coding genes were partitioned by codon position. GTR+G model was applied to all subsets; rate parameters, base frequencies, substitution rates, and alpha parameters of gamma distributions were unlinked. Degrees of freedom of the chi-square tests were adjusted for the number of identical sequences. Taking into account that low information content in short and slow evolving genes may lead to erroneous acceptance of the strict clock model we used a more liberal rejection criterion *P*=<0.1.

**Gene**	**P**	**strict clock**
A2AB	0.12	**accepted**
APOB	0.00	rejected
ATP7	0.00	rejected
BRCA1	0.01	rejected
BRCA2	0.00	rejected
CMYC	0.00	rejected
CNR1	0.17	**accepted**
GHR	0.01	rejected
IRBP	0.00	rejected
LCAT	0.17	**accepted**
RAG1	0.10	rejected
RAG2	0.00	rejected
VWF	0.00	rejected
CREM	0.06	rejected
ACP5	0.10	rejected

Table S8. Calibrations used for the molecular clock analysis in BEAST. Ages (Mya) are given according to NOW Database (https://nowdatabase.org/).

node	Fossil and its interpretation	Age	Reference	Calibration density			
				offset	mean	std.dev	95% upper bound
Dipus / *Jaculus*	earliest *Dipus* – *Dipus sp.* in Shala formation, middle Baodean, China,	~8.2	[45]	8.2	10.73	1.365	14.02
Cardiocranius / *Salpingotus*	earliest representatives of *Salpingotus* lineage – *S. primitivus*, Bahe formation, China	10-12.5	[46]	10.0	13.65	1.780	17.87
Allactaginae / Dipodinae or Euchoreutinae	earliest Allactaginae - *Protalactaga. shevyrevae*, upper part of the Lower Miocene, Akzhar Formation, Zaisan, Kazakhstan	16.0–20.0	[47]	16.0	22.22	3.096	29.59
Allactaga major / *A. severtzovi*	*Allactaga ukrainica* - a species closely related to *A. severtzovi* from Early Pleistocene, S Ukraine	1.72–1.9	NOW Database https://nowdatabase.org/	1.72	2.30	0.295	3.00

Pygeretmus / *Scarturus*	earliest *Pygeretmus* – Late Pliocene, Kiikbay, SE Kazakhstan	3.0–3.28	[48]	3.0	3.99	0.511	5.21
Pygeretmus ss /*Alactagulus*	earliest *Alactagulus Alactagulus* indet - Early Pleistocene, Orkhon, Mongolia	2.18–2.45	[49]	2.18	2.94	0.380	3.84
Scarturus elater species group / sister lineages	earliest member of *S. elater* species group – *Allactaga elater* - Early Pleistocene, Akkulaevo, Bashkiria, Russia	2.2–2.5	[50]	2.20	2.98	0.387	3.90

Species distribution modeling (SDM).

The occurrence records of Allactaginae used in the species distribution modeling (SDM) were obtained from our field observations, ZMMU collections, other collections accessed via the Global Biodiversity Information Facility (GBIF, https://www.gbif.org), and literature sources. The environmental data used for SDM were 30 arc-second grids (approximately 1 km resolution) of climate. Current climate data (BioClim 1-19) were obtained from WORLDCLIM Version 2.0[43]; paleo-climate data for intervals 20–5,400 kya were obtained from Oscillayers dataset[44]. Paleo-climate data originally available at 2.5 arc-minutes were upscaled to 30 arc-second.

The extent of the study area or of the "landscape of interest" significantly affects SDM results[46,47]. To define the study area of a species, we clipped environmental variables using rectangle mask covering the area of occurrence points plus 3 degrees in each direction. The model for current environments was constructed with default MAXENT settings, as these were demonstrated to be the most appropriate for wide-ranging data[48]. We used the MAXENT logistic output, which provides estimates of relative habitat suitability[51]. To estimate the model's performance, we used the area under the Receiver Operating Characteristic curve (AUC) test, which is an extensively used measure in species distribution modeling[52]. Since most bioclimatic variables are intercorrelated we aimed to avoid overprediction and therefore calculated the matrix of pair-wise correlations of 19 bio-climatic variables for each study area using “Band Collection Statistics” tool from the standard Arc Toolbox, Spatial Analyst Tools (ArcGIS Desktop 10.8.1). We chose a pair of variables with the highest value of correlation coefficients, built two models each with one of these variables removed, estimated the model’s performance using the AUC test, and excluded the variable associated with a lower AUC value. This procedure was repeated with the next pairs of highly correlated variables and was stopped when removal of a variable from the model resulted in an abrupt decline of the AUC value. Models with reduced sets of environmental variables for current time were projected onto the paleo-environmental data sets. For each node, we used divergence time intervals estimated from molecular dating with 50 kyr added to maximal and minimal values. Within these time intervals, we got the set of models of relative habitat suitability (RHS) for each 10 kyr . To define the areas of real species occurrence,

the original model values, ranging continuously from 0 to 1, were transformed to a binary 0 or 1 using a threshold value. The threshold value was chosen to be equal to the "maximum training sensitivity plus specificity"; this threshold was experimentally demonstrated to provide optimal results[49]. Within each of the analyzed time periods estimated by genetic analysis as the time of branching of genetic lineages, SDM revealed episodes of ancestor's geographic range fragmentations that can be interpreted as isolation leading to speciation. This result can validate the use of the chosen approach.

Figure S3. Bayesian phylogeny of Allactaginae based on the concatenated alignment of nuclear genes. Values above the branches correspond to the posterior probabilities in Bayesian and bootstrap support in the ML and MP analyses, respectively (BI/ML/MP).

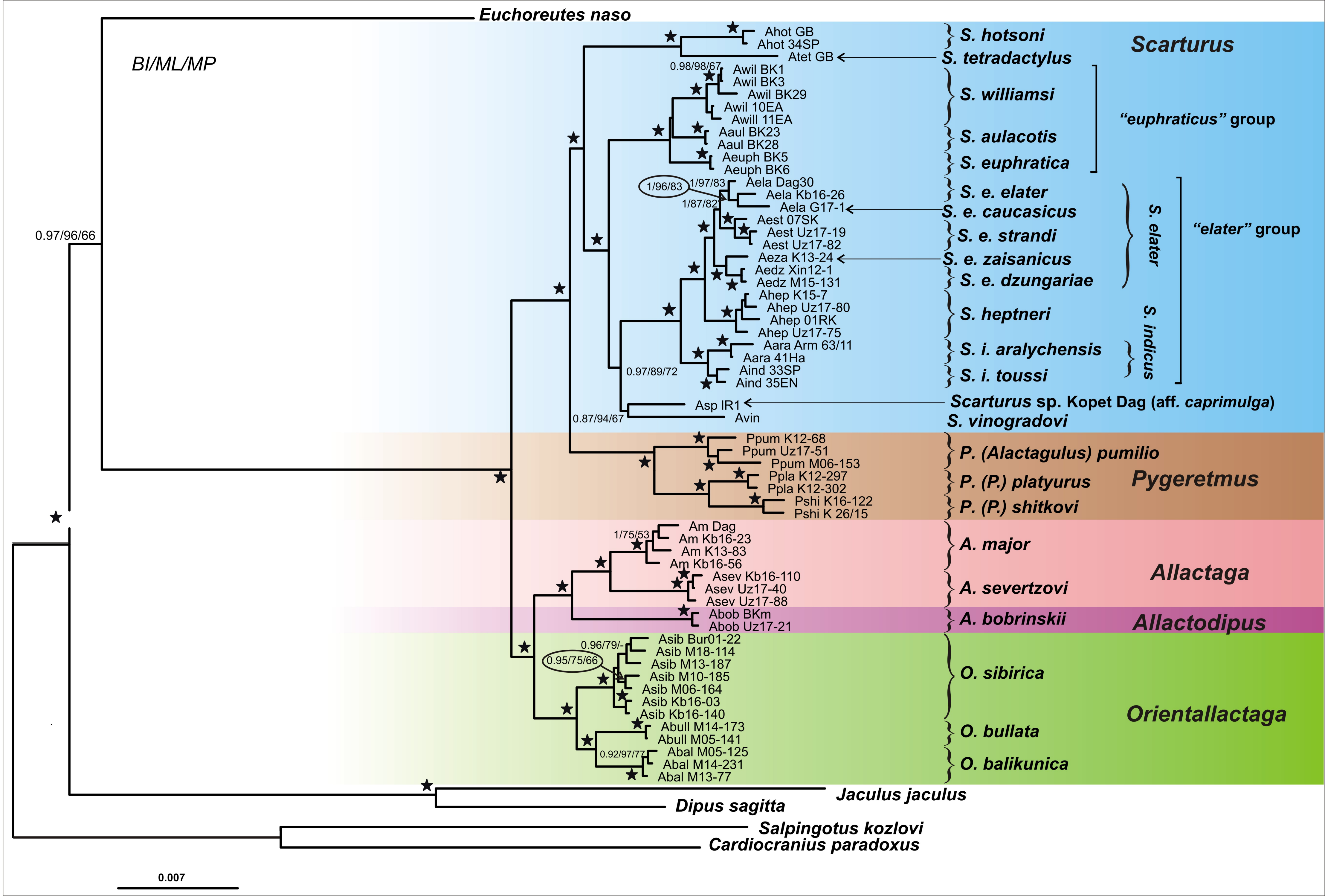

Euchoreutes naso
BI/ML/MP
Ahot GB
Ahot 34SP
Atet GB
S. hotsoni
S. tetradactylus
Scarturus
0.98/98/67
Awil BK1
Awil BK3
Awil BK29
Awil 10EA
Awill 11EA
Aaul BK23
Aaul BK28
Aeuph BK5
Aeuph BK6
S. williamsi
S. aulacotis
S. euphratica
"euphraticus" group
1/96/83
1/97/83
1/87/82
Aela Dag30
Aela Kb16-26
Aela G17-1
Aest 07SK
Aest Uz17-19
Aest Uz17-82
Aeza K13-24
Aedz Xin12-1
Aedz M15-131
S. e. elater
S. e. caucasicus
S. e. strandi
S. e. zaisanicus
S. e. dzungariae
S. elater
"elater" group
Ahep K15-7
Ahep Uz17-80
Ahep 01RK
Ahep Uz17-75
Aara Arm 63/11
Aara 41Ha
Aind 33SP
Aind 35EN
S. heptneri
S. i. aralychensis
S. i. toussi
S. indicus
0.97/96/66
0.97/89/72
Asp IR1
Avin
0.87/94/67
Scarturus sp. Kopet Dag (aff. caprimulga)
S. vinogradovi
Ppum K12-68
Ppum Uz17-51
Ppum M06-153
Ppla K12-297
Ppla K12-302
Pshi K16-122
Pshi K 26/15
P. (Alactagulus) pumilio
P. (P.) platyurus
P. (P.) shitkovi
Pygeretmus
1/75/53
Am Dag
Am Kb16-23
Am K13-83
Am Kb16-56
Asev Kb16-110
Asev Uz17-40
Asev Uz17-88
A. major
A. severtzovi
Allactaga
Abob BKm
Abob Uz17-21
A. bobrinskii
Allactodipus
0.96/79/-
0.95/75/66
Asib Bur01-22
Asib M18-114
Asib M13-187
Asib M10-185
Asib M06-164
Asib Kb16-03
Asib Kb16-140
Abull M14-173
Abull M05-141
0.92/97/77
Abal M05-125
Abal M14-231
Abal M13-77
O. sibirica
O. bullata
O. balikunica
Orientallactaga
Jaculus jaculus
Dipus sagitta
Salpingotus kozlovi
Cardiocranius paradoxus
0.007

Figure S4. Maximum likelihood phylogeny of the Allactaginae based on the *cytb* gene sequences. Values above the branches correspond to the posterior probabilities and bootstrap support (1,000 pseudoreplicates) in the BI and ML analyses, respectively. The results of ABGD species delimitation analysis are shown to the right.

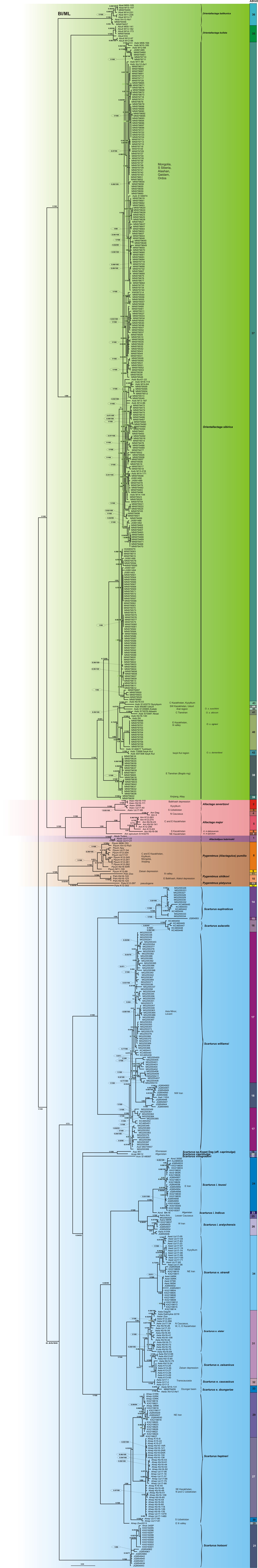

BI/ML
ABCD
Orientallactaga balikunica
Orientallactaga bullata
Mongolia, S Siberia, Alashan, Qaidam, Ordos
Orientallactaga sibirica
O. s. suschkini
O. s. altorum
O. s. ognevi
O. s. dementiewi
Allactaga severtzovi
Allactaga major
A. m.djetysuensis
A. m.spiculum
Allactodipus bobrinskii
Pygeretmus (Alactagulus) pumilio
Pygeretmus shitkovi
Pygeretmus platyurus
Scarturus euphraticus
Scarturus aulacotis
Scarturus williamsi
NW Iran
Scarturus sp.Kopet Dag (aff. caprimulga)
Scarturus caprimulga
Scarturus vinogradovi
Scarturus i. toussi
E Iran
Scarturus i. Indicus
Afganistan
Lesser Caucasus
Scarturus i. aralychensis
W Iran
Kyzylkum
Scarturus e. strandi
NE Iran
N Caucasus, W, C, E Kazakhstan
Scarturus e. elater
Scarturus e. zaisanicus
Zaisan depression
Scarturus e. caucasicus
Transcaucasia
Scarturus e. dzungariae
Dzungar basin
Scarturus heptneri
SE Kazakhstan, N and C Uzbekistan
S Uzbekistan
E Ili valley
Scarturus hotsoni

Figure S5. Species tree of Allactaginae produced by the *BEAST algorithm using the Bayesian multispecies coalescent approach (**A**) and derived from ASTRAL algorithm (**B**). In Fig. S4A, numbers at nodes denote posterior probabilities in *BEAST. In Fig. S4B, numbers at nodes correspond to consensus scores obtained using posterior samples from MrBayes (left) and with gene bootstrapping based on ML gene trees (right).

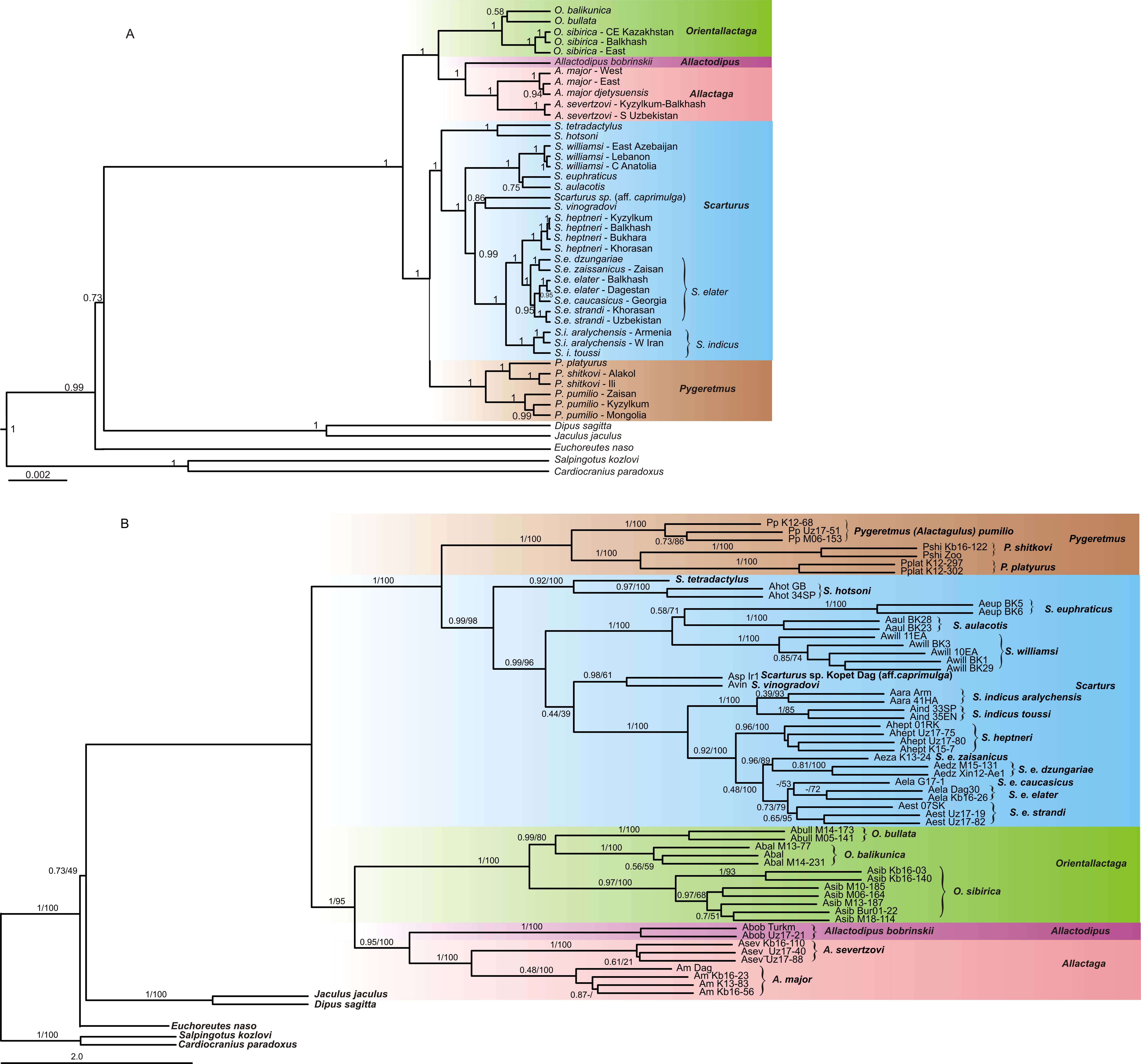

A
O. balikunica
O. bullata
O. sibirica - CE Kazakhstan
O. sibirica - Balkhash
O. sibirica - East
Orientallactaga
Allactodipus bobrinskii
Allactodipus
A. major - West
A. major - East
A. major djetysuensis
Allactaga
A. severtzovi - Kyzylkum-Balkhash
A. severtzovi - S Uzbekistan
S. tetradactylus
S. hotsoni
S. williamsi - East Azebaijan
S. williamsi - Lebanon
S. williamsi - C Anatolia
S. euphraticus
S. aulacotis
Scarturus sp. (aff. caprimulga)
S. vinogradovi
Scarturus
S. heptneri - Kyzylkum
S. heptneri - Balkhash
S. heptneri - Bukhara
S. heptneri - Khorasan
S.e. dzungariae
S.e. zaissanicus - Zaisan
S.e. elater - Balkhash
S.e. elater - Dagestan
S.e. caucasicus - Georgia
S.e. strandi - Khorasan
S.e. strandi - Uzbekistan
S. elater
S.i. aralychensis - Armenia
S.i. aralychensis - W Iran
S. i. toussi
S. indicus
P. platyurus
P. shitkovi - Alakol
P. shitkovi - Ili
P. pumilio - Zaisan
P. pumilio - Kyzylkum
P. pumilio - Mongolia
Pygeretmus
Dipus sagitta
Jaculus jaculus
Euchoreutes naso
Salpingotus kozlovi
Cardiocranius paradoxus
0.002
B
Pp K12-68
Pp Uz17-51
Pp M06-153
Pygeretmus (Alactagulus) pumilio
Pshi Kb16-122
Pshi Zoo
P. shitkovi
Pplat K12-297
Pplat K12-302
P. platyurus
Pygeretmus
S. tetradactylus
Ahot GB
Ahot 34SP
S. hotsoni
Aeup BK5
Aeup BK6
S. euphraticus
Aaul BK28
Aaul BK23
S. aulacotis
Awill 11EA
Awill BK3
Awill 10EA
Awill BK1
Awill BK29
S. williamsi
Asp Ir1 Scarturus sp. Kopet Dag (aff.caprimulga)
Avin S. vinogradovi
Scarturs
Aara Arm
Aara 41HA
S. indicus aralychensis
Aind 33SP
Aind 35EN
S. indicus toussi
Ahept 01RK
Ahept Uz17-75
Ahept Uz17-80
Ahept K15-7
S. heptneri
Aeza K13-24 S. e. zaisanicus
Aedz M15-131
Aedz Xin12-Ae1
S. e. dzungariae
Aela G17-1
S. e. caucasicus
Aela Dag30
Aela Kb16-26
S. e. elater
Aest 07SK
Aest Uz17-19
Aest Uz17-82
S. e. strandi
Abull M14-173
Abull M05-141
O. bullata
Abal M13-77
Abal
Abal M14-231
O. balikunica
Orientallactaga
Asib Kb16-03
Asib Kb16-140
Asib M10-185
Asib M06-164
Asib M13-187
Asib Bur01-22
Asib M18-114
O. sibirica
Abob Turkm
Abob Uz17-21
Allactodipus bobrinskii
Allactodipus
Asev Kb16-110
Asev Uz17-40
Asev Uz17-88
A. severtzovi
Allactaga
Am Dag
Am Kb16-23
Am K13-83
Am Kb16-56
A. major
Jaculus jaculus
Dipus sagitta
Euchoreutes naso
Salpingotus kozlovi
Cardiocranius paradoxus
2.0

Figure S6. Plot of *cytb* sequence divergence (Y-axis) against node ages inferred from nuclear data (X-axis). Both X and Y values were obtained via corresponding analyses in BEAST 1.10. The red line shows the non-linear regression curve obtained with the use of non-linear estimation module in Statistica 7.0. A polynomial model was used: $Y= aX+bX^2+c$. The following values of coefficients were obtained: a= 0.078842, b=-0.004852, c=0.010153. Both a and b are significantly different from zero (*P*=0.0000 and *P*=0.027, respectively); thus, indicating non-linear relationship between time and *cytb* divergence. This effect can be explained by the *cytb* sequence saturation that is not corrected for by the available substitution models. For the oldest split, the node height in the *cytb* tree is underestimated by more than 40%.

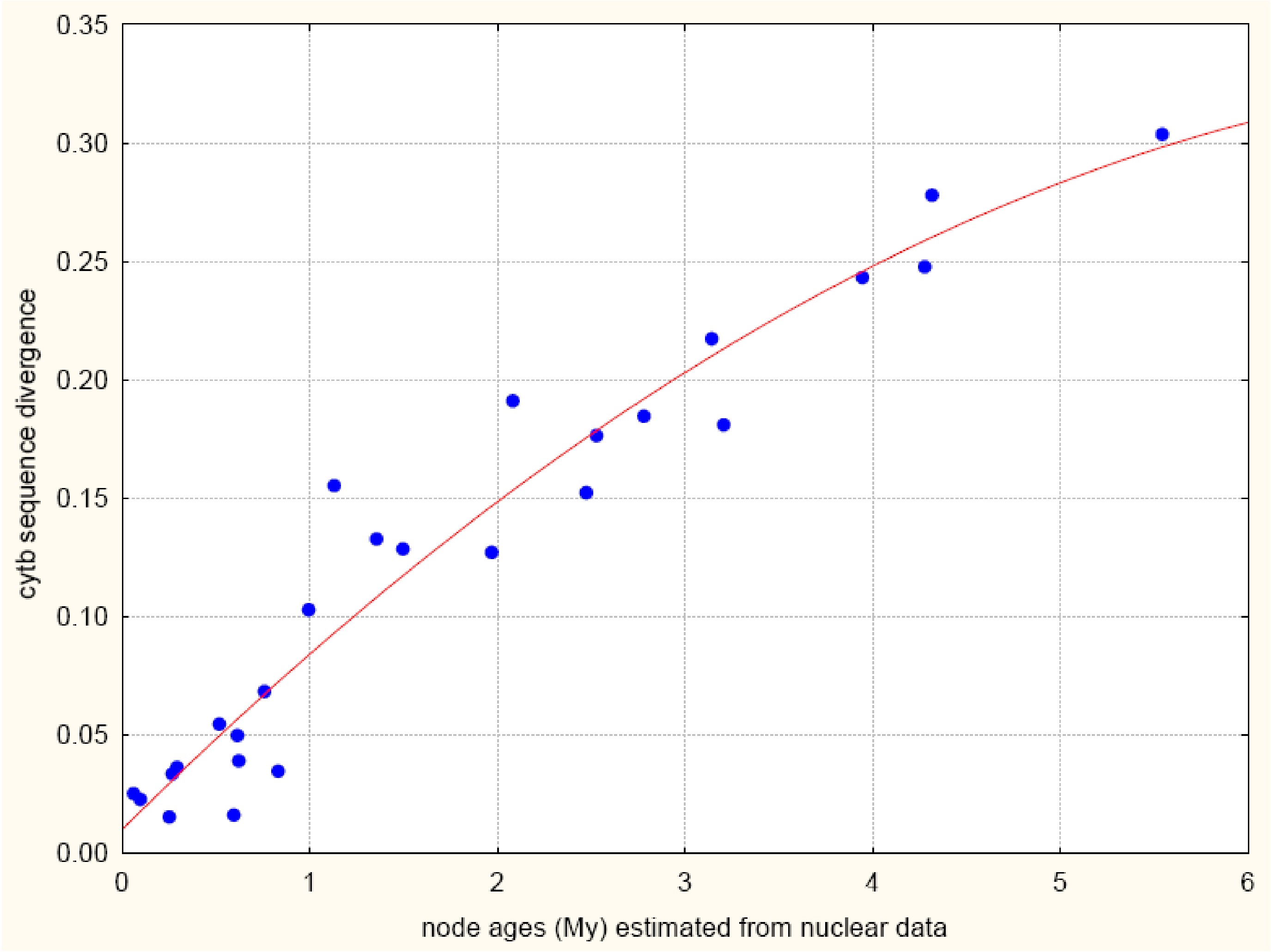

Supplementary information. ABGD results

Description of sequence groups as recognized by the ABGD procedures

1. Allactodipus bobrinskii
2. Allactaga severtzovi – N Uzbekistan, Balkhash depression
3. Allactaga severtzovi - S Uzbekistan
4. Allactaga major – N Caucasus
5. Allactaga major – C,E Kazakhstan
6. Allactaga major djetysuensis - SE Kazakhstan
7. Allactaga major spiculum – NE Kazakhstan – N of Irtysh
8. Pygeretmus pumilio - most of the range in W and E Central Asia
9. Pygeretmus pumilio - Zaisan depression
10. Pygeretmus shitkovi - E Balkhash depression
11. Pygeretmus shitkovi - E Ili valley
12. Pygeretmus platyurus
13. Pygeretmus platyurus putative pseudogene
14. Scarturus euphraticus - most of the range
15. Scarturus euphraticus - Iran
16. Scarturus aulacotis
17. Scarturus williamsi - most of the range
18. Scarturus williamsi - NW Iran
19. Scarturus aff. caprimulga - Kopet Dag
20. Scarturus caprimulga - Afganistan
21. Scarturus hotsoni
22. Scarturus vinogradovi
23. Scarturus indicus s.str. - Afganistan
24. Scarturus indicus toussi - Iran
25. Scarturus (i.) aralychensis - Lesser Caucasus
26. Scarturus (i.) aralychensis - Iran
27. Scarturus heptneri - SE Kazakhstan, N,C Uzbekistan
28. Scarturus heptneri - E Ili valley

29. Scarturus heptneri - NE Iran
30. Scarturus heptneri - S Uzbekistan
31. Scarturus elater sensu stricto - most of the range in SE Europe, N Caucasus, W,C,E Kazakhstan including Zaisan depression
32. Scarturus e. caucasicus - Transcaucasia
33. Scarturus (e.) dzungariae - Dzungar basin
34. Scarturus (e.) strandi - Uzbekistan, Turkmenistan, NE Iran
35. Orientallactaga bullata
36. Orientallactaga balikunica
37. Orientallactaga sibirica - E Central Asia
38. Orientallactaga sibirica - Bogdo mnts
39. Orientallactaga sibirica - Altay
40. Orientallactaga sibirica ognevi - E Kazakhstan
41. Orientallactaga sibirica altorum - C Tianshan
42. Orientallactaga sibirica dementiewi - Issyk Kul region
43. Orientallactaga sibirica - C Kazakhstan, Kyzylkum
44. Orientallactaga sibirica – SW Kazakhstan
45. Orientallactaga sibirica suschkini– Aral region

Table S9. Divergence times of Allactaginae as estimated by BEAST based on nuclear concatenation.

Tmrca	UCLD			RLC_poisson		
	median	95% HPD		median	95% HPD	
Dipodidae	**21.05**	18.95	23.38	**21.59**	19.58	23.49
Cardiocaniinae	**12.69**	10.85	14.69	**13.49**	11.94	15.09
Dipodinae + sister group	**18.96**	17.35	20.91	**19.27**	17.69	21.07
Dipus+*Jaculus*	**9.51**	8.49	10.75	**9.23**	8.35	10.14
Euchoreutes + Allactaginae	**17.94**	16.52	19.80	**18.13**	16.49	19.64
Allactaginae	**5.54**	4.88	6.29	**5.69**	5.09	6.36
Orientallactaga+*Allactaga*	**4.28**	3.64	4.99	**4.37**	3.78	4.92
Alactodipus +*Allactaga*	**3.21**	2.65	3.77	**3.28**	2.85	3.78
Allactaga	**1.97**	1.71	2.33	**2.01**	1.73	2.34
A. severtzovi	**0.06**	0.00	0.21	**0.06**	0.00	0.21
Orientallactaga	**2.08**	1.60	2.65	**2.17**	1.76	2.68
O. bullata + *O. balikunica*	**1.50**	1.08	1.95	**1.54**	1.19	1.97
O. sibirica	**0.52**	0.32	0.74	**0.55**	0.36	0.78
Pygeretmus + *Scarturus*	**4.32**	3.81	4.87	**4.48**	4.01	5.00
Pygeretmus	**2.53**	2.22	2.90	**2.57**	1.73	2.34
P. pumilio	**0.83**	0.57	1.14	**0.85**	0.58	1.12

P. pumilio except Zaisan	**0.60**	0.33	0.84	**0.60**	0.39	0.87
P. platyurus + *P. shitkovi*	**1.36**	1.05	1.70	**1.39**	1.10	1.69
P. shitkovi	**0.29**	0.11	0.51	**0.30**	0.12	0.50
Scarturus	**3.95**	3.46	4.48	**4.10**	3.65	4.67
S. hotsoni + *S. tetradactylus*	**1.84**	1.33	2.44	**1.90**	1.44	2.46
VECE clade (*Scarturus* without *S. hotsoni* & *S. tetradactylus*	**3.14**	2.75	3.61	**3.21**	2.84	3.69
S. euphraticus species group	**1.13**	0.84	1.45	**1.21**	0.93	1.49
S. williamsi	**0.27**	0.10	0.44	**0.29**	0.14	0.49
S. vinogradovi + *S. caprimulga* + *S. elater*	**2.78**	2.40	3.20	**2.85**	2.49	3.26
S. vinogradovi + *S. caprimulga*	**2.47**	1.89	3.00	**2.49**	1.89	2.95
S. elater species group	**1.50**	1.20	1.82	**1.51**	1.18	1.80
S. indicus	**0.61**	0.40	0.90	**0.64**	0.39	0.89
S.i.aralychensis	**0.10**	0.00	0.25	**0.10**	0.00	0.27
S. elater + *S. heptneri*	**0.99**	0.77	1.23	**1.01**	0.78	1.26
S. elater ss + *S. e. dzungariae*	**0.76**	0.58	0.97	**0.78**	0.58	0.99
S. elater ss + *S. e.strandi*	**0.62**	0.45	0.81	**0.64**	0.47	0.84
S. e.strandi	**0.25**	0.10	0.43	**0.26**	0.11	0.44

UCLD – relaxed uncorrelated lognormal clock model. RLC_poisson – Bayesian random local clocks with Poisson process for rate variation

All values are corrected for ancestral polymorphism using 0.15 My as the average allele coalescence time.

Table S10. Taxonomic changes in Allactaginae Vinogradov, 1925.

The names of taxa which status has changed compared to the latest revision are given in red. According to the taxonomy suggested in the current study *Allactaga (major) spiculum, Scarturus (elater) strandi, Scarturus (elater) dzungariae,* and *Scarturus (indicus) aralychensis* are regarded as semispecies (species in statu nascendi). Question marks indicate taxa which taxonomic status requires additional study. Names of valid genera are shown in bold. The name "Microallactaga" Shenbrot, 1974 is a nomen nudum. Species allocated to the genera *Allactaga*, *Orientallactaga*, and *Scarturus* are marked with colors (yellow, grey, and green, respectively).

Suggested taxonomy<br>5 genera, 19 species and 4 semispecies	Michaux & Shenbrot, 2017[53]<br>Lebedev et al., 2013[1]5 genera,<br>16 species	MSW3:<br>Holden, Musser, 2005[54]<br>3 genera, 16 species	Shenbrot et al., 1995[55]<br>3 genera, 13 species	Ognev, 1948[56]<br>4 genera, 10 species	Vinogradov, 1937[57]<br>4 genera, 12 species
Allactaga Cuvier, 1836<br>*A. major* (Kerr, 1792)<br>*A. m. major*<br>*A. m. djetysuensis* Shenbrot, 1991<br>*? A. m. ssp* (western part of the range)<br>***A. (m.) spiculum* (Lichtenstein, 1825)**<br>*A. severtzovi* Vinogradov, 1925<br>*A. s. severtzovi* (including *A. s. chorezmi*)<br>*? A. s. ssp* (S Uzbekistan)	***Allactaga***<br>*A. major*<br>*A. m. major*<br>*A. m. spiculum*<br>*A. m. djetysuensis*<br>*A. severtzovi*<br>*A. s. severtzovi*<br>*A. s. chorezmi* Shenbrot, 1993	***Allactaga***<br>subgenus *Allactaga*<br>*A.(A.) major*<br>*A.(A.) severtzovi*	***Allactaga***<br>subgenus *Allactaga*<br>*A.(A.) major*<br>*A. m. major*<br>*A.. m. djetysuensis*<br>*A. m. spiculum*<br>*A.(A.) severtzovi*<br>*A. s. severtzovi*<br>*A. s. chorezmi*	***Allactaga***<br>subgenus *Allactaga*<br>*A.(A.) jaculus* Pallas, 1778<br>(=*major*)<br>*A. j. jaculus*<br>*A. j. fuscus* Ognev, 1924<br>*A. j. decumanus* Lichtenstein, 1825<br>*A. j. intermedius* Ognev, 1948<br>*A. j. vexillarius* Eversmann, 1840<br>*A. j. spiculum*<br>*A.(A.) severtzovi*	***Allactaga***<br>*A. jaculus*<br>*A. severtzovi*
		A.(A.) elater<br>*A.(A.) vinogradovi*<br>*A.(A.) firouzi*<br>*A.(A.) hotsoni*	*A.(A.) elater*<br>*elater* subspecies group<br>*A. e. elater*<br>*A. e. caucasicus*<br>*A. e. dzungariae*<br>*A. e. zaisanicus*<br>*indica* subspecies group<br>*S. e. indica*<br>*A. e. turkmeni* Goodwin, 1940<br>*A. e. aralychensis*<br>*A.(A.) vinogradovi*<br>*A.(A.) hotsoni* (including *firouzi)*	*A.(A.) elater* Lichtenstein, 1825<br>*A. e. elater*<br>*A. e. strandi*<br>*A. e. aralychensis*<br>*A. e. caucasicus*<br>*A.e. kizljaricus* Satunin, 1907<br>*A. e. dzungariae*<br>*A. e. vinogradovi*	*A.. elater*<br>*A. e. elater*<br>*A.e. kizljaricus*<br>*A. e. aralychensis*<br>*A. e. caucasicus*<br>*A. e. strandi,*<br>*A. e. dzungariae*<br>*A. e. indica*
		subgenus *Paralactaga*<br>*Allactaga (P.) euphratica*<br>*A. (P.) williamsii*	subgenus *Paralactaga*<br>*Allactaga (P.) euphratica*<br>*A. e. euphratica*<br>*A. e. williamsi*<br>*A. e. caprimulga*	*A.(A.) williamsi*	*A. williamsi*<br>*A. euphratica*<br>*A. hotsoni*
Orientallactaga Shenbrot, 1984<br>*O. sibirica* (Forster, 1778)<br>*O. sibirica* semispecies or subspecies group ("eastern")<br>*O. s. sibirica*<br>*O. s. saltator* (Eversmann, 1848)<br>*O. s. annulata* (Milne-Edwards, 1867)<br>*O. s. longior* (G. S. Miller, 1911)<br>*O. s. bulganensis* (Shenbrot, 1991)<br>*? O. s. ssp* (see Cheng et al., 2020)<br>*O. suschkini* semispecies or subspecies group ("western")<br>*O. s. suschkini* (Satunin, 1900)<br>*? O. s. rueckbeili* (Thomas, 1914)<br>*O. s. altorum* (Ognev, 1946)<br>*O. s. dementiewi* (Toktosunov, 1958)<br>*O. s. ognevi* (Shenbrot, 1991)<br>*O. balikunica* (Hsia and Fang, 1964)<br>*O. bullata* (G. M. Allen, 1925)	***Orientallactaga***<br>*O. sibirica*<br>*O. s. sibirica*<br>*O. s. altorum*<br>*O. s. bulganensis*<br>*O. s. dementiewi*<br>*O. s. longior*<br>*O. s. ognevi*<br>*O. s. rueckbeili*<br>*O. s. saltator*<br>*O. s. suschkini*<br>*O. balikunica*<br>*O. bullata*	subgenus *Orientallactaga*<br><br>*Allactaga (O.) sibirica*<br>*A. (O.) bullata*<br>*A. (O.) balikunica*	subgenus *Orientallactaga*<br><br>*Allactaga (O.) sibirica*<br>*sibirica* subspecies group<br>*A. s. sibirica*<br>*A. s. altorum*<br>*A. s. ognevi*<br>*A. s. rueckbeili*<br>*A. s. longior*<br>*saltator* subspecies group<br>*A. s. saltator*<br>*A. s. bulganensis*<br>*A. s. dementiewi*<br>*suschkini* subspecies group<br>*A. s. suschkini*<br>*A. (O.) balikunica*<br>*A. (O.) bullata*	*A.(A.) sibirica*<br>*A. s. sibirica*<br>*A. s. altorum*<br>*A. s. semideserta* Bannikov, 1947<br>*A. s. rueckbeili*<br>*A. s. saltator*<br>*A. s. suschkini*<br>*A. bullata*	*A. saltator*<br>*A. s. saltator*<br>*A. s. suschkini*<br>*A. s. mongolica* Radde, 1861<br>*A. s. annulata*<br><br>*A. bullata*
Allactodipus Kolesnikov,1937<br>*A. bobrinskii* Kolesnikov,1937	***Allactodipus***<br>*A. bobrinskii*	***Allactodipus***<br>*A. bobrinskii*	***Allactodipus***<br>*A. bobrinskii*	subgenus *Allactodipus*<br>*Allactaga (Allactodipus) bobrinskii*	
Scarturus Gloger, 1841<br>*S. tetradactylus* (Lichtenstein, 1823)<br>*S. hotsoni* (Thomas, 1920)<br>*S. h. hotsoni*<br>*? S. h. firouzi* (Womochel, 1978)<br>*S. euphraticus* Thomas, 1881<br>*S. aulacotis* Wagner, 1840<br>*S. williamsi* Thomas, 1897<br>*S. w. williamsi*<br>*? S. w. ssp* (NW Iran)<br>*S. elater* (Lichtenstein, 1825)<br>*S. e. elater*<br>*S. e. caucasicus* (Nehring, 1900)<br>***S. (e.) strandi* (Heptner, 1934)**<br>***S. (e.) dzungariae* (Thomas, 1912)**<br>*?? S. (e.) ?d. zaisanicus* (Shenbrot, 1991)<br>***S. heptneri* (Pavlenko et Denisenko, 1976)**<br>*S. h. heptneri*<br>*? S. h. ssp* (NE Iran)<br>***S. indicus* (Gray, 1842)**<br>*S. i. indicus*<br>***S. i. toussi* (Darvish et al., 2008)**<br>***S. (i.) aralychensis* (Satunin, 1901)**<br>***S. caprimulga* (Ellerman, 1948)**<br>*S. c. caprimulga*<br>*? S. c. ssp.* (Kopet Dag)<br>*S. vinogradovi* (Argyropulo, 1941)	***Scarturus***<br>subgenus *Scarturus*<br>*S.(S.) tetradactylus*<br><br>subgenus *Paralactaga* Young, 1927<br>*S. (P.)euphraticus*<br>*S. e. euphraticus*<br>*S. e. caprimulga*<br>*S.(P.) aulacotis*<br>*S. (P.) williamsi*<br>*S. (P.) hotsoni*<br>*S. h. hotsoni*<br>*S. h. firouzi*<br><br>subgenus ?"*Microallactaga*" Shenbrot, 1974<br>*S.("M.") elater*<br>*S. e. elater*<br>*S. e. aralychensis*<br>*S. e. caucasicus*<br>*S. e. dzungariae*<br>*S. e. zaisanicus*<br>*S. e. indicus*<br><br>*S.("M.") vinogradovi*<br>*S. v. vinogradovi* 1<br>*S. v. toussi*	subgenus *Scarturus*<br>*Allactaga (S.) tetradactyla*	subgenus *Scarturus*<br>*Allactaga (S.) tetradactyla*	***Scarturus***<br>*S. tetradactylus*	***Scarturus***<br>*S. tetradactylus*
Pygeretmus Gloger, 1841<br>subgenus *Pygeretmus*<br>*P. (P.) platyurus* (Lichtenstein, 1823)<br>*P. (P.) shitkovi* (Kuznetsov, 1930)<br>*P.s. shitkovi*<br>*? P.s. ssp.* (Ili valley)	***Pygeretmus***<br>subgenus *Pygeretmus*<br>*P. (P.) platyurus*<br>*P. (P.) shitkovi*	***Pygeretmus***<br>subgenus *Pygeretmus*<br>*P. (P.) platyurus*<br>*P. (P.) shitkovi*	***Pygeretmus***<br>subgenus *Pygeretmus*<br>*P. (P.) platyurus*<br>*P. (P.) shitkovi*	***Pygeretmus***<br>*P. platyurus*<br>*P. zhitkovi*	***Pygeretmus***<br>*P. platyurus*<br>*P. zhitkovi*
subgenus *Alactagulus* Nehring, 1897<br>*P. (A.) pumilio* (Kerr, 1792)<br>*P. p. pumilio*<br>*P. p. potanini* (Vinogradov, 1926)<br>*P. p. bullatoides* Shenbrot, 1995<br>*? P. p. iliensis* Shenbrot, 1995<br>*? P. p. selevini* Shenbrot, 1995<br>*? P. p. ssp.* (Zaisan depression)	subgenus *Alactagulus*<br>*P. (A.) pumilio*<br>*P. p. pumilio*<br>*P. p. bullatoides*<br>*P. p. iliensis*<br>*P. p. potanini*<br>*P. p. selevini*	subgenus *Alactagulus*<br>*P. (A.) pumilio*	subgenus *Alactagulus*<br>*P. (A.) pumilio*<br>*P. p. pumilio*<br>*P. p. bullatoides*<br>*P. p. iliensis*<br>*P.p. potanini*<br>*P. p. selevini*	***Alactagulus***<br>*A. pygmaeus*, Pallas, 1778 (=*pumilio*)<br>*A. p. pygmaeus*<br>*A. p. dinniki* Satunin, 1920<br>*A. p. tanaiticus* Ognev, 1948<br>*A. p. aralensis* Ognev, 1948<br>*A. p. turkomanus* Heptner et Samorodov, 1939<br>*A. p. potanini*	***Alactagulus***<br>*A. acontion* Pallas, 1878 (=*pumilio*)<br>*A. a. acontion*<br>*A. a. dinniki*<br>*A. a. potanini*

Biogeographic reconstructions

Node no. 1: divergence between the *Allactaga+Orientallactaga* and *Scarturus+Pygeretmus* clades 5.69−5.54 Mya (Fig. 3). We have no paleoclimate data for the Upper Miocene; however, reconstructions for the most warm and wet period of the earliest Pliocene (5.33 Mya) demonstrated that at that time, the range of the putative ancestral species was divided into East and West Central Asian parts separated along the Altay–Tarbagatay–Dzungar Alatau–Tianshan line. Thus, one may hypothesize that the *Allactaga+Orientallactaga* clade originated in East Central Asia, whereas the *Scarturus+Pygeretmus* clade evolved in West Central Asia.

Node no. 2: divergence between *Orientallactaga* and *Allactaga+Allactodipus* 4.37−4.18 Mya (vicariance model) (Fig. 4). As a result of range fragmentation, several small isolates appeared in central Kyzylkum 4.34 Mya and existed up to 4.28 Mya with partial interruptions along narrow corridors (semi-isolation) 4.31 and 4.27 Mya. The ancestor of *Allactaga* + *Allactodipus* possibly originated from these isolates.

Node no. 3: divergence between *Allactaga* and *Allactodipus* (3.28-3.2 Mya) (vicariance model). An isolate on Krasnovodsk Plateau separated from the main range 3.26 Mya and existed up to 3.18 Mya without interruption. It is plausible that *Allactodipus* originated from this isolate.

Node no. 4: divergence between *Allactaga major* and *A. severtzovi* (2.02-1.9 Mya) (vicariance model). Three isolates appeared 2.0 Mya and existed up to 1.91 Mya with two interruptions at 1.98-1.97 and 1.94-1.93 Mya: (1) in the eastern part of Turgay depression; (2) in the middle part of the Volga basin; (3) in Zaisan Hollow. *A. major* originated from one of these three isolates, whereas the ancestor of *A. severtzovi* occurred in the main part of the geographic range.

Node no. 5: divergence between *Orientallactaga sibirica* and *O. bullata+O. balikunica* (2.17-1.81 Mya) (founder speciation model). The ancestor of the group entered the region from the eastern part of Cental Asia to Kazakhstan through a narrow corridor in Zhungarian Gate 2.09 Mya. This corridor was closed 2.06 Mya and was restored later for short periods 1.91 and 1.84 Mya. It is possible to conclude that the ancestor of *O. sibirica* originated in the Kazakh part of the group's geographic range.

Node no. 6: divergence between *O. bullata* and *O. balikunica* (1.54-1.43 Mya) (vicariance model). Two relatively small isolates emerged due to range fragmentation in the current Shargyin Gobi and Beger Hollow 1.55 Mya and existed up to 1.5 Mya with one short interruption 1.52 Mya. *O. bullata* had possibly originated in one of these two isolates, whereas the ancestor of *A. balikunica* occurred in the main part of the group's geographic range.

Node no. 7: divergence between ancestors of *Scarturus* and *Pygeretmus* (4.48-4.32 Mya) (vicariance model). Peripheral isolate in the area around the current border

between Afghanistan and Pakistan separated from the main range at 4.42 Mya and existed up to 4.35 Mya with one short interruption along a narrow corridor 4.37 Mya. *Scarturus* has possibly originated in this isolate.

Node no. 8: divergence between *S. tetradactylus* + *S. hotsoni* and VECE clades (4.1-3.95 Mya) (founder speciation model). A corridor connecting the main part of the group's geographic range with current Iranian Baluchestan opened 4.0 Mya and was closed from 3.99 Mya. It seems that the ancestor of the former clade dispersed across this corridor and evolved in the Baluchestan isolate.

Node no. 9: divergence between the *S. euphraticus* species group and other lineages within VECE clade (3.21-3.11 Mya). Two scenarios are possible for this node: (1) throughout almost all of this period, a narrow corridor connected the main part of the group's geographic range with Anatolia; this corridor was closed twice for short periods 3.14 and 3.12 Mya; (2) a narrow corridor connecting Anatolia with northern Mesopotamia emerged 3.15 Mya and existed up to 3.12 Mya; all other time before and after this event Anatolia was isolated from northern Mesopotamia. According to the first scenario, the ancestor of the *S. euphraticus* species group emerged as a result of isolation in Anatolia (vicariance model). According to the second scenario, the ancestor entered northern Mesopotamia from Anatolia and evolved in the former area after its isolation (founder speciation model).

Node no. 10: divergence between *S. caprimulga*+*S. vinogradovi* and *S. elater* species group (2.85-2.78 Mya) (vicariance model). During this entire period, the main part of the group's geographic range and the area around the current border between Afghanistan and Pakistan were either isolated (2.88-2.86, 2.84, 2.80-2.79, and 2.77-2.75 Mya) or connected by a long narrow corridor (2.89, 2.85, 2.83-2.81, 2.78, and 2.74-2.69 Mya). It appears that the ancestor of *S. caprimulga + S. vinogradovi* clade originated from this isolated area.

Node no. 11: divergence between *S. caprimulga* and *S. vinogradovi* (2.50-2.45 Mya) (founder speciation model). A narrow long corridor connecting southeastern and central Afghanistan with northern foothills of Tien-Shan across the western foothills of Pamir and Tien-Shan was established 2.53 Mya and was interrupted at 2.47, 2.45, and 2.43 Mya. The ancestor of *S. vinogradovi* apparently dispersed northwards along this corridor and later evolved in isolation.

Nodes 14-15: divergence within *S. euphraticus* species group (1.21-0.87 Mya). The order of branching within this clade has no high support. Paleo-environment distribution modeling demonstrated that during this entire period, there were two groups of populations: (1) Anatolia, Trans-Caucasus, and N Zagros, and (2) Levant, N Mesopotamia, and S Zagros. During most of the time, these two groups of populations were connected by several narrow corridors; these corridors closed twice, 1.23-1.24 and 1.07 Mya. Apparently, *S. williamsi* originated in the first (northern) part of the group's geographic range, whereas the common ancestor of *S. euphraticus* and *S. aulacotis* originated in the second (southern) part of the group's range (vicariance model). The common ancestor of *S. euphraticus* and *S. aulacotis* disappeared from the eastern part of its range (SW foothills of Zagros Mountains and SE Mesopotamia) 1.07 Mya and dispersed southeast again 1.05 Mya; northern and southeastern parts of

the geographic range were completely isolated 1.03-1.02, 1.00-0.99, and 0.95-0.93 Mya, but were connected by a narrow corridor during other periods. It seems that *S. euphraticus* originated in southeastern isolate, whereas *S. aulacotis* evolved in the main part of the geographic range of *S. euphraticus* and *S. aulacotis* common ancestor (founder speciation model).

Node no 16: divergence between *S. elater* and *S. heptneri* (1.01−0.93 Mya) (vicariance model). Fergana Valley was isolated from the main part of the geographic range 1.05−1.04, 1.01, 0.97−0.96, and 0.93−0.87 Mya; during other periods, it was connecter by a narrow corridor. It seems obvious that *S. heptneri* originated in the Fergana isolate.

Node no. 17: divergence between *Pygeretmus* and *Alactagulus* subgenera (2.57-2.34 Mya) (vicariance model). The Ili River Hollow (SE Kazakhstan) was connected with the main part of *Pygeretmus* genus geographic range by a narrow corridor during most of this period, but became isolated at 2.53-2.51, 2.49-2.48, 2.45, and 2.37 Mya. Ancestral *Alactagulus* possibly evolved in the main part of the geographic range, whereas the common ancestor of *P. shitkovi* and *P. platyurus* evolved in the Ili isolate.

Node no. 18: divergence between *Pygeretmus shitkovi* and *P. platyurus* (1.42-1.36 Mya). Undivided geographic range of the common ancestor of *P. shitkovi* and *P. platyurus* existed in Kazakhstan, N Turan, and Dzungaria up to 1.43 Mya. The geographic range was broken into several isolated parts 1.42 Mya: large solid western area and several small eastern areas (around Balkhash, in Ili River Hollow, and in Dzungaria). This pattern existed up to 1.41 Mya after that isolation disappeared, but was restored 1.38-1.37 and 1.34-1.31 Mya. *P. platyurus* has possibly originated from the large western isolate, whereas *Pygeretmus shitkovi* emerged in one of the small eastern isolates.

Figure S7. Geography of speciation in the nodes no. 1-11, 14-18

All maps were obtained as raster maps from modeling using MAXENT 3.4.1 software and then generated as polygon maps using ArcGIS Desktop 10.8.1 software.
MAXENT: available at https://biodiversityinformatics.amnh.org/open_source/maxent/
ArcGIS Desktop: Copyright © 1995-2020 Esri

Node no. 1. Allactaginae. Variables used in the modeling: Bio 1, Bio 2, Bio 4, Bio 8, Bio 15 and Bio 16. AUC = 0.756. Threshold value = 0.404.

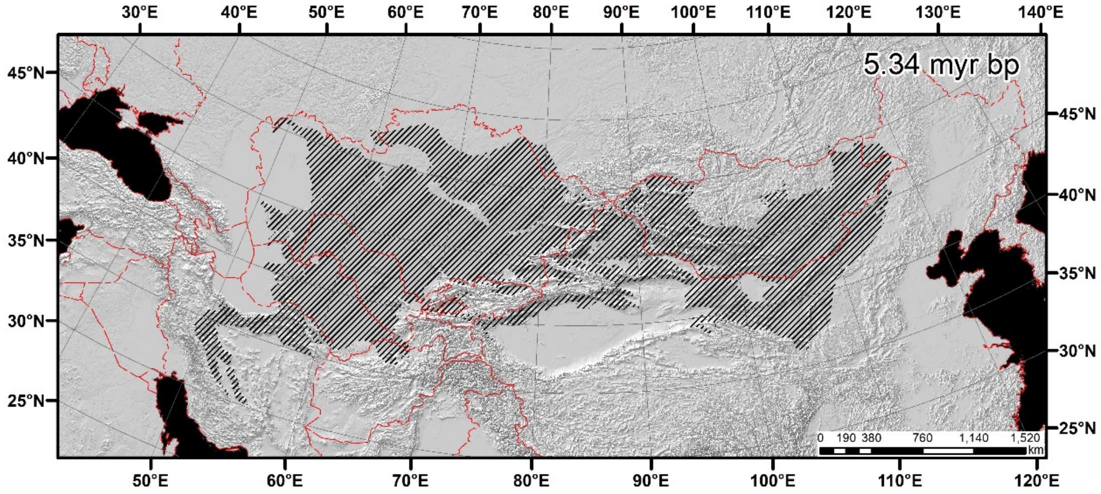

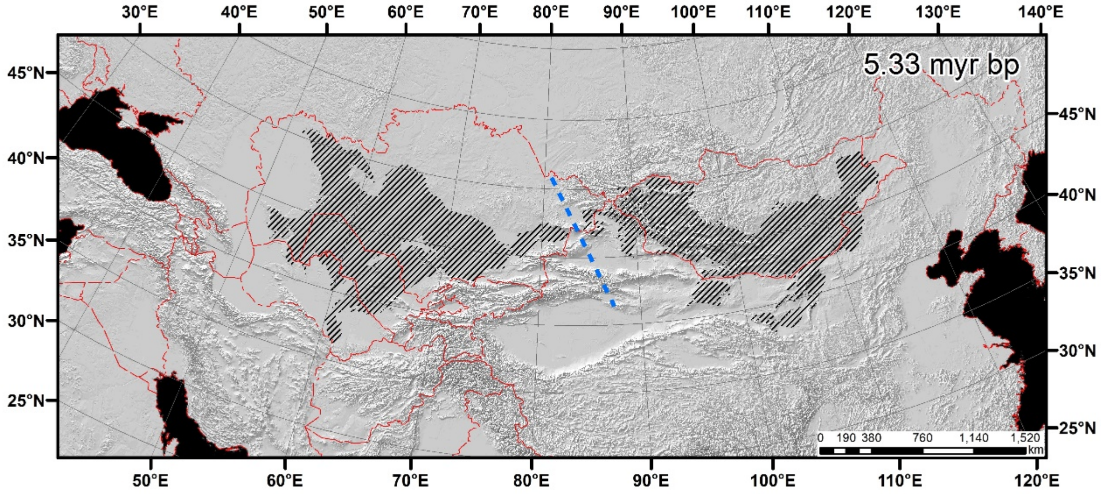

Node no. 2 – *Allactaga+Allactodipus* / *Orientallactaga*. Variables used in the modeling: Bio 1, Bio 2, Bio 4, Bio 5, Bio 6, Bio 12 and Bio 15. AUC = 0.772. Threshold value = 0.428.

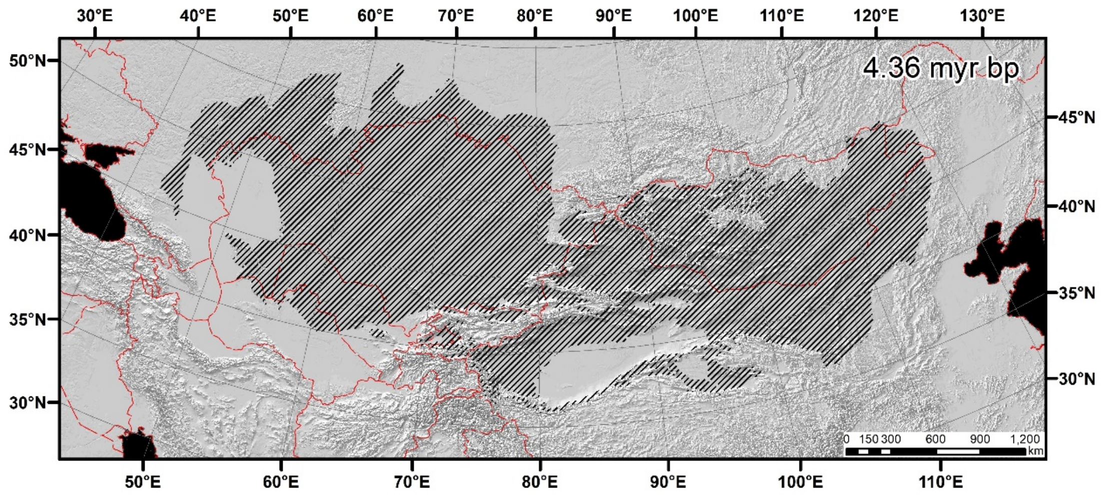

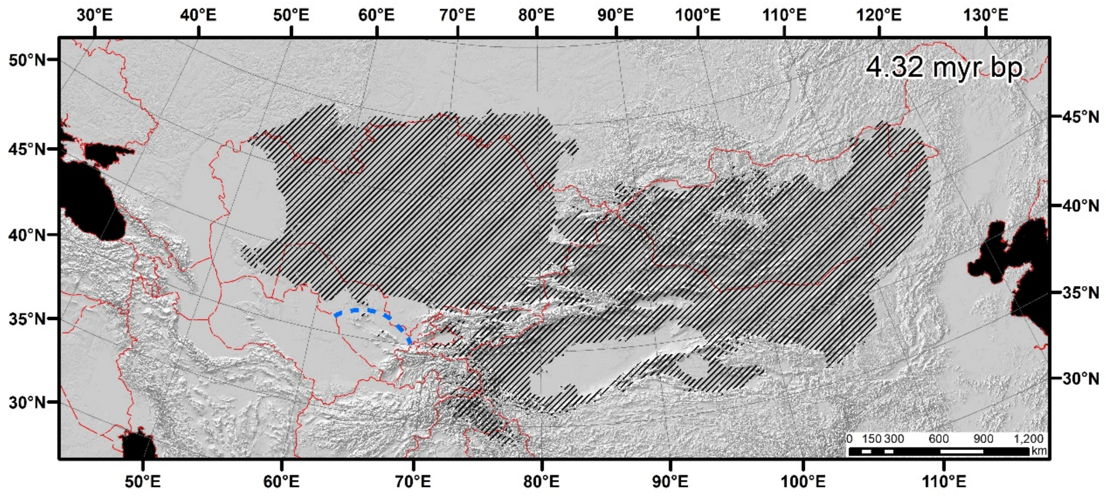

Node no. 3 – *Allactaga / Allactodipus*. Variables used in the modeling: Bio 2, Bio 4, Bio 5, Bio 7, Bio 10 and Bio 19. AUC = 0.795. Threshold value = 0.495.

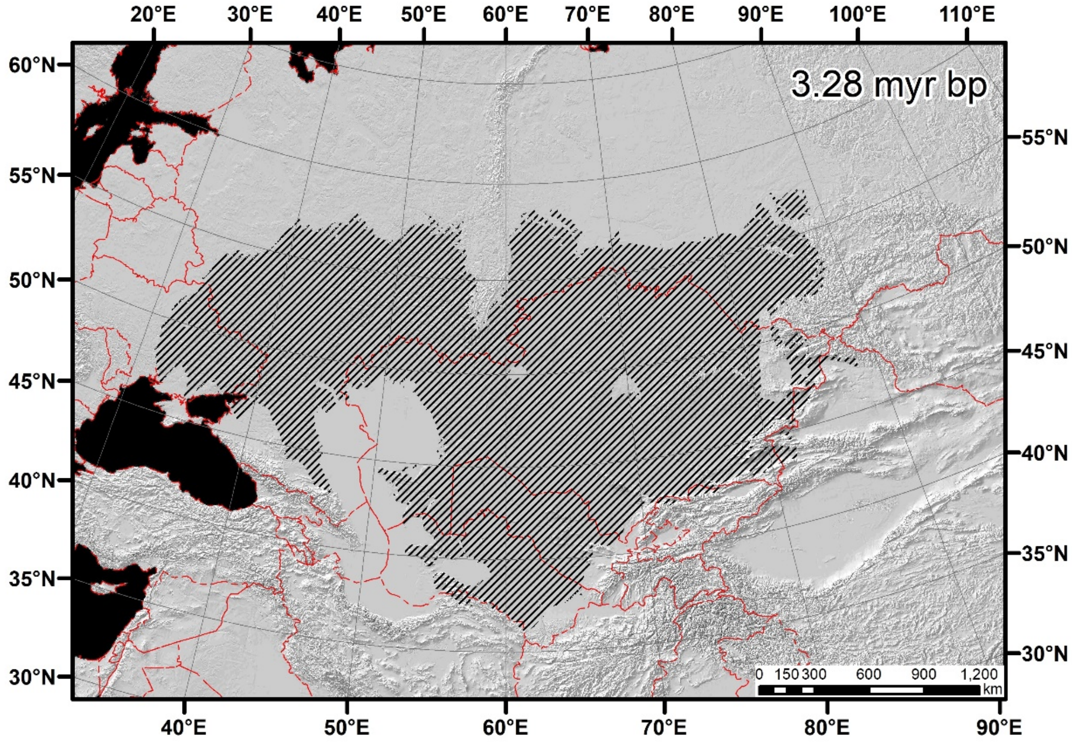

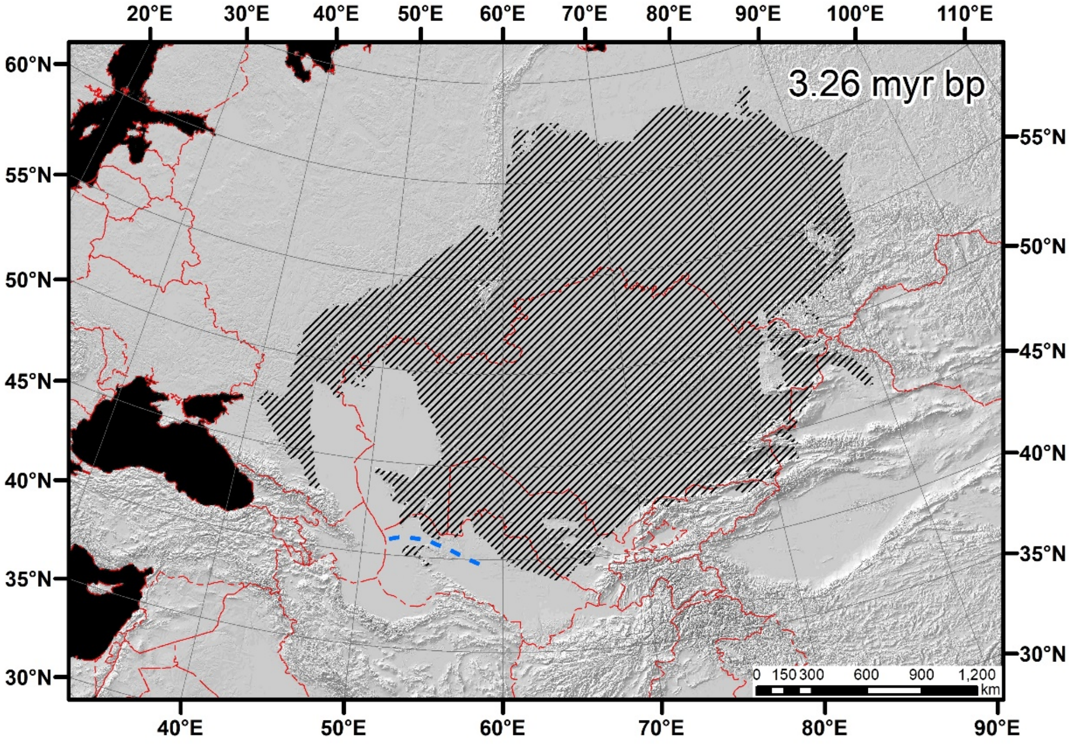

Node no. 4 - *Allactaga*. Variables used in the modeling: Bio 1, Bio 2, Bio 4, Bio 5, Bio 6, Bio 10 and Bio 19. AUC = 0.795. Threshold value = 0.509. The model for 1.92 myr bp is shown as the coldest and driest time period when geographic range was maximally constricted with minimal size of peripheral isolates.

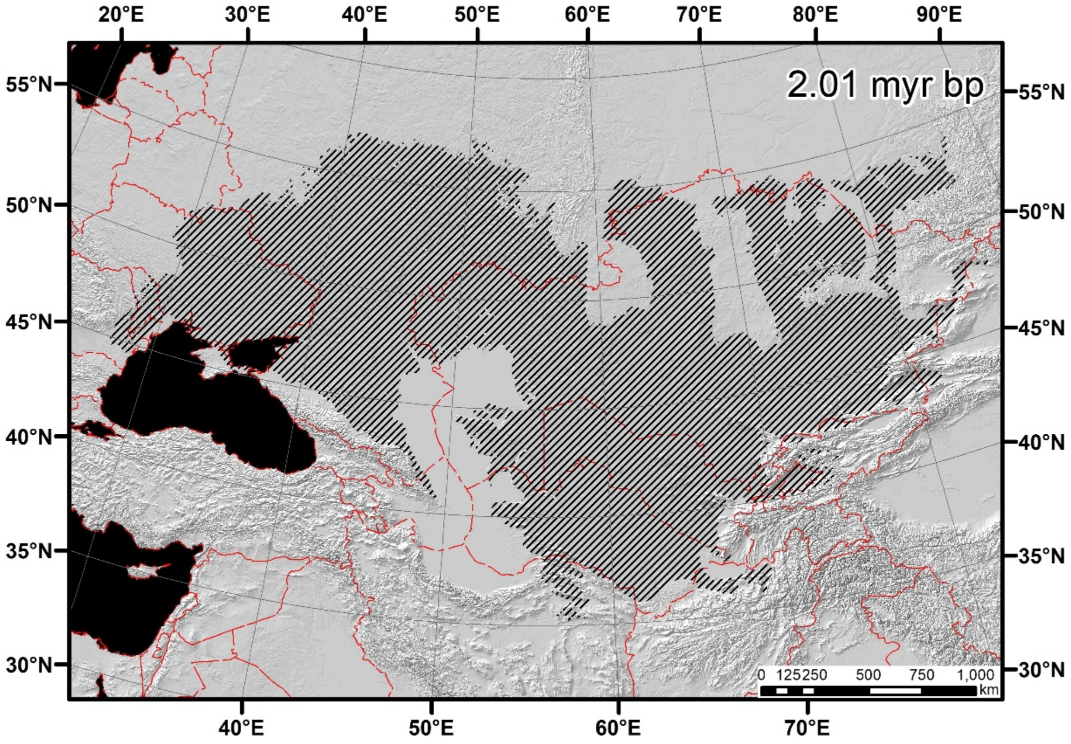

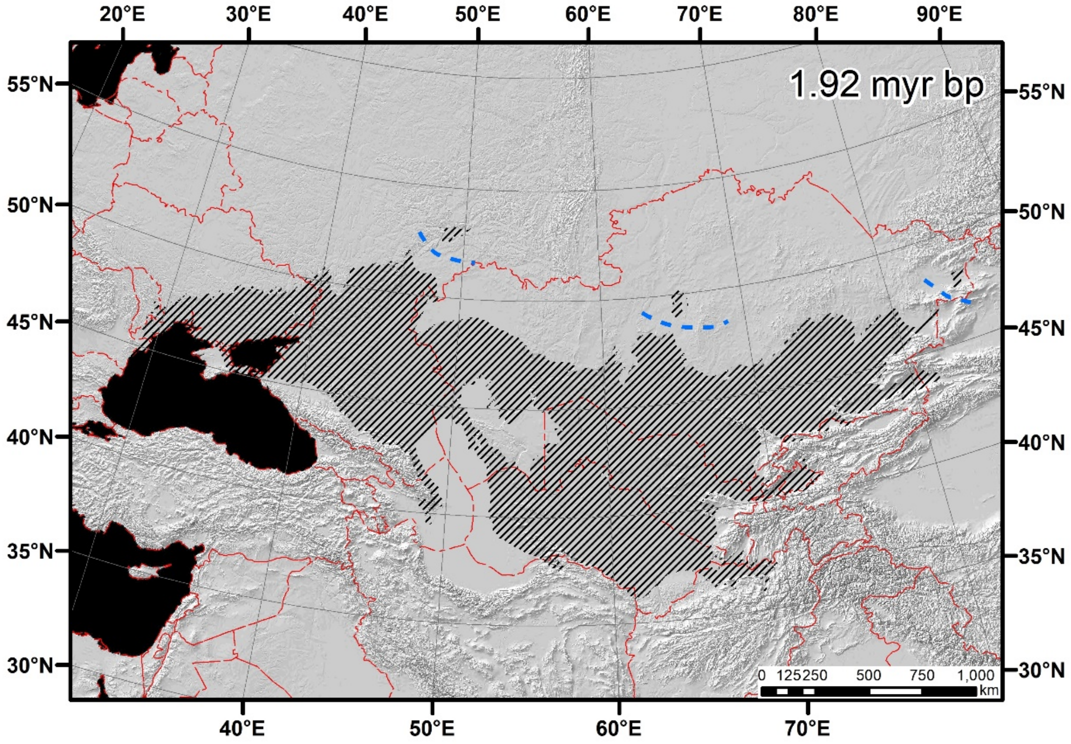

Node no. 5 - *Orientallactaga*. Variables used in the modeling: Bio 1, Bio 2, Bio 4, Bio 6, Bio 12, Bio 15 and Bio 19. AUC = 0.843. Threshold value = 0.312.

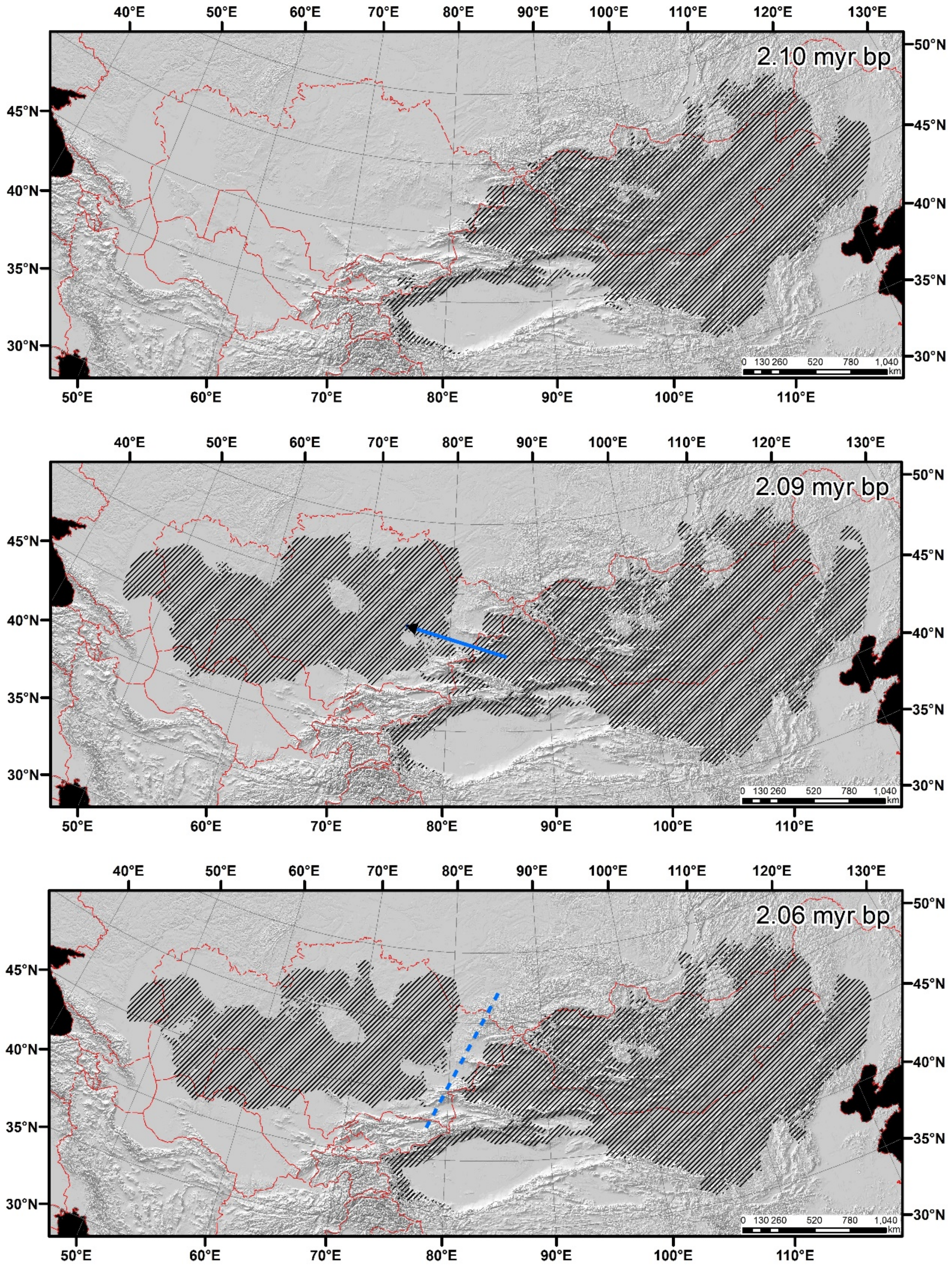

Node no. 6 – *Orientallactaga bullata / O. balikunica*. Variables used in the modeling: Bio 1, Bio 4, Bio 5, Bio 11, Bio 15 and Bio 16. AUC = 0.912. Threshold value = 0.327.

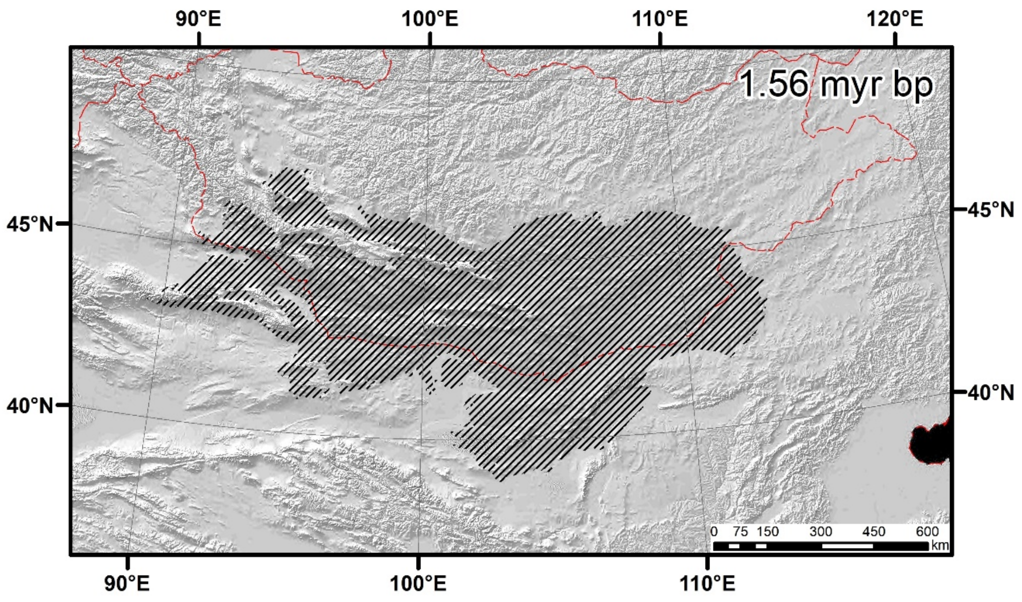

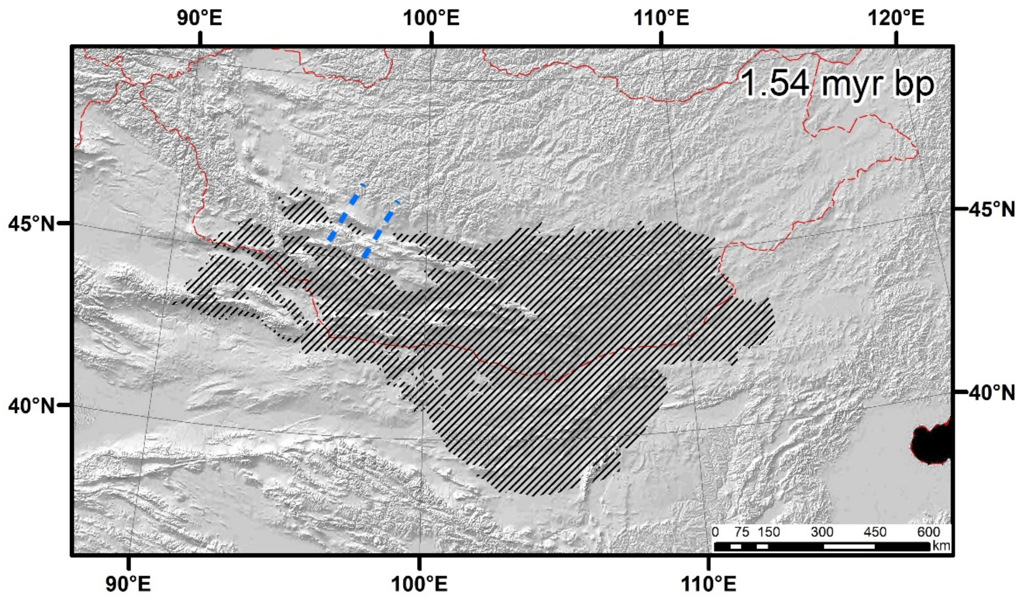

Node no. 7 – *Scarturus / Pygeretmus*. Variables used in the modeling: Bio 1, Bio 2, Bio 10, Bio 12, Bio 18 and Bio 19. AUC = 0.836. Threshold value = 0.339.

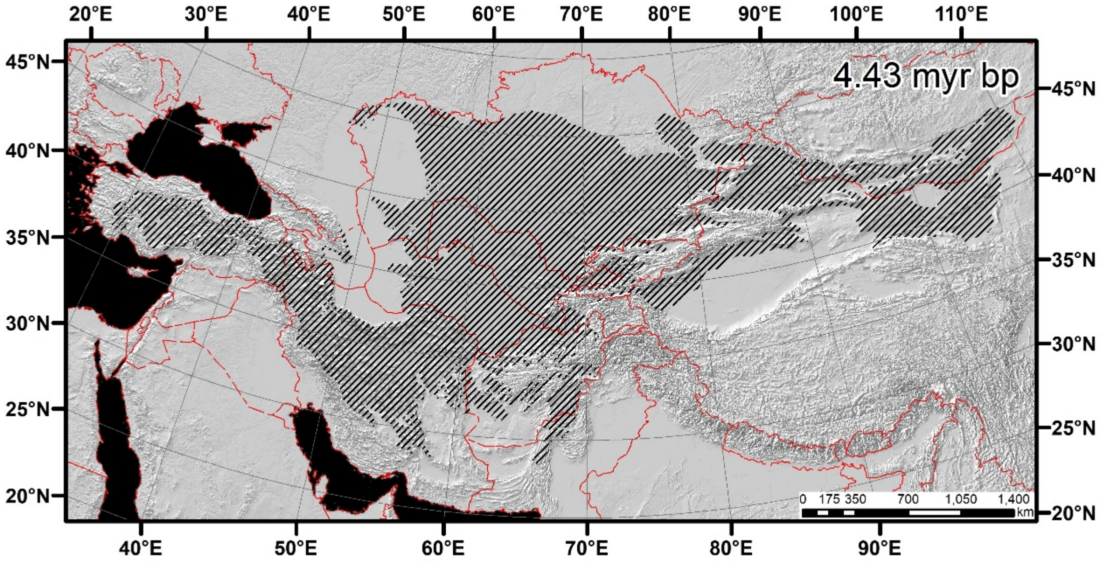

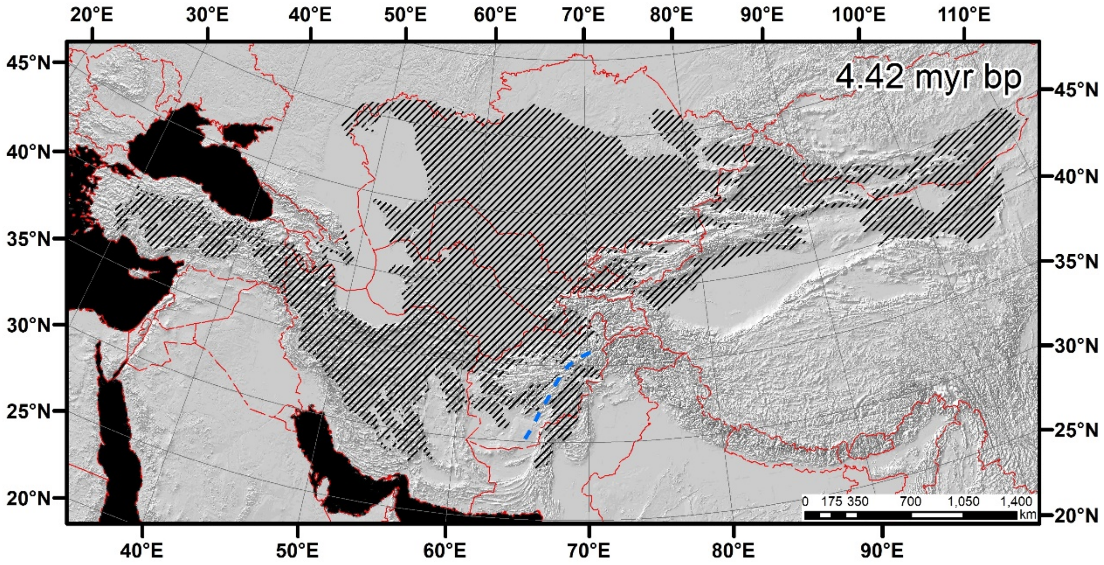

Node no. 8 – *Scarturus* genus. Variables used in the modeling: Bio 1, Bio 2, Bio 4, Bio 5, Bio 12, Bio 18 and Bio 19. AUC = 0.854. Threshold value = 0.304.

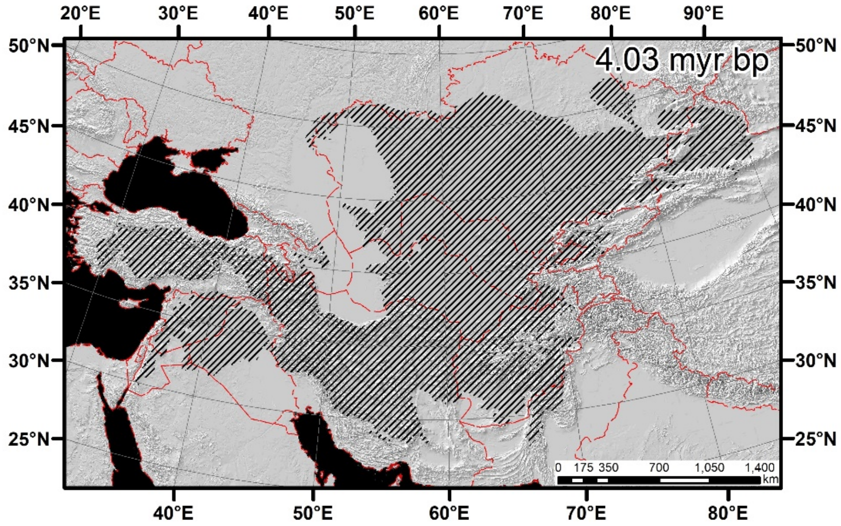

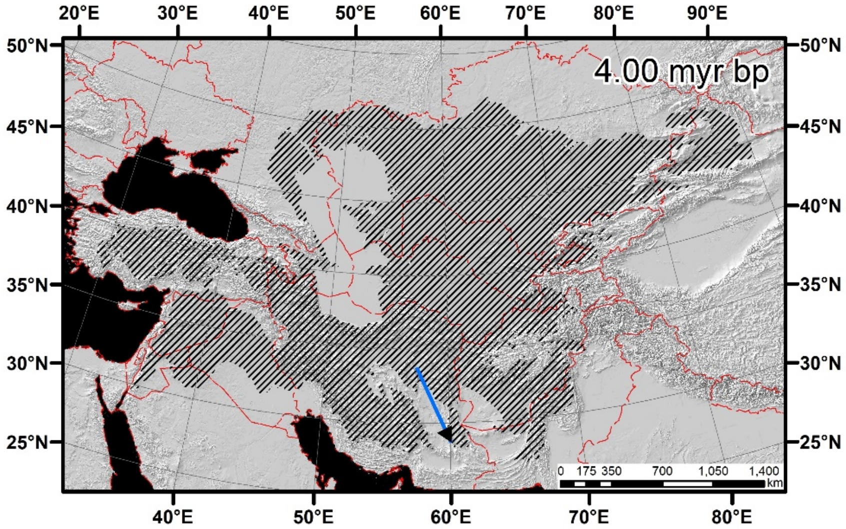

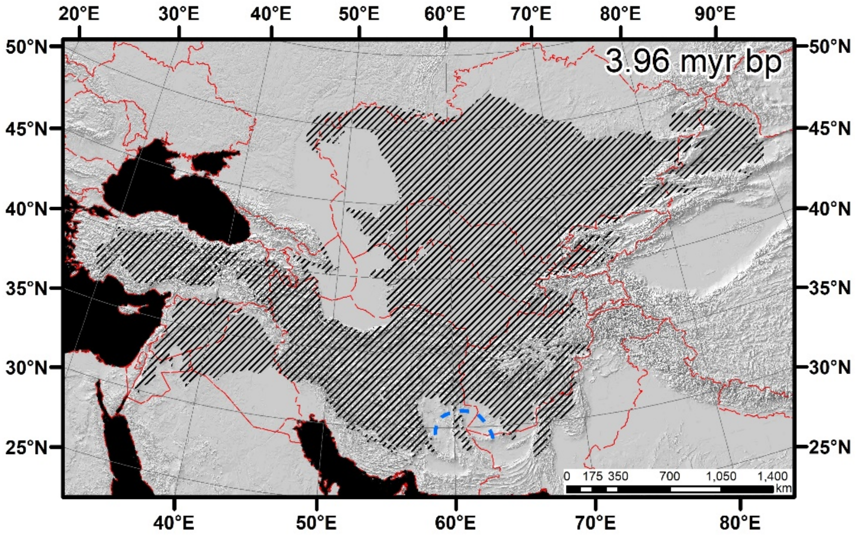

Node no. 9 – *Scarturus euphraticus* species group / sister lineages. Variables used in the modeling: Bio 1, Bio 2, Bio 4, Bio 10, Bio 12, Bio 18 and Bio 19. AUC = 0.854. Threshold value = 0.356.

Scenario 1.

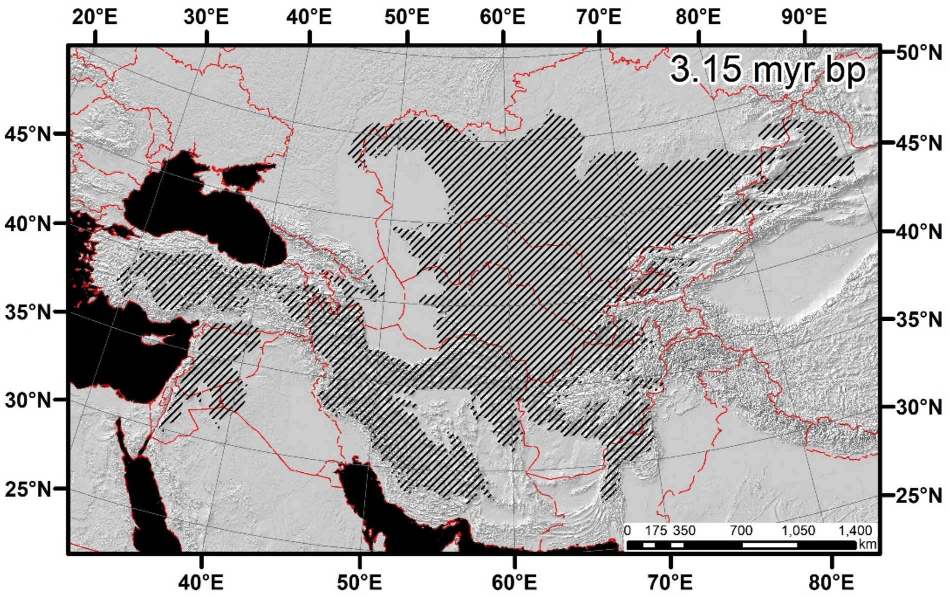

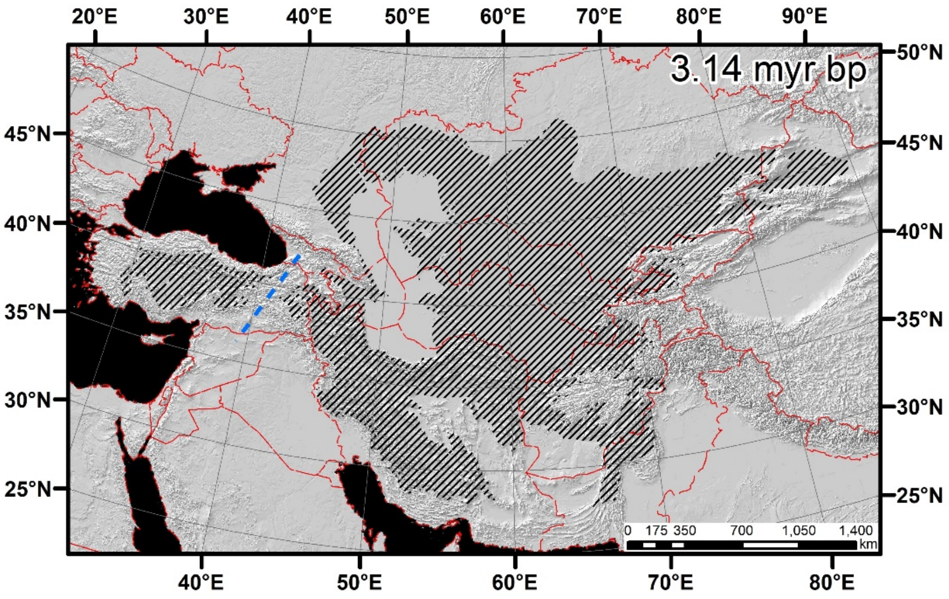

Scenario 2.

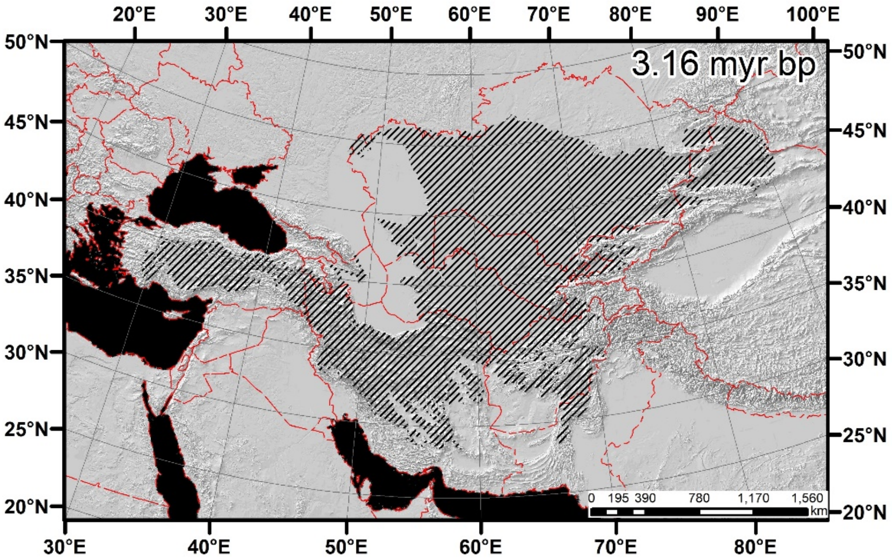
3.16 myr bp
20°E 30°E 40°E 50°E 60°E 70°E 80°E 90°E 100°E
50°N 45°N 40°N 35°N 30°N 25°N 20°N
30°E 40°E 50°E 60°E 70°E 80°E
0 195 390 780 1,170 1,560
km

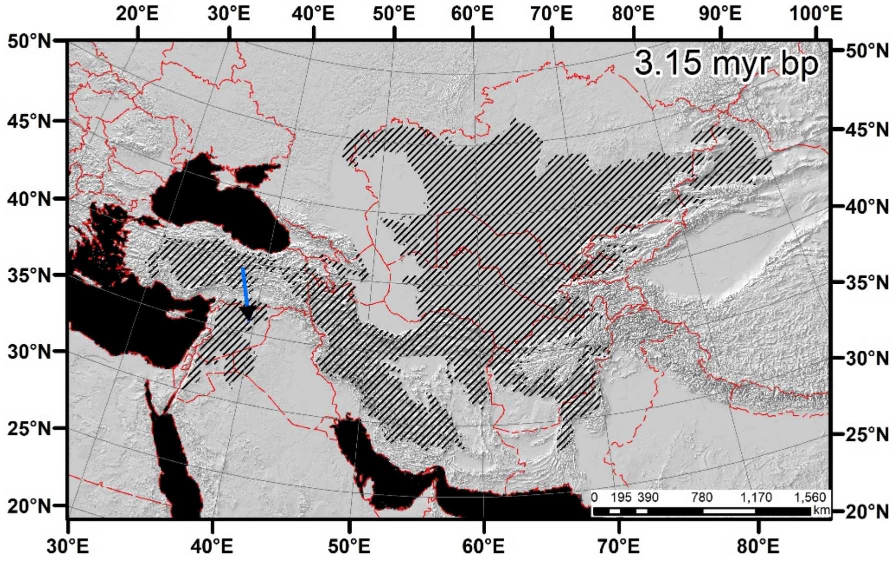
3.15 myr bp
20°E 30°E 40°E 50°E 60°E 70°E 80°E 90°E 100°E
50°N 45°N 40°N 35°N 30°N 25°N 20°N
30°E 40°E 50°E 60°E 70°E 80°E
0 195 390 780 1,170 1,560
km

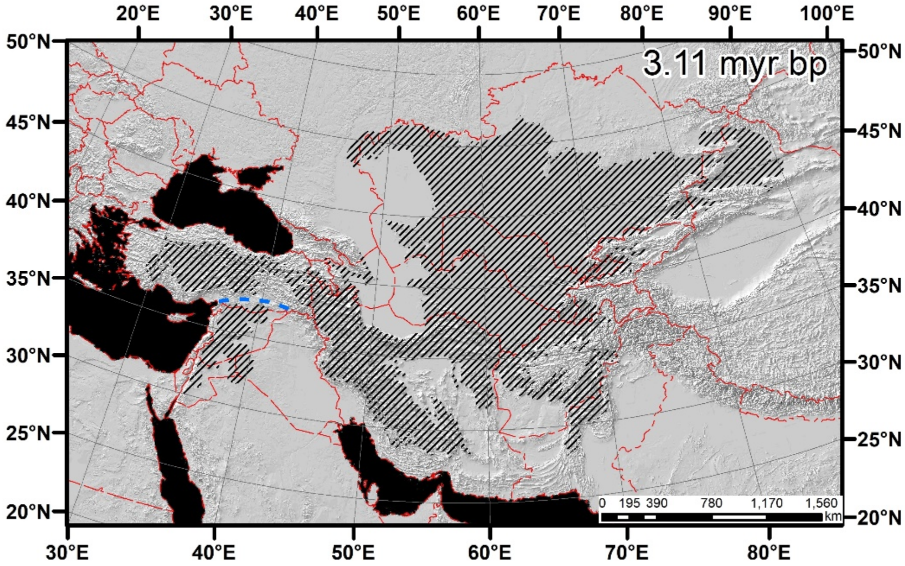
3.11 myr bp
20°E 30°E 40°E 50°E 60°E 70°E 80°E 90°E 100°E
50°N 45°N 40°N 35°N 30°N 25°N 20°N
30°E 40°E 50°E 60°E 70°E 80°E
0 195 390 780 1,170 1,560
km

Node no. 10 – *Scarturus elater* species group / sister lineages. Variables used in the modeling: Bio 1, Bio 3, Bio 7, Bio 10, Bio 18 and Bio 19. AUC = 0.860. Threshold value = 0.300.

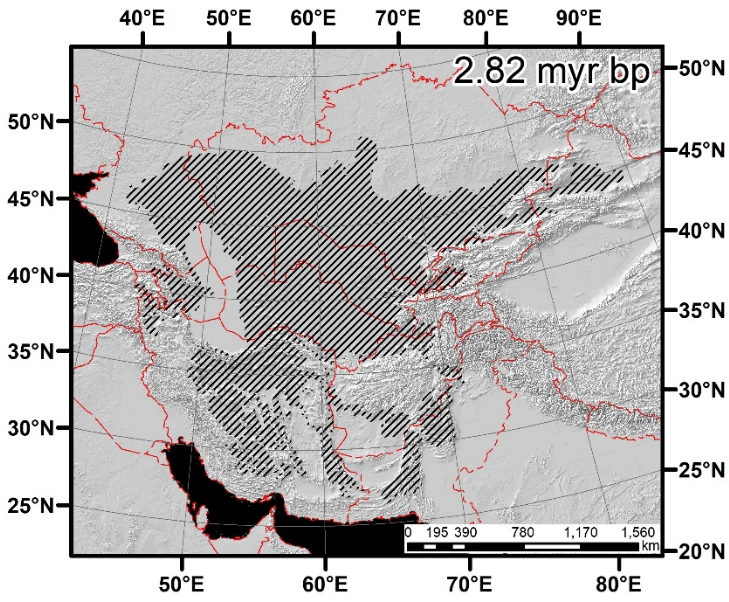

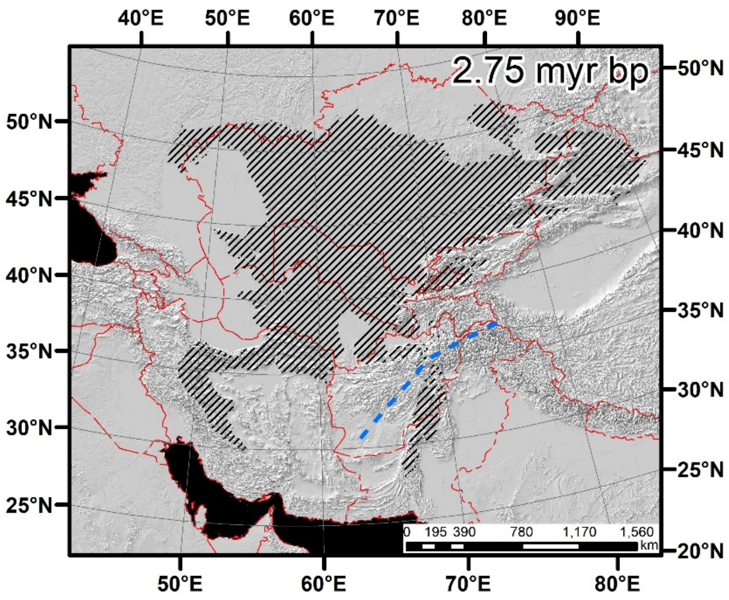

Node no. 11 – *Scarturus caprimulga / S. vinogradovi*. Variables used in the modeling: Bio 1, Bio 7, Bio 11, Bio 13, Bio 17 and Bio 19. AUC = 0.959. Threshold value = 0.158. Dots – potentially suitable but not occupied areas.

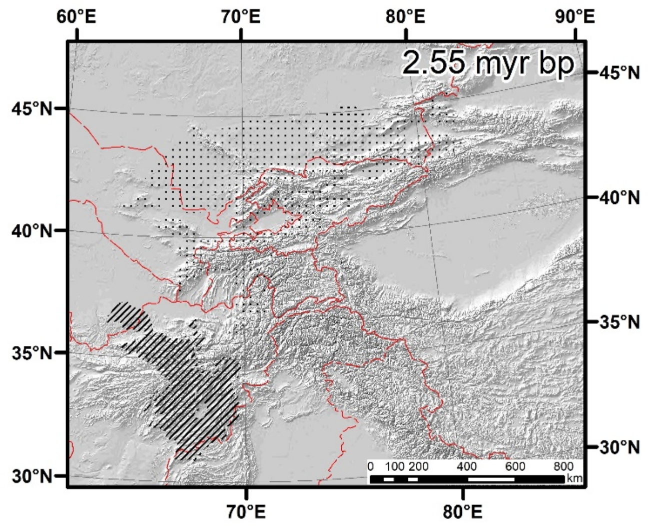

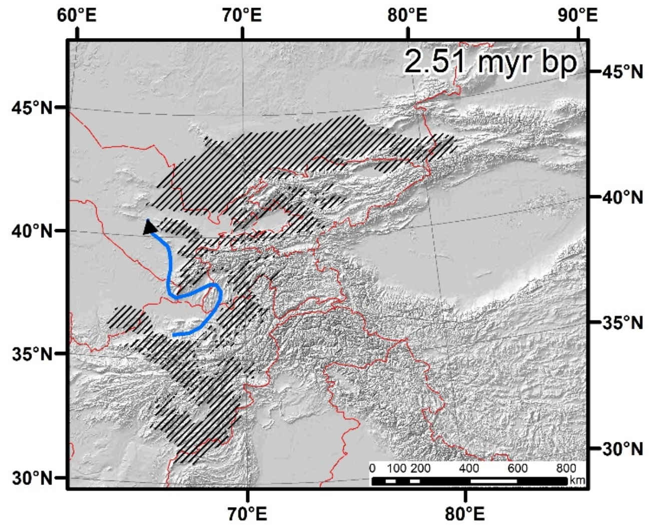

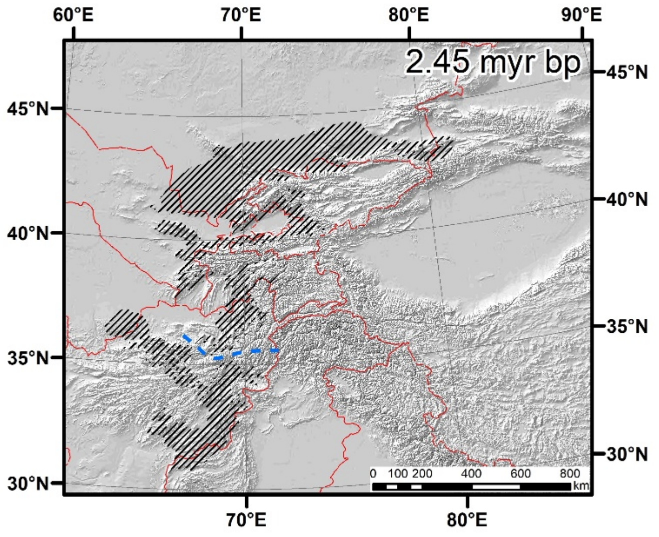

Nodes 14 – divergence within *Scarturus euphraticus* speies group between *S. williamsi* and *S. euphraticus* + *S. aulacotis*. Variables used in the modeling: Bio 2, Bio 6, Bio 8, Bio 12, Bio 18 and Bio 19. AUC = 0.873. Threshold value = 0.446.

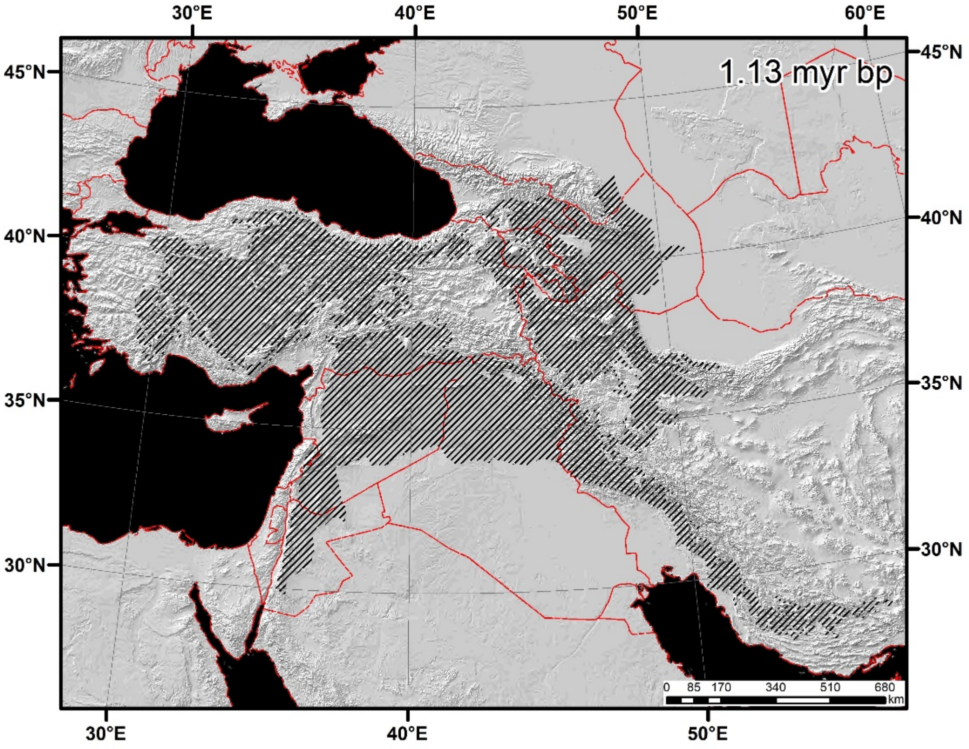

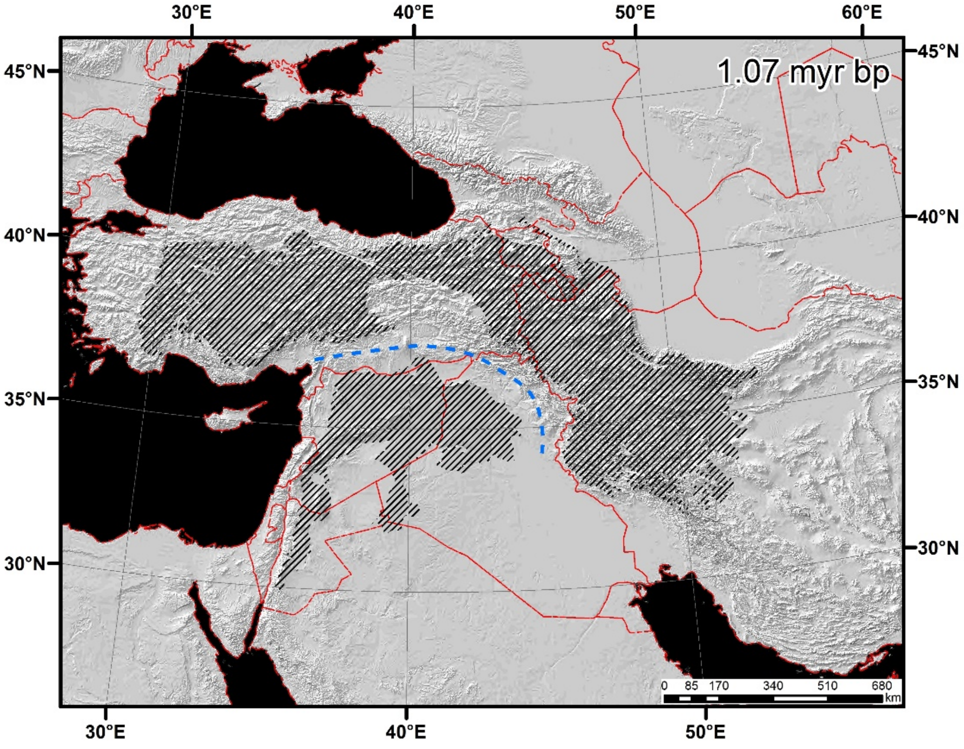

Node 15 - divergence between *S. euphraticus* and *S. aulacotis*. Variables used in the modeling: Bio 2, Bio 6, Bio 8, Bio 13 and Bio 19. AUC = 0.925. Threshold value = 0.308.

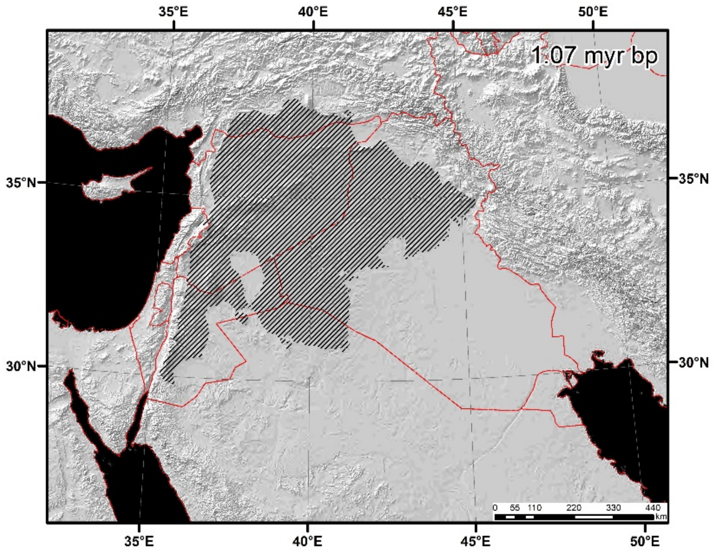

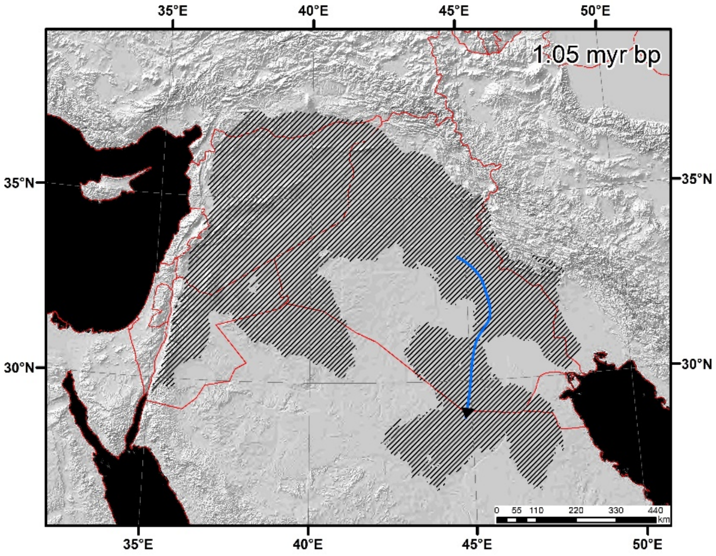

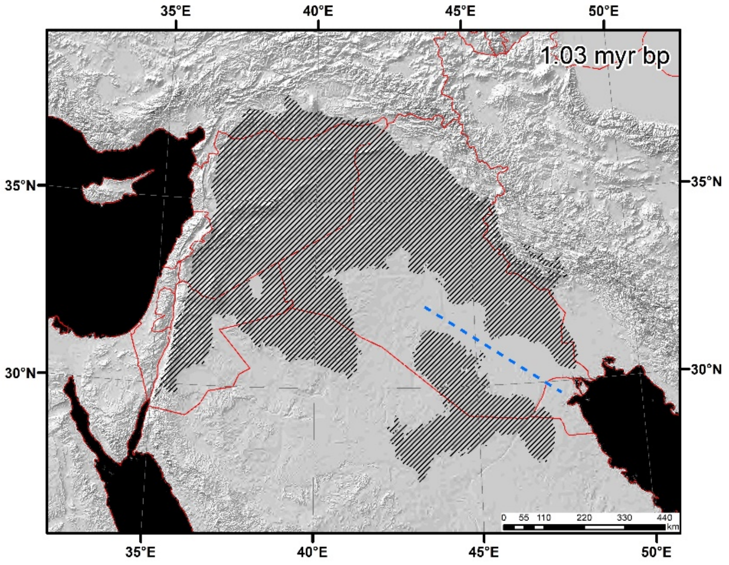
1.03 myr bp
35°E
40°E
45°E
50°E
35°N
30°N
0 55 110 220 330 440
km

Node 16 - divergence between *S. elater* and *S. heptneri*. Variables used in the modeling: Bio 1, Bio 3, Bio 7, Bio 10, Bio 12 and Bio 19. AUC = 0.872. Threshold value = 0.365.

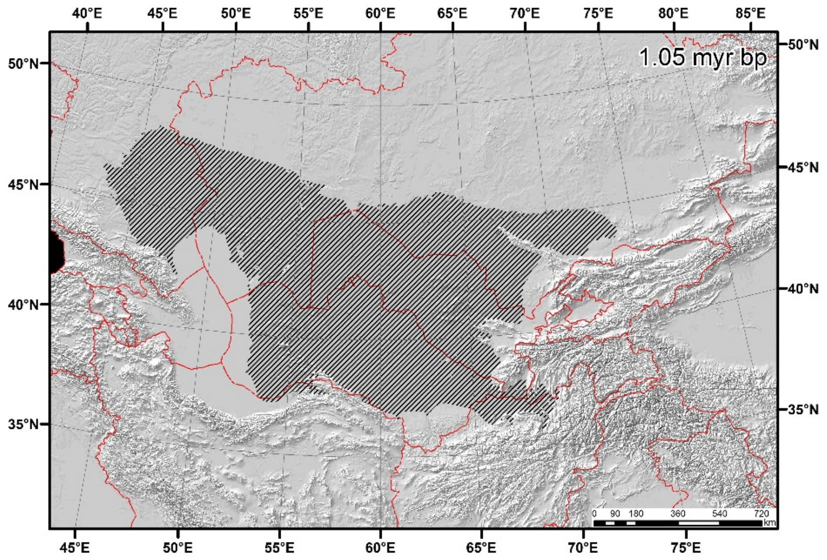

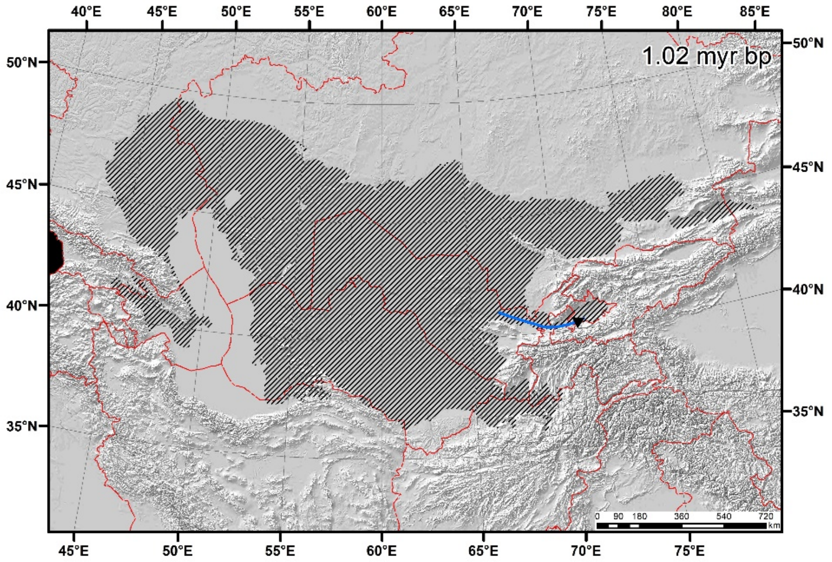

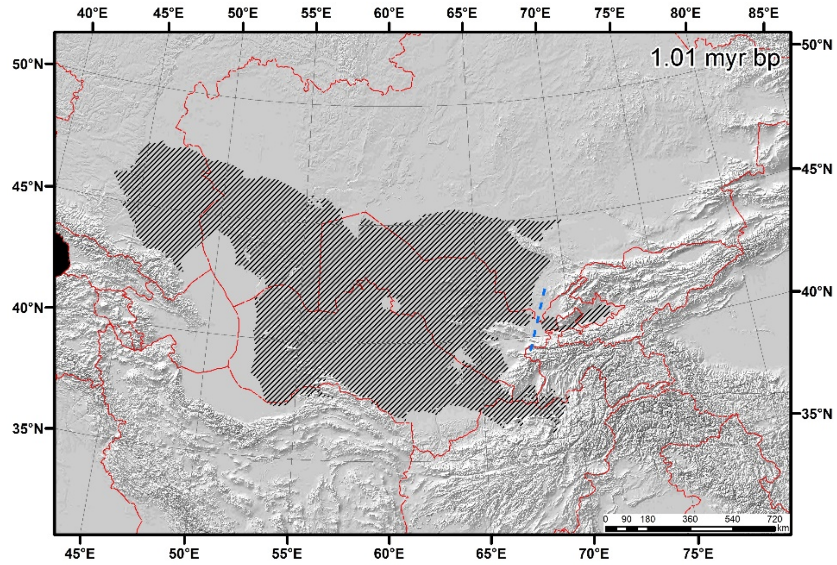

1.01 myr bp
40°E
45°E
50°E
55°E
60°E
65°E
70°E
75°E
80°E
85°E
50°N
45°N
40°N
35°N
0 90 180 360 540 720
km

Node 17 - divergence between *Pygeretmus* and *Alactagulus* subgenera. Variables used in the modeling: Bio 3, Bio 4, Bio 6, Bio 10, Bio 12 and Bio 15. AUC = 0.867. Threshold value = 0.323.

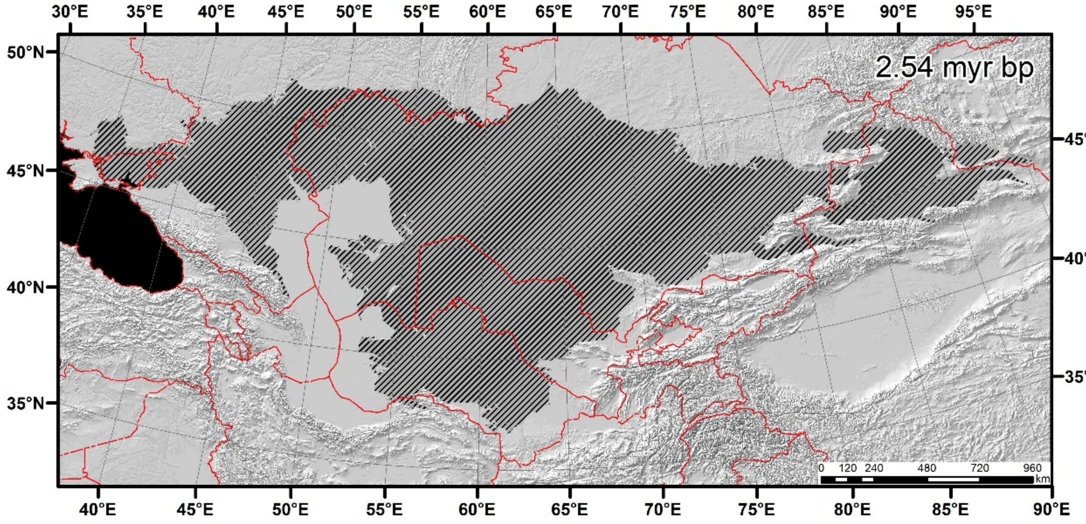

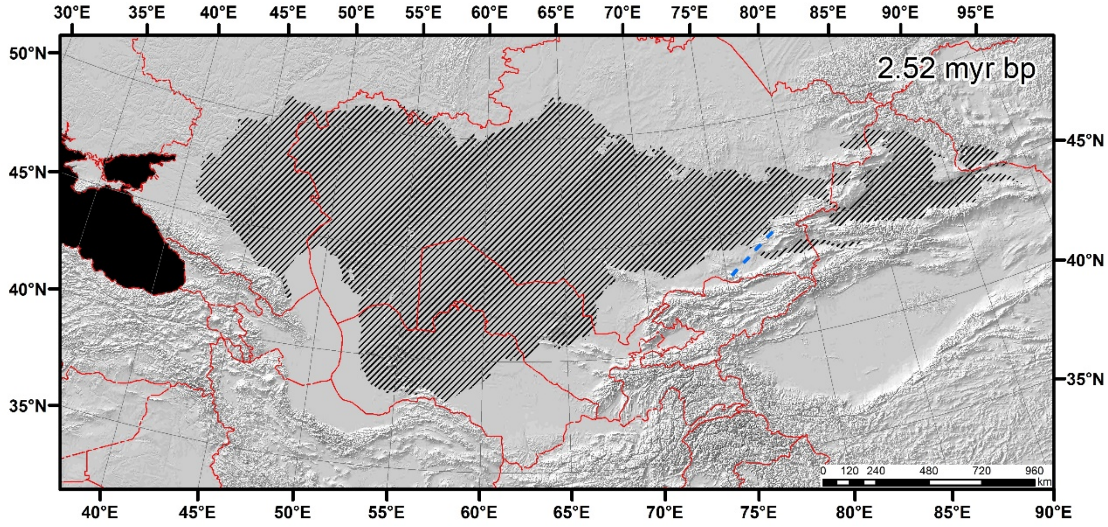

Node 18 - divergence between *Pygeretmus shitkovi* and *P. platyurus*. Variables used in the modeling: Bio 2, Bio 7, Bio 10, Bio 13, Bio 14, Bio 15 and Bio 17. AUC = 0.898. Threshold value = 0.126.

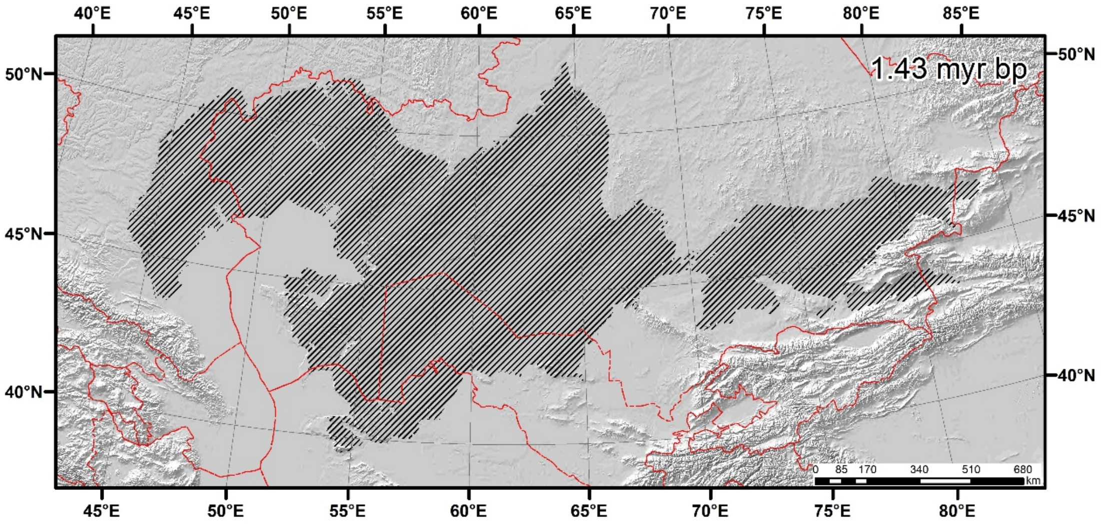

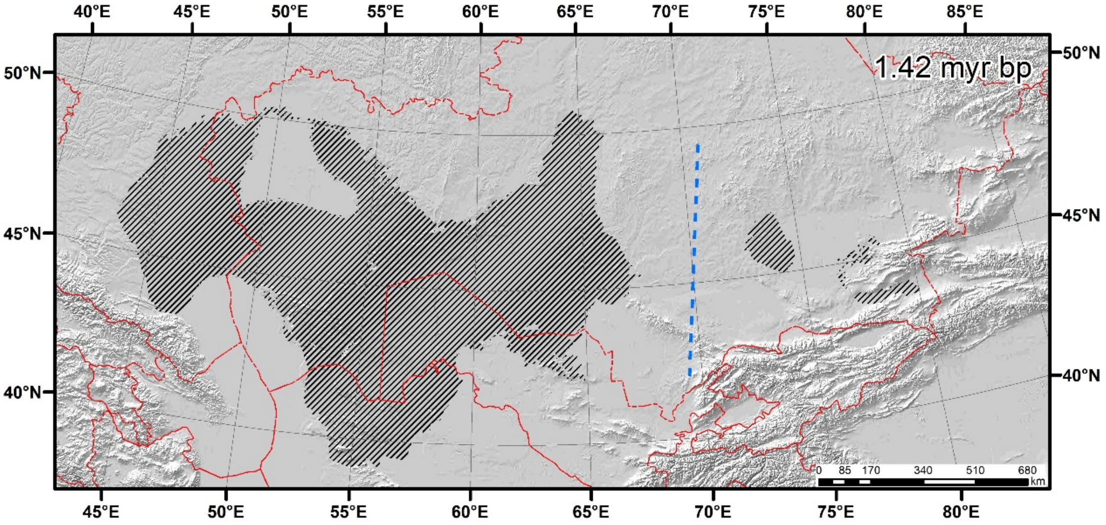

REFERENCES

1. Lebedev, V. S. *et al.* Molecular phylogeny and systematics of Dipodoidea: a test of morphology-based hypotheses. *Zool. Scr.* **42**, 231–249 (2013).
2. Wu, S. *et al.* Molecular and paleontological evidence for a post-cretaceous origin of rodents. *PLoS One* **7**, e46445 (2012).
3. Pisano, J. *et al.* Out of Himalaya: the impact of past Asian environmental changes on the evolutionary and biogeographical history of Dipodoidea (Rodentia). *J. Biogeogr.* **42**, 856–870 (2015).
4. Bannikova, A. *et al.* Genetic evidence for several cryptic species within the Scarturus elater species complex (Rodentia: Dipodoidea): when cryptic species are really cryptic. *Biol. J. Linn. Soc.* **126**, 16–39 (2019).
5. Moore, T. Y. *et al.* Multiple phylogenetically distinct events shaped the evolution of limb skeletal morphologies associated with bipedalism in the jerboas. *Curr. Biol.* **25**, 2785–2794 (2015).
6. Huchon, D. *et al.* Rodent phylogeny and a timescale for the evolution of glires: evidence from an extensive taxon sampling using three nuclear genes. *Mol. Biol. Evol.* **19**, 1053–1065 (2002).
7. Huchon, D. *et al.* Multiple molecular evidences for a living mammalian fossil. *Proc. Natl. Acad. Sci.* **104**, 7495–7499 (2007).
8. DeBry, R. W. Identifying conflicting signal in a multigene analysis reveals a highly resolved tree: The phylogeny of Rodentia (Mammalia). *Syst. Biol.* **52**, 604–617 (2003).
9. Meredith, R. W. *et al.* Impacts of the Cretaceous Terrestrial Revolution and KPg Extinction on Mammal Diversification. *Science (80-.).* **334**, 521–524 (2011).
10. Blanga-Kanfi, S. *et al.* Rodent phylogeny revised: analysis of six nuclear genes from all major rodent clades. *BMC Evol. Biol.* **9**, 71 (2009).
11. Huchon, D., Catzeflis, F. M. & Douzery, E. J. Molecular evolution of the nuclear von Willebrand factor gene in mammals and the phylogeny of rodents. *Mol. Biol. Evol.* **16**, 577–589 (1999).
12. Cheng, J. *et al.* Phylogeny, taxonomic reassessment and 'ecomorph' relationship of the Orientallactaga sibirica complex (Rodentia: Dipodidae: Allactaginae). *Zool. J.*

Linn. Soc. **192**, 185–205 (2021).

13. Liao, J. *et al.* Comparative phylogeography of Meriones meridianus, Dipus sagitta, and Allactaga sibirica: Potential indicators of the impact of the Qinghai-Tibetan Plateau uplift. *Mamm. Biol.* **81**, 31–39 (2016).
14. Dianat, M., Aliabadian, M., Darvish, J. & Akbarirad, S. Molecular phylogeny of the Iranian Plateau five-toed jerboa, Allactaga (Dipodidea: Rodentia), inferred from mtDNA. *Mammalia* **77**, (2013).
15. Mohammadi, S., Afonso, S., Adibi, M. A., Melo-Ferreira, J. & Campos, R. A new and highly divergent mitochondrial lineage in the small five-toed jerboa, Allactaga elater, from Iran (Mammalia: Rodentia). *Zool. Middle East* **62**, 206–211 (2016).
16. Moshtaghi, S., Darvish, J., Mirshamsi, O. & Mahmoudi, A. Cryptic species diversity in the genus Allactaga (Rodentia: Dipodidae) at the edge of its distribution range. *Folia Zool.* **65**, 142–147 (2016).
17. Montgelard, C., Bentz, S., Tirard, C., Verneau, O. & Catzeflis, F. M. Molecular Systematics of Sciurognathi (Rodentia): The Mitochondrial Cytochrome b and 12S rRNA Genes Support the Anomaluroidea (Pedetidae and Anomaluridae). *Mol. Phylogenet. Evol.* **22**, 220–233 (2002).
18. Çolak, R. *et al. Türkiye'de Yayılış Gösteren Allactaga (Mammalia: Rodentia) Türlerinin Taksonomisi ve Filogenisi*. (2018).
19. Kryštufek, B. *et al.* Mitochondrial sequences point on a cryptic species in five-toed jerboas, subgenus Paralactaga. *Mammalia* **77**, (2013).
20. Shenbrot, G. *et al.* A new recent genus and species of three-toed jerboas (Rodentia: Dipodinae) from China: A living fossil? *J. Zool. Syst. Evol. Res.* **55**, 356–368 (2017).
21. Lebedev, V. S. *et al.* Phylogeographical study reveals high genetic diversity in a widespread desert rodent, Dipus sagitta (Dipodidae: Rodentia). *Biol. J. Linn. Soc.* **123**, 445–462 (2018).
22. Boratynski, Z., Brito, J. C. & Mappes, T. The origin of two cryptic species of African desert jerboas (Dipodidae: Jaculus). *Biol. J. Linn. Soc.* **105**, 435–445 (2012).
23. Luo, G., Ding, L. & Liao, J. The complete mitochondrial genome of Stylodipus

telum (Rodentia: Dipodidae) and its phylogenetic analysis. *Mitochondrial DNA Part A* **27**, 2568–2569 (2016).

24. Sambrook, J., Fritsch, E. F. & Maniatis, T. *Molecular cloning: a laboratory manual*. (Cold Spring Harbor, 1989).
25. Lebedev, V. S. *et al.* Phylogeny and evolutionary history of birch mice Sicista Griffith, 1827 (Sminthidae, Rodentia): Implications from a multigene study. *J. Zool. Syst. Evol. Res.* jzs.12279 (2019) doi:10.1111/jzs.12279.
26. Yang, D. Y., Eng, B., Waye, J. S., Dudar, J. C. & Saunders, S. R. Improved DNA extraction from ancient bones using silica-based spin columns. *Am. J. Phys. Anthropol.* **105**, 539–543 (1998).
27. Hall, T. A. BioEdit: a user-friendly biological sequence alignment editor and analysis program for Windows 95/98/NT. *Nucleic Acids Symp. Ser.* **41**, 95–98 (1999).
28. Swofford, D. L. PAUP*: Phylogenetic analysis using parsimony (* and other methods), version 40b10. (2003).
29. Nguyen, L.-T., Schmidt, H. A., von Haeseler, A. & Minh, B. Q. IQ-TREE: A Fast and Effective Stochastic Algorithm for Estimating Maximum-Likelihood Phylogenies. *Mol. Biol. Evol.* **32**, 268–274 (2015).
30. Kalyaanamoorthy, S., Minh, B. Q., Wong, T. K. F., von Haeseler, A. & Jermiin, L. S. ModelFinder: fast model selection for accurate phylogenetic estimates. *Nat. Methods* **14**, 587–589 (2017).
31. Minh, B. Q., Nguyen, M. A. T. & von Haeseler, A. Ultrafast approximation for phylogenetic bootstrap. *Mol. Biol. Evol.* **30**, 1188–1195 (2013).
32. Guindon, S. *et al.* New algorithms and methods to estimate maximum-likelihood phylogenies: assessing the performance of PhyML 3.0. *Syst. Biol.* **59**, 307–321 (2010).
33. Ronquist, F. *et al.* MrBayes 3.2: efficient bayesian phylogenetic inference and model choice across a large model space. *Syst. Biol.* **61**, 539–542 (2012).
34. Rambaut, A., Drummond, A. J., Xie, D., Baele, G. & Suchard, M. A. Posterior summarization in Bayesian phylogenetics using Tracer 1.7. *Syst. Biol.* **67**, 901–904 (2018).

35. Kumar, S. & Gadagkar, S. R. Disparity Index: a simple statistic to measure and test the homogeneity of substitution patterns between molecular sequences. *Genetics* **158**, 1321–1327 (2001).
36. Tamura, K., Dudley, J., Nei, M. & Kumar, S. MEGA4: Molecular Evolutionary Genetics Analysis (MEGA) Software Version 4.0. *Mol. Biol. Evol.* **24**, 1596–1599 (2007).
37. Heled, J. & Drummond, A. J. Bayesian inference of species trees from multilocus data. *Mol. Biol. Evol.* **27**, 570–580 (2010).
38. Stephens, M., Smith, N. J. & Donnelly, P. A new statistical method for haplotype reconstruction from population data. *Am. J. Hum. Genet.* **68**, 978–989 (2001).
39. Librado, P. & Rozas, J. DnaSP v5: a software for comprehensive analysis of DNA polymorphism data. *Bioinformatics* **25**, 1451–1452 (2009).
40. Mirarab, S. *et al.* ASTRAL: genome-scale coalescent-based species tree estimation. *Bioinformatics* **30**, i541–i548 (2014).
41. Fujisawa, T. & Barraclough, T. G. Delimiting species using single-locus data and the generalized mixed uule coalescent approach: a revised method and evaluation on simulated data sets. *Syst. Biol.* **62**, 707–724 (2013).
42. Marshall, C. R. A simple method for bracketing absolute divergence times on molecular phylogenies using multiple fossil calibration points. *The American Naturalist*, **171**, 726-742 (2008).
43. Solow, A. R. Estimation of stratigraphic ranges when fossil finds are not randomly distributed. *Paleobiology* **29**, 181–185 (2003).
44. Marshall, C. R. Using confidence intervals to quantify the uncertainty in the end-points of stratigraphic ranges. *Paleontol. Soc. Pap.* **16**, 291–316 (2010).
45. Qiu, Z.D., Wang, X.M. & Li, Q. Faunal succession and biochronology of the Miocene through Pliocene in Nei Mongol (Inner Mongolia). *Vertebrata PalAsiatica* **44**, 165-181 (2006).
46. Li, Q. & Zheng, S. H. Note on four species of dipodids (Dipodidae, Rodentia) from the Late Miocene Bahe Formation, Lantian, Shaanxi. *Vertebrata PalAsiatica* **43**, 283-296 (in Chinese with English summary) (2005).
47. Zazhigin, V. S. & Lopatin, A. V. Evolution, phylogeny, and classification of

Dipodoidea. in *Systematics and Phylogeny of the Rodents and Lagomorphs* (eds. Agadzhanyan, A. K. & Orlov, V. N.) 50–52 (KMK (in Russian), 2000).

48. Tjutkova, L. A. & Kaipova, G. O. Late Pliocene and Eopleistocene micromammal faunas of southeastern Kazakhstan. *Acta Zool. Cracoviensia* **39**, 546–557 (1996).
49. Devyatkin, E. V. *et al. Late Cenozoic of Mongolia (Stratigraphy and Paleogeography)*. (Nauka Press (in Russian), 1989).
50. Sukhov, V. P. *Late Pliocene Small Mammals from Akkulaevo Localitiy in Bashkiria*. (Nauka Press (in Russian), 1970).
51. Elith, J. et al. A statistical explanation of MaxEnt for ecologists. Divers. Distrib. 17, 43–57 (2011).
52. Elith, J. *et al*. Novel methods improve prediction of species' distributions from occurrence data. Ecography. 29, 129–151 (2006).
53. Michaux, J. & Shenbrot, G. Family Dipodidae (Jerboas). in *Handbook of the mammals of the world, 7. Rodents II* (eds. Wilson, D. E., Lacher, T. E. & Mittermeier, R. A.) 62–100 (Lynx Edicions in association with Conservation International and IUCN, 2017).
54. Holden, M. E. & Musser, G. G. Family Dipodidae. in *Mammal Species of the World. A Taxonomic and Geographic Reference* (eds. Wilson, D. E. & Reeder, D. M.) 871–893 (Johns Hopkins University Press, 2005).
55. Shenbrot, G. I.*et al.* Mammals of the Fauna of Russia and Contiguous Countries. Dipodoid Rodents. (Nauka Press (in Russian), 1995).
56. Ognev, S. I. Mammals of the USSR and adjacent countries: Rodents (continued). Mammals of eastern Europe and northern Asia. (Akademiya Nauk SSSR (in Russian), 1948).
57. Vinogradov, B. S. Fauna of the USSR; Mammals, vol. 3, pt. 4. Jerboas. (Academy Nauk SSSR Publishing House (in Russian), 1937).
